# Endogenous regulation of the Akt pathway by the aryl hydrocarbon receptor (AhR) in lung fibroblasts

**DOI:** 10.1038/s41598-021-02339-3

**Published:** 2021-11-30

**Authors:** Fangyi Shi, Noof Aloufi, Hussein Traboulsi, Jean-François Trempe, David H. Eidelman, Carolyn J. Baglole

**Affiliations:** 1 Research Institute of the McGill University Health Centre, Montreal, QC Canada; 2 Department of Pathology, McGill University, Montreal, QC Canada; 3 Department of Medicine, McGill University, Montreal, QC Canada; 4 Department of Pharmacology & Therapeutics, McGill University, Montreal, QC Canada

**Keywords:** Biochemistry, Cell biology

## Abstract

The aryl hydrocarbon receptor (AhR) is a ligand-activated transcription factor known to mediate toxic responses to dioxin. However, the role of the AhR in the regulation of cellular physiology has only recently been appreciated, including its ability to control cell cycle progression and apoptosis by unknown mechanisms. We hypothesized that the AhR enhances the activation of the AKT serine/threonine kinase (Akt) pathway to promote cell survival. Utilizing AhR knock-out (*Ahr*^−/−^) and wild-type (*Ahr*^+/+^) mouse lung fibroblasts (MLFs), we found that *Ahr*^−/−^ MLFs have significantly higher basal Akt phosphorylation but that AhR did not affect Akt phosphorylation in MLFs exposed to growth factors or AhR ligands. Basal Akt phosphorylation was dependent on PI3K but was unaffected by changes in intracellular glutathione (GSH) or p85α. There was no significant decrease in cell viability in *Ahr*^−/−^ MLFs treated with LY294002—a PI3K inhibitor—although LY294002 did attenuate MTT reduction, indicating an affect on mitochondrial function. Using a mass spectrometry (MS)-based approach, we identified several proteins that were differentially phosphorylated in the *Ahr*^−/−^ MLFs compared to control cells, including proteins involved in the regulation of extracellular matrix (ECM), focal adhesion, cytoskeleton remodeling and mitochondrial function. In conclusion, *Ahr* ablation increased basal Akt phosphorylation in MLFs. Our results indicate that AhR may modulate the phosphorylation of a variety of novel proteins not previously identified as AhR targets, findings that help advance our understanding of the endogenous functions of AhR.

## Introduction

The aryl hydrocarbon receptor (AhR) is a ligand-activated transcription factor that belongs to the basic helix-loop-helix (bHLH)/Per-ARNT-Sim (PAS) superfamily^1^. The bHLH-PAS family is comprised of transcription factors that contain a bHLH domain followed by tandem PAS domains^2^. In the canonical AhR signaling pathway, activated AhR translocates to the nucleus where it dimerizes with AhR nuclear translocator (ARNT), a class II protein of the bHLH-PAS family, in order to bind to the xenobiotic responsive elements (XREs) on DNA. This classical genomic AhR signaling pathway can be activated by many exogenous and endogenous compounds that share similar structural and chemical properties^3^. The best characterized AhR ligands are synthetic halogenated aromatic hydrocarbons (HAHs). HAHs are chemicals that consist of benzene rings with one or more halogens (i.e., chlorine, fluorine, bromine, or iodine) attached. Dioxins are a class of chlorinated aryl hydrocarbons composed of two benzene rings connected through two oxygen atoms, with dioxin being commonly used as the synonym for 2,3,7,8-tetrachlorodibenzo-p-dioxin (TCDD), a metabolically-inert chemical that is a potent and well-characterized AhR ligand.

The AhR pathway was believed to be primarily involved in the sensing and subsequent metabolization of xenobiotic chemicals. Upon ligand binding, the conformation of the AhR changes, exposing the nuclear localization signal and causing translocation into the nucleus^4^. In the nucleus, the AhR exchanges its chaperones for the AhR nuclear translocation (ARNT) and binds to the XRE core sequence (5′-GCGTG-3′) near the promoters of target genes to induce transcription^5^. Many genes that are targeted by AhR encode for phase I and II proteins such as those in the cytochrome P450 family. After activation, AhR is exported from the nucleus to the cytoplasm and degraded through a proteasome-dependent mechanism. Activation of the canonical AhR signaling pathway is best-known for mediating the toxic effects of TCDD, such as hepatotoxicity and immunosuppression^6,7^.

The AhR can also regulate cell physiology processes such as extracellular matrix (ECM) formation, cell cycle progression, and gene expression independent of its activation by ligands^8^. This may involve crosstalk with other pathways to produce downstream effects not involved in the canonical AhR-XRE signaling. One of the potential pathways is the AKT serine/threonine kinase (Akt) pathway that is well-known for promoting cell survival, proliferation, and protein synthesis^9^. The canonical pathway leading to Akt activation is initiated by the binding of growth factors to receptor tyrosine kinases (RTKs)^10^. Once activated, these receptors activate class IA phosphoinositide 3-kinases (PI3Ks), heterodimers consisting of a p110 catalytic subunit and a p85 regulatory subunit. Activated PI3Ks then phosphorylate phosphatidylinositol to produce phosphatidylinositol 3,4,5-triphosphate (PIP3)^11^. This results in the colocalization of inactive Akt and the enzymes that phosphorylate it to the cell membrane, leading to subsequent Akt activation^10^. Akt has two main activating phosphorylation sites—the threonine 308 residue (T308) which is phosphorylated by phosphoinositide-dependent kinase-1 (PDK1) and the serine 473 residue (S473) which is phosphorylated by mammalian target of rapamycin complex 2 (mTORC2)^10^. Proteins that negatively regulate activation of the Akt pathway include phosphatase and tensin homolog (PTEN) which dephosphorylates PIP3 and PH domain leucine-rich repeat protein phosphatase (PHLPP) which dephosphorylates Akt^11,12^. Reactive oxygen species (ROS) also modulate the PI3K/Akt pathway by negatively regulating the activity of PTEN^13–15^.

Many of the downstream effects of Akt overlap with those of the AhR, including regulation of cell proliferation and survival. We have previously shown that the AhR protects lung cells against apoptosis^16^, oxidative stress^17^ as well as endoplasmic reticulum (ER) stress^18^, but whether this is due to AhR-dependent control over Akt is not known. Published information on the involvement of the AhR on the Akt pathway is conflicting. Wu et al*.* found that AhR reduced apoptosis in a mouse hepatoma cell line due to increased Akt activation^19^ whereas a recent in vivo study found that there was more Akt activity in *Ahr*^−/−^ mouse liver^20^. Given our lack of information on the mechanism by which the AhR exerts homeostatic control over cellular processes, we sought to evaluate if AhR promotes the activation of the Akt pathway in lung fibroblasts to upregulate pro-survival mechanisms.

## Materials and methods

### Chemicals

All chemicals were purchased from Sigma-Aldrich (St. Louis, MO) unless otherwise indicated. Recombinant mouse platelet-derived growth factor-BB (PDGF-BB) was purchased from STEMCELL Technologies (Cambridge, MA). LY294002 was purchased from Cell Signaling Technology (Danvers, MA).

### Cell culture

Primary MLFs were derived from *Ahr*^+/+^ and *Ahr*^−/−^ mice as previously described^21^. All animal procedures were approved by the McGill University Animal Care Committee (Protocol Number: 5933) and were carried out in accordance with the Canadian Council on Animal Care and ARRIVE guidelines^22^. Fibroblasts were cultured in Gibco MEM (Thermo Fisher Scientific) supplemented with 10% FBS (Wisent Bioproducts), 1X Gibco GlutaMAX (Thermo Fisher Scientific), 1X antibiotic–antimycotic solution (Wisent Bioproducts: #450115), and 0.05 mg/ml gentamycin sulfate solution (Wisent Bioproducts). Cells were maintained at 37 °C in humidified air with 5% CO_2_. Cells used in each experiment were within two passage difference and no fibroblasts exceeding passage ten were used. All cells were seeded at 10,000 cells/cm^2^, grown to approximately 80–90% confluence and cultured in serum-free medium for 18 h before conducting the experiments unless otherwise indicated.

### Preparation of cigarette smoke extract (CSE)

CSE was generated from filtered research-grade cigarettes (3R4F) obtained from the Kentucky Tobacco Research Council (Lexington, KT) as previously described^23,24^. Briefly, smoke from one cigarette was bubbled through 15 ml of serum-free medium and the optical density (OD) of the CSE was measured using a SmartSpec Plus spectrophotometer (Bio-Rad Laboratories, Hercules, CA). An OD of 0.65 at 320 nm was considered to represent 100% CSE. CSE was then passed through a Thermo Scientific™ Titan3™ syringe filter with a pore size of 0.45 µm and diluted to 2% CSE using serum-free medium.

### Cellular protein extraction and quantification

Total cellular protein was extracted using Pierce RIPA buffer (Thermo Fisher Scientific) supplemented with 1X cOmplet Mini Protease Inhibitor Cocktail (Roche) and 1X PhosSTO Phosphatase Inhibitor Cocktail (Roche). Protein quantification was performed using a Thermo Scientific Pierce Bicinchoninic Acid (BCA) protein assay kit (Thermo Fisher Scientific) according to manufacturer’s instructions and the absorbance was measured with an iMark microplate reader (Bio-Rad Laboratories, Hercules, CA).

### Western blot

Twenty micrograms of total cellular protein were mixed with Laemmli SDS sample buffer (Alfa Aesar), subjected to SDS-PAGE, and electro-blotted onto Immun-Blot PVDF membranes (Bio-Rad Laboratories, Hercules, CA). Membranes were blocked with 5% Oxoid skim milk powder (Thermo Fisher Scientific) in 1X PBS and 0.1% Tween-20 (Thermo Fisher Scientific) for one hour at room temperature. After that, membranes were incubated on a rocking platform overnight at 4 °C with primary antibodies against phospho-Akt (Cell Signaling Technology 4060; 1:2000), total Akt (Cell Signaling Technology 2920; 1:1000), AhR (Enzo Life Sciences BML-SA210; 1:2000), phospho-GSK-3β (Cell Signaling Technology 5558; 1:1000), total GSK-3β (Cell Signaling Technology #12456; 1:1000), phospho-Akt substrates (Cell Signaling Technology 9614; 1:1000), p85α (Cell Signaling Technology 13666; 1:500), and PTEN (Cell Signaling Technology 9559, 1:1000). The membranes were then incubated for one hour at room temperature with the antibody against tubulin (Sigma-Aldrich T7816; 1:10000). The membranes were incubated with the secondary antibody for one hour at room temperature. The secondary antibodies were horseradish peroxidase-conjugated anti-rabbit IgG (Cell Signaling Technology 7074; 1:10000) and anti-mouse IgG (Cell Signaling Technology 7076; 1:10000). Detection of protein levels was performed using enhanced chemiluminescence (ECL) and the signals were captured using a ChemiDoc MP Imaging System (Bio-Rad Laboratories, Hercules, CA). Densitometric analysis was performed using Image Lab Software (Bio-Rad Laboratories, Hercules, CA) and target phospho-protein levels were normalized to the corresponding total protein levels. Full length blots are provided in the Supplementary information.

### Transfection

*Ahr*^+/+^ MLF were grown to 60–80% confluency and transfected with 100 nM of small interfering RNA (siRNA) against p85α (PI 3-kinase p85α siRNA (m): Santa Cruz Biotechnology #sc-36218) or non-targeting control siRNA (Control siRNA-D: Santa Cruz Biotechnology #sc-44232) using Lipofectamine RNAiMAX (Thermo Fisher Scientific) and siRNA transfection MEM (Santa Cruz Biotechnology) according to manufacturer's instructions. Cells were incubated in the transfection medium for one hour after which serum-containing medium was added. Twenty-four hours later, the cells were serum-starved for 18 h prior to experimentation.

### MTT assay

Equivalent numbers of fibroblasts were seeded in triplicate in 96-well plates. Cells were allowed to settle overnight then serum-starved for 18 h prior to conducting the experiments. Cells were either treated with 50 μM LY294002 alone or an equivalent volume of DMSO for 24 h. Following the treatments, 10 µl of 5 mg/ml MTT (in 1X PBS) was added to each well and the plates were incubated for 4 h at 37 °C. The plates were then centrifuged, and the media was removed. The precipitate in each well was dissolved by adding 200 µl of DMSO and the absorbance was read with a BioRad microplate reader at 510 nm.

### Cell viability

Equivalent numbers of fibroblasts were seeded in 6-well plates. Cells were allowed to settle overnight and serum-starved for 18 h prior the experiments. Cells were either treated with 50 μM LY294002 or DMSO for 24 h. Following treatments, cells were detached using Acutase, an equivalent volume of MEM was added, and the cells were collected to assess cell death. For the trypan blue assay, cells were mixed 1:1 with trypan blue (0.4%) and live and dead cells were counted using the Countess 3 FL (Invitrogen). For flow cytometry assessment of cell viability, cells were stained with propidium iodide (PI; BioLegend) in PBS immediately prior acquiring on a flow cytometry. After acquiring, the data were analyzed using FlowJo v10.2 software (https://www.flowjo.com/).

### Quantitative mass spectrometry (MS) for phosphoproteomics

*Ahr*^+/+^ and *Ahr*^−/−^ MLFs were cultured in T_175_ flasks and serum-starved for 18 h before collection. Cells were rinsed and harvested in 20 mM HEPES supplemented with 150 mM NaCl. The cell-containing solution was then centrifuged to remove the HEPES and resuspended in 8 M urea (pH 8) in 20 mM HEPES supplemented with protease and phosphatase inhibitors. The samples were sonicated briefly on ice and cell debris was cleared by centrifugation. The final samples contained pooled protein extracts utilizing cells from two different *Ahr*^+/+^ and *Ahr*^−/−^ mice. Equal amounts of protein were sent to Institut de recherches cliniques de Montréal (IRCM, Montréal) for mass spectrometry analysis. Approximately 290 µg of protein from each sample was digested by trypsin and the peptides labeled with tandem mass tag (TMT). The phosphopeptides were enriched using titanium dioxide (TiO_2_) and liquid chromatography with tandem mass spectrometry (LC–MS–MS) was performed. Peptide and protein identification were carried out using Mascot (Matrix Science) by searching against a *Mus musculus* reference proteome database (UniProt, the Universal Protein resource, http://www.uniprot.org). The results were then analyzed by Proteome Discoverer 2.4 (Thermo Scientific). Identified proteins with an abundance ratio adjusted p-value (*Ahr*^−/−^/*Ahr*^+/+^) less than 0.01 and a Mascot score greater than 20 were selected for further analysis. Peptide sequences of each identified protein were also manually inspected. Information used for functional annotation of each protein was obtained from the Uniprot database. To perform a protein–protein interaction network analysis, the Search Tool for Retrieval of Interacting Genes (STRING) (https://string-db.org) database was employed. Active interaction sources for constructing the network include text mining, experiments, databases, and co-expression. The species was limited to “*Mus musculus*” and a minimum required interaction score was set to 0.4.

### Statistical analysis

Statistical analysis was performed using Prism 6 (v. 6.02; GraphPad Software, San Diego, CA; https://www.graphpad.com/scientific-software/prism/). Statistical significance between two groups was analyzed by a two-tailed t-test. Statistical differences among the means of more than two groups were determined using one-way analysis of variance (ANOVA) followed by a Tukey’s multiple comparisons test. The interrelationships between more than two independent variables were assessed using two-way ANOVA followed by a Tukey’s post-hoc test unless otherwise indicated. In all cases, values with P < 0.05 were considered significantly different.

## Results

### Growth factor stimulation significantly induced Akt and GSK-3β phosphorylation independent of the AhR

**Figure 1:**
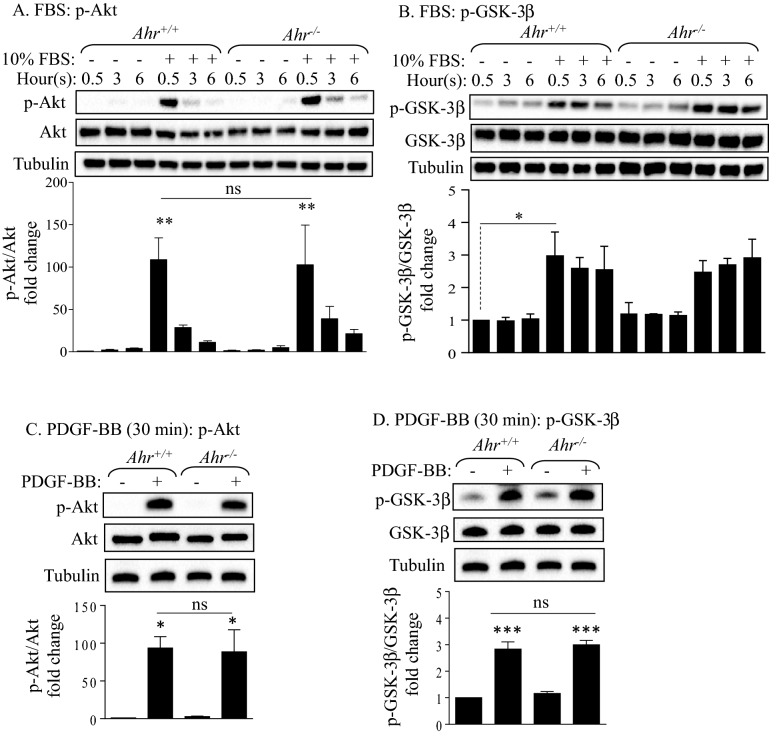
Effect of AhR expression on Akt and GSK-3β phosphorylation in MLFs exposed to 10% FBS or PDGF-BB. (**A**, **B**) *Ahr*^+/+^ and *Ahr*^−/−^ MLFs were either serum-starved for 18 h or maintained in 10% FBS. Cellular protein was extracted after 0.5, 3 and 6 h of changing either the serum-free or 10% FBS containing medium for Western blot analysis of (**A**) Akt and (**B**) GSK-3β phosphorylation. (**C**,** D**) Serum-starved *Ahr*^+/+^ and *Ahr*^−/−^ MLFs were treated with or without 20 ng/ml of PDGF-BB for 30 min and cellular protein was extracted for Western blot analysis of (**C**) Akt and (**D**) GSK-3β phosphorylation. Depicted blots are representative of three independent experiments. Densitometric values of the phosphorylated proteins were normalized to the values of their corresponding total proteins and were converted to fold changes relative to that of the control *Ahr*^+/+^ cells. Results are expressed as mean ± SEM (*p < 0.05; **p < 0.01; ***p < 0.001; ns, not statistically significant).

To determine the extent to which the AhR controls Akt phosphorylation, we first treated *Ahr*^+/+^ and *Ahr*^−/−^ MLFs with FBS and PDGF-BB, the latter being a growth factor well-known to induce Akt activation in fibroblasts^25^. Phosphorylation of GSK-3β, one of the key downstream targets of Akt^26^, was also evaluated. In both *Ahr*^+/+^ and *Ahr*^−/−^ MLFs, supplying the cells with 10% FBS significantly induced Akt phosphorylation after 30 min, which then gradually decreased over the 3- and 6-h time frame (Fig. 1A). However, the phosphorylation of Akt induced by FBS did not differ significantly between the *Ahr*^+/+^ and *Ahr*^−/−^ cells (Fig. 1A). FBS also increased GSK-3β phosphorylation, but there was no significant difference between *Ahr*^+/+^ and *Ahr*^−/−^ MLFs (Fig. 1B). PDGF-BB also induced rapid and significant Akt phosphorylation within 5 min (Fig. 1C). There was also a significant increase in GSK-3β phosphorylation by PDGF-BB (Fig. 1D). Although PDGF-BB significantly induced Akt and GSK-3β phosphorylation in MLFs, the level of induction did not differ between *Ahr*^+/+^ and *Ahr*^−/−^ cells (Fig. 1C,D). Thus, the AhR does not control Akt or GSK-3β phosphorylation by growth factors.

### CSE does not cause Akt and GSK-3β phosphorylation in MLFs

**Figure 2:**
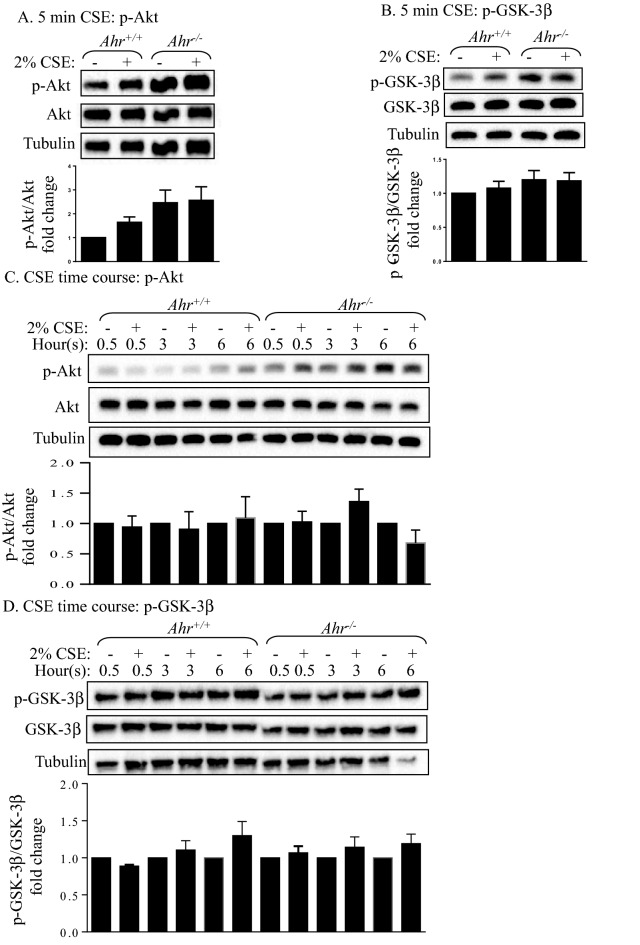
Effects of CSE on Akt and GSK-3β phosphorylation. (**A**, **B**) Serum-starved *Ahr*^+/+^ and *Ahr*^−/−^ MLFs were treated with or without 2% CSE for 5 min and cellular protein was collected for western blot analysis of (**A**) Akt and (**B**) GSK-3β phosphorylation. (**C**, **D**) *Ahr*^+/+^ and *Ahr*^−/−^ MLFs were treated with or without 2% CSE for 0.5, 3, and 6 h, and cellular protein was collected for Western blot analysis of (**C**) Akt and (**D**) GSK-3β phosphorylation. Blots are representative of (**A**, **B**) four or (**C**, **D**) three independent experiments. Densitometric values of the phosphorylated proteins were normalized to the values of their corresponding total proteins and are converted to fold changes relative to that of (**A**, **B**) the control *Ahr*^+/+^ cells or (**C**, **D**) untreated control groups at each time point. Results are expressed as mean ± SEM.

Cigarette smoke is a complex mixture of toxicants that contains numerous AhR ligands. Our laboratory previously found that AhR protects lung fibroblasts against CSE-induced apoptosis and ER stress^16,18,27^. Since Akt is part of a pro-survival pathway^26^, we next evaluated whether the AhR activates Akt in response to cigarette smoke. In response to 2% CSE for 5 min, there was a slight but non-significant increase in Akt phosphorylation in *Ahr*^+/+^ cells, but the phosphorylation of Akt in *Ahr*^+/+^ cells never exceeded that of *Ahr*^−/−^ cells (Fig. 2A). There was also no change in GSK-3β phosphorylation (Fig. 2B). Exposure to 2% CSE for up to 6 h also did not significantly change the phosphorylation of either Akt or GSK-3β in MLFs (Fig. 2C,D). Therefore, we conclude that exposure to cigarette smoke does not impact the Akt pathway in MLFs.

### B[*a*]p and CH-223191 does not affect Akt phosphorylation

**Figure 3:**
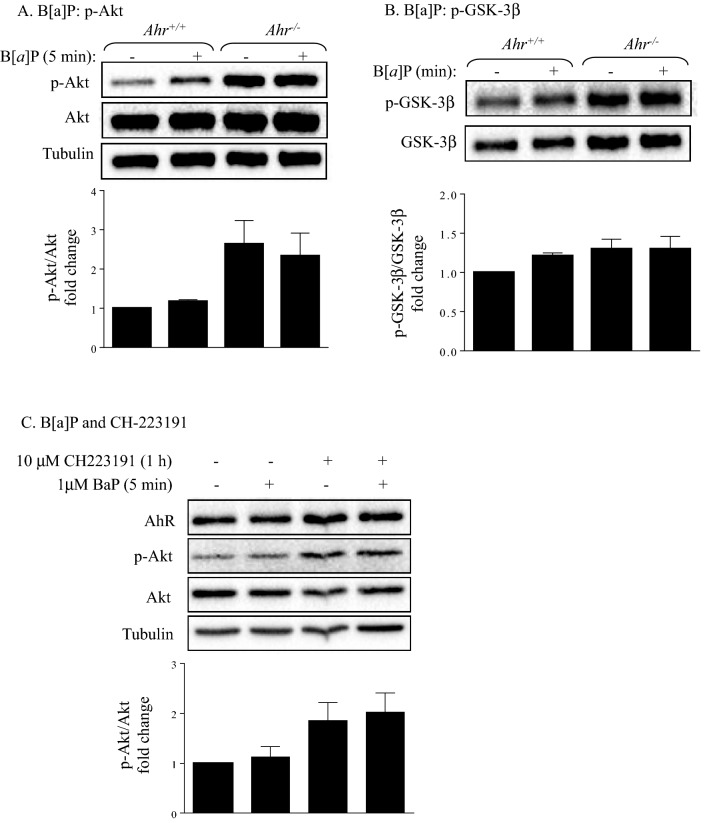
Effect of AhR activation on Akt and GSK-β phosphorylation**. **(**A**, **B**) Serum-starved *Ahr*^+/+^ and *Ahr*^−/−^ MLFs were either treated with 1 μM B[*a*]P or the vehicle control (DMSO) for 5 min and cellular protein was collected for western blot analysis of (**A**) Akt and (**B**) GSK-3β phosphorylation. (**C**) Serum-starved *Ahr*^+/+^ MLFs were treated with 10 μM CH-223191 or DMSO for 1 h followed by 5 min co-treatment with 1 μM B[*a*]P or DMSO and cellular protein was collected for western blot analysis of Akt phosphorylation. Blots are representative of (**A**, **B**) three or (**C**) five independent experiments. Densitometric values of the phosphorylated proteins were normalized to the values of their corresponding total proteins and were converted to fold changes relative to that of the control *Ahr*^+/+^ cells. Results are expressed as mean ± SEM.

Next, we evaluated whether activation of the AhR by B[*a*]P, a component of cigarette smoke and an established AhR agonist^28,29^, caused Akt and GSK-3β phosphorylation. We also included an AhR antagonist CH-223191 in our experiments with *Ahr*^+/+^ MLFs, as B[*a*]P can affect cellular pathways not involving AhR. We found that exposure to B[*a*]P (1 μM) did not affect Akt phosphorylation in *Ahr*^+/+^ MLFs (Fig. 3A). There was no change in GSK-3β phosphorylation with B[*a*]P exposure (Fig. 3B). Finally, we pretreated cells with CH-223191 (10 μM) followed by co-treatment with B[*a*]P (1 μM). However, CH-223191 did not significantly affect Akt phosphorylation (Fig. 3C). We conclude that AhR activation by classic ligands or AhR inhibition by CH-223191 has little direct impact on the Akt pathway in MLFs.

### The AhR controls basal phosphorylation of Akt

**Figure 4:**
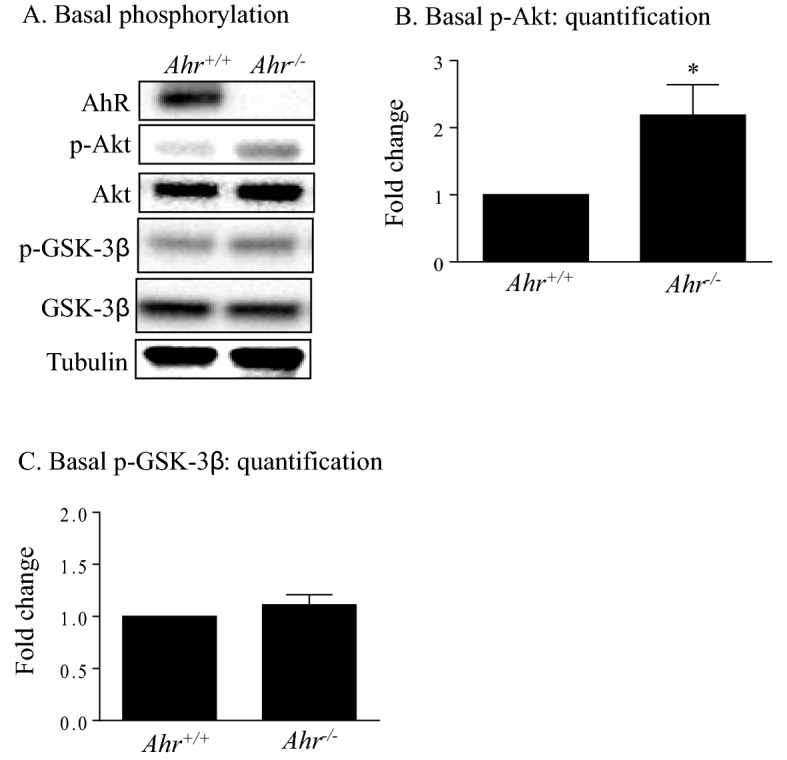
Basal Akt and GSK-3β phosphorylation in *Ahr*^+/+^ and *Ahr*^−/−^ MLFs. *Ahr*^+/+^ and *Ahr*^−/−^ MLFs were serum-starved for 18 h and cellular protein was collected for Western blot analysis of Akt and GSK-3β phosphorylation. (**A**) Representative western blot is shown and is representative of six independent experiments. (**B**, **C**) Densitometric analysis of basal (**B**) Akt and (**C**) GSK-3β phosphorylation in *Ahr*^+/+^ and *Ahr*^−/−^ MLFs. Densitometric values of phosphorylated proteins were normalized to that of their corresponding total proteins and are expressed as fold changes relative to that of the *Ahr*^+/+^ group. Results are expressed as mean ± SEM (*p < 0.05).

During these experiments, it became evident that *Ahr*^−/−^ MLFs may have higher basal Akt phosphorylation than *Ahr*^+/+^ cells (see Fig. 3). Akt phosphorylation was therefore quantified in serum-starved *Ahr*^+/+^ and *Ahr*^−/−^ MLFs. In the absence of exogenous stimuli, there was significantly higher Akt phosphorylation in quiescent *Ahr*^−/−^ MLFs than *Ahr*^+/+^ MLF (Fig. 4A,B). However, the phosphorylation of GSK-3β at the basal level did not differ significantly between *Ahr*^+/+^ and *Ahr*^−/−^ cells (Fig. 4A,C). Collectively, these data suggest that the AhR controls Akt phosphorylation under basal conditions without impacting GSK-3β.

### Altered expression of key upstream signaling mediators does not control basal Akt phosphorylation

**Figure 5:**
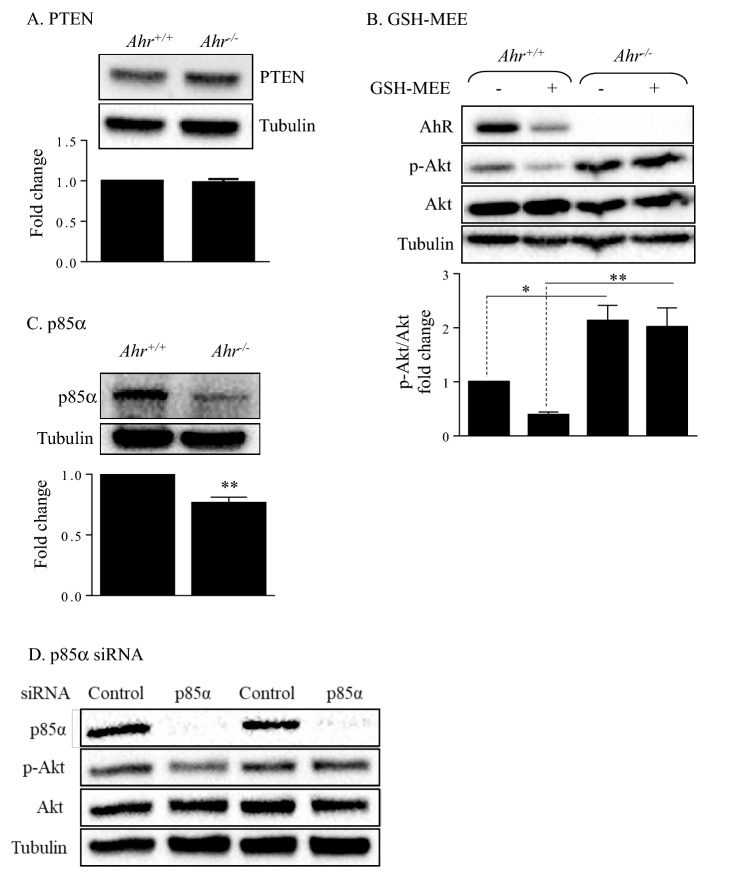
Expression of PTEN and p85α protein in *Ahr*^+/+^ *a*nd *Ahr*^−/−^ MLFs. (**A**, **C**) *Ahr*^+/+^ and *Ahr*^−/−^ MLF were serum-starved for 18 h and cellular protein was collected for Western blot analysis of (**A**) PTEN and (**C**) p85α expression. Depicted blots are representative of four independent experiments. Densitometric values were normalized to tubulin and are expressed as fold change relative to that of the *Ahr*^+/+^ groups. Results are expressed as mean ± SEM (*p < 0.05). (**B**) Serum-starved *Ahr*^+/+^ *a*nd *Ahr*^−/−^ MLF were treated with 2 mM GSH-MEE or the vehicle control (H_2_O) for 1 h and cellular protein was collected for Western blot analysis of Akt phosphorylation. Depicted blots are representative of three independent experiments. (**D**) *Ahr*^+/+^ MLFs were transfected with non-targeting control siRNA or siRNA against p85α. Transfected cells were serum-starved for 18 h and cellular protein was collected for western blot analysis of Akt phosphorylation. Representative western blot is shown.

Next, we evaluated whether AhR controls the expression of key upstream regulators of Akt activity. We first examined the expression of PTEN, the major negative regulator of the PI3K/Akt pathway^30^. However, there was similar PTEN protein expression in *Ahr*^+/+^ and *Ahr*^−/−^ MLFs (Fig. 5A). Given that PTEN inhibition by ROS can indirectly promote activation of the PI3K/Akt pathway^31^, and that we have shown the AhR controls oxidative stress in MLFs^17^, we speculated that higher ROS may be responsible for higher basal Akt phosphorylation in *Ahr*^−/−^ MLFs. To test this, we pretreated the cells with glutathione reduced ethyl ester (GSH-MEE) to boost intracellular GSH levels^24^ and evaluated Akt phosphorylation. GSH-MEE treatment reduced Akt phosphorylation in *Ahr*^+/+^ cells but had no effect on *Ahr*^−/−^ MLFs (Fig. 5B). Finally, we assessed p85α, a highly-expressed PI3K regulatory subunit that modulates PI3K pathway activation^32^. Under baseline conditions, p85 can dimerize with the PI3K catalytic subunit to stabilize and inhibit its catalytic activity^33^. Hence, we predicted that increased Akt phosphorylation in *Ahr*^−/−^ MLFs was associated with reduced p85α expression. Although we observed a significant reduction in p85α protein level in the *Ahr*^−/−^ MLFs (Fig. 5C), knocking down p85α using siRNA did not affect Akt phosphorylation (Fig. 5D). Thus, decreased p85α expression alone does not increase Akt phosphorylation in MLFs.

### PI3K activity is required for basal phosphorylation of Akt and maintenance of mitochondrial function in *Ahr*^−/−^ MLFs

**Figure 6:**
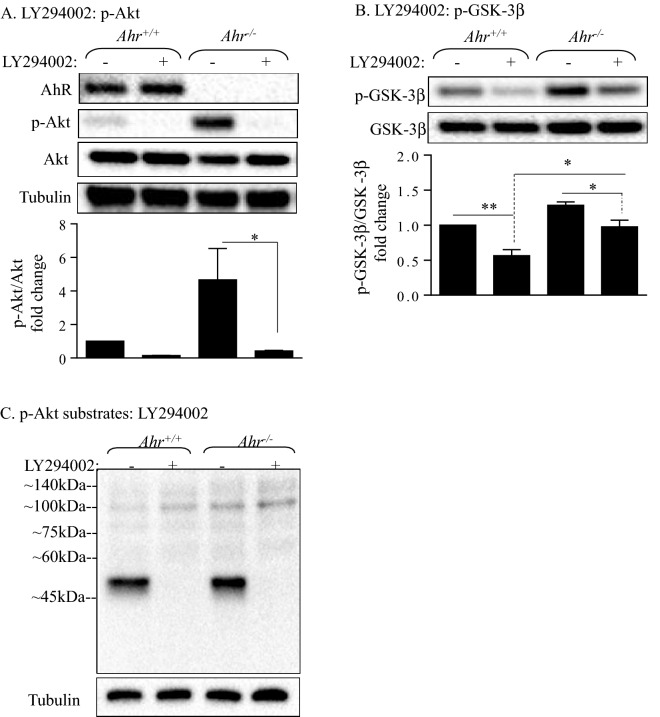
Effect of LY294002 the Akt pathway on *Ahr*^+/+^ and *Ahr*^−/−^ MLFs. (**A**, **B**) *Ahr*^+/+^ and *Ahr*^−/−^ MLFs were treated with either 50 μM LY294002 or the vehicle control (DMSO) for 2 h and cellular protein was collected for Western blot analysis of (**A**) Akt and (**B**) GSK-3β phosphorylation. Depicted blots are representative of three independent experiments. Results are expressed as mean ± SEM. (**C**) Serum-starved *Ahr*^+/+^ *a*nd *Ahr*^−/−^ MLFs were treated with LY294002 for 2 h and cellular protein was collected for western blot analysis of Akt substrate phosphorylation. Depicted blots are representative of three independent experiments.

To investigate if AhR regulates basal Akt phosphorylation by a mechanism that is dependent on PI3K, we utilized the PI3K inhibitor LY294002^34–36^. In cells that were exposed to LY294002, there was a significant reduction in the basal phosphorylation status of Akt in *Ahr*^−/−^ MLFs (Fig. 6A). Here, LY294002 completely abrogated the heightened phosphorylation in *Ahr*^−/−^ MLFs. Note that LY294002 had no effect on total Akt levels. There was also a significant reduction in basal GSK-3β phosphorylation by LY294002 (Fig. 6B). As Akt phosphorylates its substrates at the serine/threonine residues preceded by arginine at positions-5 and -3^37^, we also utilized an antibody that recognizes the phosphorylation of Akt substrates at the minimum conserved motif Arg-Xaa-Xaa-Ser/Thr (Xaa: any amino acid). This antibody produced a strong signal near the 45 kDa region in untreated *Ahr*^+/+^ and *Ahr*^−/−^ MLFs (Fig. 6C). LY294002 eliminated this band (Fig. 6C).

**Figure 7:**
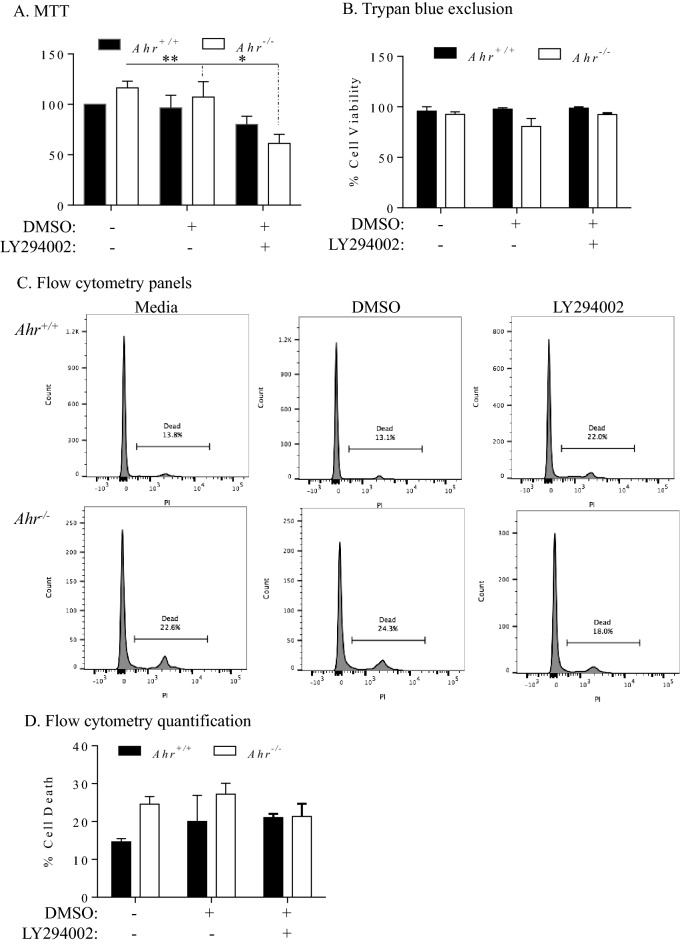
Effect of LY294002 on cell viability. *Ahr*^+/+^ and *Ahr*^−/−^ MLFs were either treated with LY294002 or an equivalent volume of DMSO for 24 h and viability was assessed by MTT assay, trypan blue exclusion and PI/flow cytometry (**A**) -There was a significant reduction in MTT reduction in *Ahr*^−/−^ MLFs treated with LY294002. Results are expressed as percent viability relative to the untreated *Ahr*^+/+^ cells and are presented as mean ± SEM (n = 5 independent experiments; *p < 0.05 and **p < 0.01 compared to corresponding DMSO control and media-only cells, respectively). (**B**) There was no significant reduction in cell viability upon treatment with LY294002 in either *Ahr*^+/+^ and *Ahr*^−/−^ MLFs. (**C**) Representative flow cytometry histograms of *Ahr*^+/+^ and *Ahr*^−/−^ MLFs treated with DMSO or LY294002; percentage of dead cells is indicated. (**D**) There was no significant increase in the percentage of dead cells in response to LY294002. Results are presented as the mean ± SEM (n = 2–5 independent experiments).

Finally, we tested whether LY294002 affected cell survival using three complementary techniques. First, we used MTT, a metabolic viability-based assay using the tetrazolium salt MTT. These data show that there was a significant reduction in cell viability only in *Ahr*^−/−^ MLFs upon inhibition of PI3K by LY294002 (Fig. 7A). These data suggest that basal Akt phosphorylation is important in controlling cell survival in MLFs. As MTT is reduced to a purple formazan crystal via mitochondrial dehydrogenases and is thus an indirect measure of cellular viability^38^, we complemented this with two additional direct measurements of viability. Using trypan blue exclusion, these data revealed that there was no significant difference in viability upon treatment with LY294002 in either *Ahr*^+/+^ and *Ahr*^−/−^ MLFs (Fig. 7B). Finally, we used flow cytometry and propidium iodide, a membrane impermeant dye that allows for the identification of dead cells. There was no significant different in propidium iodide uptake upon treatment with LY294002 (Fig. 7C,D). These data suggest that basal activation of PI3K-Akt pathway controls mitochondrial function in *Ahr*^−/−^ MLFs.

### AhR differently regulates protein phosphorylation in MLFs

**Figure 8:**
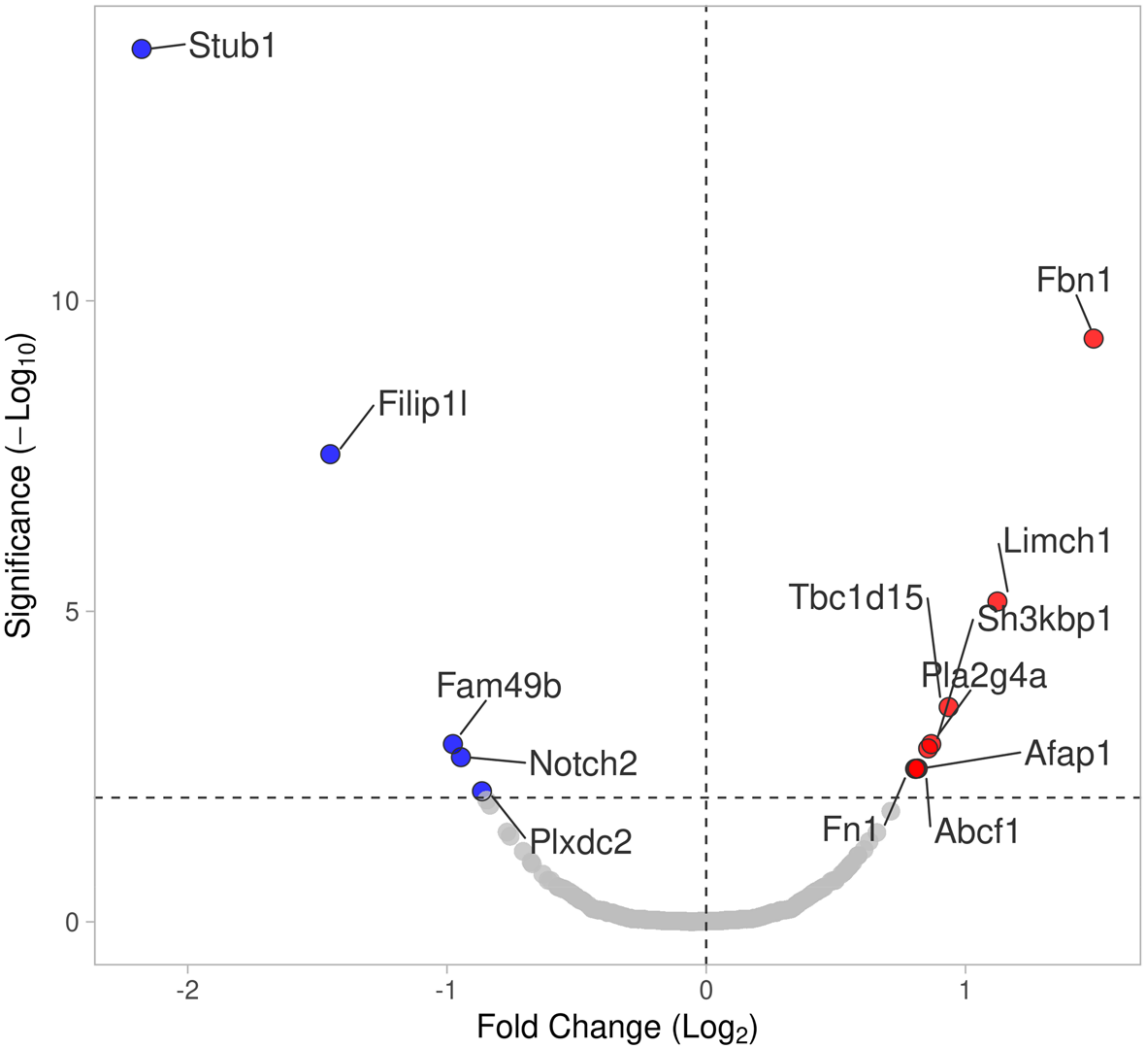
Volcano plot showing the relative levels of differentially phosphorylated proteins and their corresponding p values in the *Ahr*^+/+^ and *Ahr*^−/−^ MLFs identified by MS. Fold change indicates the intensity ratio between the *Ahr*^−/−^ and *Ahr*^+/+^ cells. The horizontal dashed line represents p = 0.01. Proteins more strongly phosphorylated in *Ahr*^−/−^ MLFs are indicated by the positive values and less phosphorylated by the negative values.

**Figure 9:**
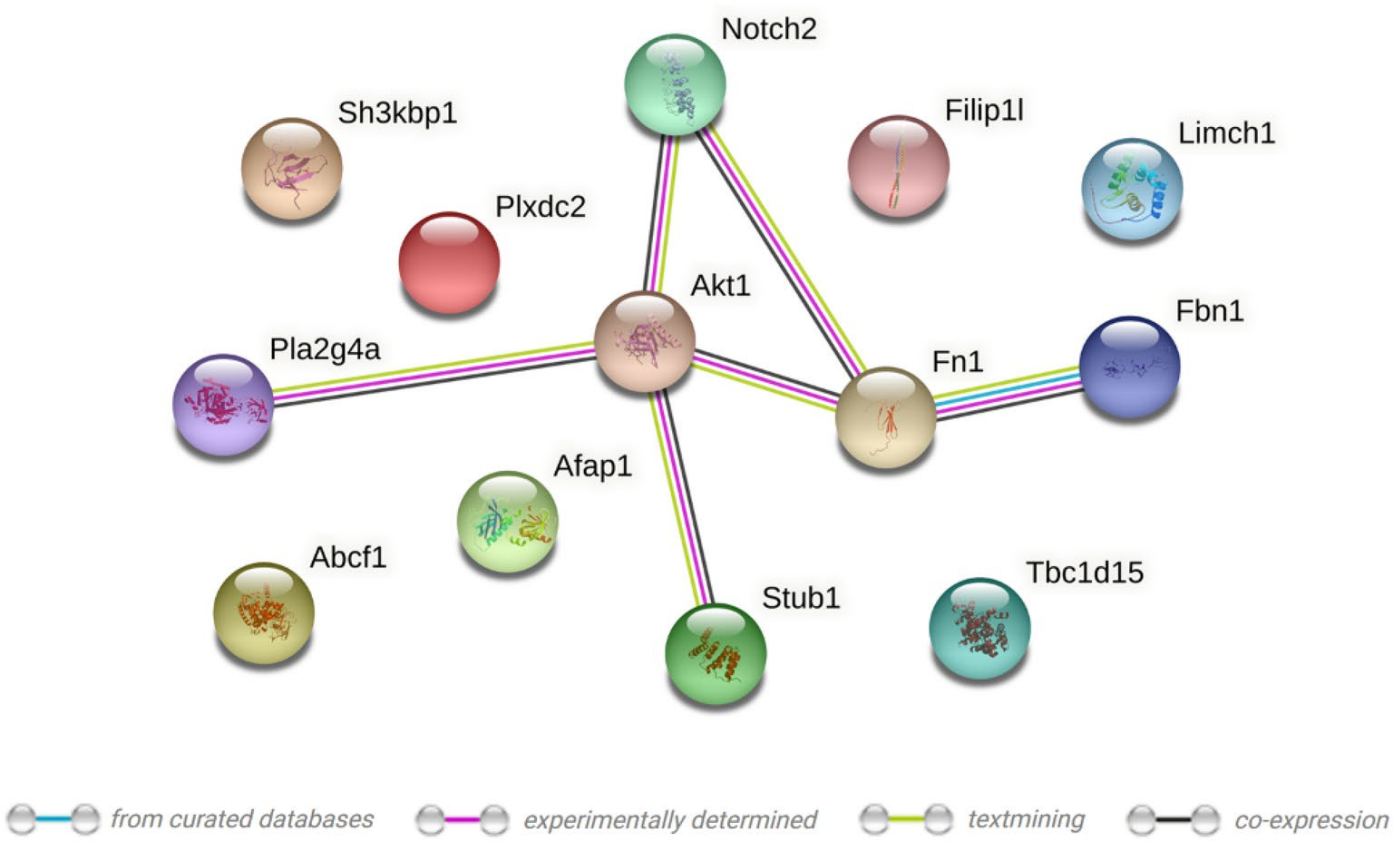
Potential interactions among differentially phosphorylated proteins and Akt. Colored nodes correspond to proteins and the edges represent the possibility of both functional and physical protein associations. Line color indicates the type of interaction evidence. Interaction scores: Akt1-Fn1: 0.902; Akt1-Stub1: 0.788; Akt1-Notch2: 0.644; Akt1-Pla2g4a: 0.504; Fn1-Fbn1: 0.986; Fn1-Notch2: 0.413. Cyrib from Table 1 was excluded from this analysis as no phosphorylated site was detected. The plot was generated using STRING.

To take a more comprehensive approach to evaluating phosphorylation of cellular proteins based on AhR expression, a quantitative MS-based proteomics analysis was conducted on serum-starved *Ahr*^+/+^ and *Ahr*^−/−^ MLFs. A total of 13 proteins with Mascot score greater than 20 were significantly up- or down-regulated (p < 0.01) in *Ahr*^−/−^ MLFs compared to *Ahr*^+/+^ MLFs (Fig. 8; Table 1). These proteins are involved in a diverse range of biological processes, including proliferation, apoptosis, differentiation, metabolism, autophagy, ubiquitination, phosphorylation and dephosphorylation, cytoskeleton remodeling, ECM organization, intracellular cargo transport, regulation of transcription and translation, protein folding, and modulation of various signal transduction pathways (Table 1). To predict potential functional interactions between the differentially phosphorylated proteins and Akt, the STRING database was used. The result indicates possible interactions between Fn1, Stub1, Notch2, and phospholipase A2 (PLA2) with Akt (Fig. 9). Collectively, our data support that the AhR plays a fundamental role in controlling basal Akt activity. The identification of novel proteins whose phosphorylation state is dependent on AhR expression further contributes novel data on the AhR in cellular physiology.

**Table 1:** List of differentially-regulated phosphorylated protein in *Ahr*^+/+^ (WT) and *Ahr*^−/−^ (KO) MLFs identified using MS.

Protein	Gene	Phosphorylated site(s)	Ratio: KO/WT	Functions
Fibrillin-1	*Fbn1*	[S2704]	2.817	Metal ion binding; ECM structural constituent; hormone activity regulation; a cell adhesion mediator
LIM and calponin homology domains-containing protein 1	*Limch1*	[S560]	2.178	Metal ion binding; actin binding; actin stress fibers assembly; focal adhesion stabilization; inhibition of cell migration
TBC1 domain family member 15	*Tbc1d15*	[S32; S201; S203; S205; S616]	1.912	Regulation of intracellular protein trafficking; acting as a GTPase activating protein for Rab family proteins
Phospholipase A2	*Pla2g4a*	[S429]	1.825	Ca^2+^-dependent phospholipase and lysophospholipase activities; membrane lipid remodeling and biosynthesis of lipid inflammatory mediators
SH3 domain-containing kinase-binding protein 1	*Sh3kbp1*	[S274; S631; S633]	1.81	Regulation of diverse signal transduction pathways; apoptosis; endocytosis; ubiquitin protein ligase binding; cell migration and cytoskeletal organization
Actin filament-associated protein 1	*Afap1*	[S669]	1.76	Regulation of cytoskeleton organization; an adapter molecule linking other proteins to the actin cytoskeleton
ATP-binding cassette sub-family F member 1	*Abcf1*	[S194]	1.753	mRNA translation initiation mediator; ATP binding and ATPase activity; RNA binding; ribosome binding
Fibronectin	*Fn1*	[S2294]	1.748	Regulation of cell shape, adhesion and migration; ECM organization; regulation of focal adhesion signaling
Plexin domain-containing protein 2	*Plxdc2*	[S456]	0.549	Transmembrane protein; Regulation of tumor angiogenesis
Neurogenic locus notch homolog protein 2	*Notch2*	[S2082]	0.519	Transmembrane receptor for membrane-bound ligands; transcriptional regulator; regulation of cell differentiation, proliferation and apoptotic programs
CYFIP-related Rac1 interactor B	*Cyrib*		0.508	Regulation of mitochondrial function; attenuation of actin filament polymerization, phagocytosis and cell migration; protection against infection
Filamin A-interacting protein 1-like	*Filip1l*	[S789; T992]	0.366	Antiangiogenic; negative regulation of proliferation and migration; positive regulation of apoptosis
STIP1 homology and U box-containing protein 1	*Stub1*	[S20]	0.221	Quality control for misfolded or incompletely synthesized proteins; modulation of the activity of several chaperone complexes; regulation of protein ubiquitination

## Discussion

The AhR is a ligand-activated transcription factor that was first identified as the binding protein for TCDD in 1976^39^. Since then, the role of AhR in mediating dioxin toxicity has been extensively investigated, while less is known about its endogenous functions. In the past few years, advances in molecular biology and technological innovations have led to the discovery of several non-canonical interactions between the AhR and other signaling proteins that may shed light on the physiological functions of AhR; an increasing number of studies have revealed the molecular and physiological significance of AhR involvement in cell cycle regulation, tissue differentiation, organ development, immune system function, and cell survival, among others. Our laboratory has shown that AhR has cytoprotective effects in pulmonary cells against the harmful effects of cigarette smoke, including in lung fibroblasts^16,18,27^. We utilize primary lung fibroblasts as these are a relevant lung cell type that is easily derived from tissue^21^. Many AhR-dependent effects in lung fibroblasts are conserved in other lung cell types such as epithelial and endothelial cells^16,40^. Importantly, AhR controls lung fibroblasts survival^16,17^. However, the mechanistic basis for AhR function in lung cells is unknown. We postulated that AhR may control upstream pathways important in cell survival, and in particular the Akt pathway, a fundamental pathway in cells of higher eukaryotes that governs cell growth, proliferation and survival^9^.

In response to known activators of either Akt (i.e., growth factors) or AhR (i.e., CSE and B[*a*]P), there was no difference in phosphorylation of Akt between the *Ahr*^+/+^ and *Ahr*^−/−^ cells. The lack of AhR-dependent regulation of Akt phosphorylation by growth factors (including serum) likely means that lower proliferation previously reported in lung fibroblasts caused by AhR deficiency^27^ is not mediated by the Akt pathway. Moreover, the lack of differentiatial regulation by AhR ligands is in contrast with previous studies showing induction of Akt phosphorylation in cells treated with CSE^41,42^. This discrepancy may be due to difference in CSE concentrations used between studies. It has also been reported that components in cigarette smoke, including B[*a*]P, induce rapid Akt activation^43,44^. Specifically, Bölck et al*.* found that 1 μM B[*a*]P significantly induced Akt phosphorylation in human immortalized (HaCaT) keratinocytes after 5 min of treatment^41^. However, the phosphorylation of Akt (S473) from exposure to B[*a*]P was unchanged in MLFs. One reason that could account for this discrepancy is that the Akt phosphorylation site evaluated by Bölck et al*.* was T308^44^, whereas that evaluated in our study was S473. It is therefore possible that these two sites are differently phosphorylated by the AhR depending on the conditions. Collectively, these results lead to the intriguing speculation that under homeostatic conditions in MLFs, the AhR regulates Akt phosphorylation in a way such that it prevents an exaggerated response while still allowing respiratory cells to respond quickly to the changing environment. This notion is further supported by the fact that basal Akt phosphorylation was significantly higher in *Ahr*^−/−^ cells, suggesting that the AhR exerts homeostatic control over Akt phosphorylation. Although this is inconsistent with previous studies showing that AhR promotes Akt activation^19,45^, our result is in accordance with a previous study that demonstrated that knocking-out AhR increased Akt phosphorylation in mouse liver^20^. The inconsistency between results in these different studies may be due to the fact that some studies have compared the cancer cells with their AhR-deficient counterparts. Specifically, cells used by Wu et al*.* were a low-AhR expressing variant derived from the parental murine (Hepa1c1c7) hepatoma cells^46^, which expresses approximately 10% of the wild-type AhR level^19,45^, whereas our study has compared primary lung fibroblasts from *Ahr*^+/+^ and *Ahr*^−/−^ mice. This suggests that the regulation of basal Akt activity by the AhR may be cell-type specific and/or be reflective of differences between primary and cancer cells.

The mechanism through which AhR regulates basal Akt activity is not known but we speculated could be due to changes in the expression or function of key upstream regulators such as PTEN or PI3K. However, the level of PTEN was not different between *Ahr*^+/+^ and *Ahr*^−/−^ cells. Another key regulator of the PI3K pathway is p85α^10^. Although p85α level was significantly decreased in the *Ahr*^−/−^ cells, knock-down of p85α did not affect basal Akt phosphorylation, suggesting that downregulation of p85α alone is not a cause of elevated Akt phosphorylation. As the activity of the PTEN and PI3K/Akt pathway can also be controlled by oxidative stress^47^, we evaluated whether GSH would decrease Akt phosphorylation. GSH is a tripeptide (l-γ-glutamyl-l-cysteinylglycine) synthesized by eukaryotic cells to protect against oxidative stress^48^. GSH contains a cysteine residue with an active thiol group, which can act as an antioxidant to reduce reactive oxygen and nitrogen species^48^. In this study, we used GSH-MEE, a lipophilic molecule that can be hydrolyzed by intracellular esterases to release GSH, thereby increasing intracellular GSH^49,50^. We have previously published that GSH-MEE boosts intracellular GSH levels in primary lung fibroblasts to reduce smoke-induced oxidative stress and apoptosis^24^. However, GSH only decreased Akt phosphorylation in *Ahr*^+/+^ cells, suggesting that ROS signaling plays a role in basal Akt activation in *Ahr*^+/+^ MLFs, whereas Akt activity in *Ahr*^−/−^ cells is insensitive to changes in GSH. As GSH-MEE is a lipophilic molecule that is converted to GSH intracellularly through enzymatic activity^51^, it is also possible that AhR deficiency alters the expression and/or function of these non-specific esterases such that GSH-MEE is uncleaved, thereby reducing its anti-oxidant potential.

To take a more comprehensive approach to understanding the AhR, we performed mass spectrometric analysis of differentially phosphorylated proteins between *Ahr*^+/+^ and *Ahr*^−/−^ cells. We identified several proteins that showed significant differences and are important in regulating cellular functions controlled by AhR. For example, the AhR regulates cell morphology, focal adhesion, and migration^52,53^. We found that many proteins regulating these processes were differentially phosphorylated between *Ahr*^+/+^ and *Ahr*^−/−^ MLFs; the protein with highest phosphorylation due to *Ahr* ablation was fibrillin-1, a major structural component of microfibrils^54^ that regulates the storage, release, and activation of extracellular TGF-β1, a cytokine that causes the differentiation of fibroblasts into myofibroblasts. Protein–protein interaction analysis further allowed us to identify potential interactions, some of which may play a role in the regulation of Akt phosphorylation, such as PLA2, Stub1, and Notch2.

Although we did not detect upregulated phosphorylation of well-characterized Akt substrates in *Ahr*^−/−^ MLFs by western blot and MS, we suspected that enhanced Akt phosphorylation in *Ahr*^−/−^ MLFs is associated with cell viability. To test this, we utilized the PI3K inhibitor LY294002^25^. Basal activation of Akt dependent on the PI3K, as the phosphorylation of Akt was eliminated by LY294002. Using MTT assay, it appeared that LY294002 significantly reduced viability in *Ahr*^−/−^ MLFs, suggesting that increased Akt phosphorylation is required by *Ahr*^−/−^ MLFs for cell survival. MTT conversion requires active metabolism via mitochondrial enzymes. Given that we have previously published on the importance of AhR in maintaining mitochondrial health^16^, we utilized two additional complementary cell viability assays. Using trypan blue exclusion and propidium iodide incorporation with flow cytometry, treatment with LY294002 did not alter cell viability. Thus, LY294002 is affecting the mitochondria without causing increased cell death. The PI3K/Akt pathway influences mitochondrial function, including control over metabolic enzymes^15^. These new data suggest that an AhR-Akt pathway is involved in the maintenance of mitochondrial health. It is possible that this is occurring through phosphorylation of novel proteins identified by our mass spectroscopy analysis such as FAM49B (*Cyrib*)^55^. Furthermore, LY294002 inhibits PI3K by acting on the ATP binding site of the enzyme^25^ although LY294002 may non-specifically interact with other proteins. For example, LY294002 also binds to and may perturb the functions of casein kinase 2 (CK2), GSK-3β, and mammalian target of rapamycin (mTOR)^56^, which can be a limitation in the interpretation of data obtained from this study. Moreover, LY294002 can bind to the AhR and antagonize its activation by TCDD^57^. Although the binding of LY294002 to AhR does not activate its DNA-binding ability at the concentrations used in this study^57^, LY294002 may still interfere with the physiological function of AhR that could confound interpretation of the results. It is also noteworthy that LY294002 eliminated basal Akt phosphorylation but not that of GSK-3β. Although GSK-3β is one of the best-established targets of Akt^26^, these data suggest that there are additional signaling mechanisms that regulate GSK-3β phosphorylation in MLFs.

**Figure 10:**
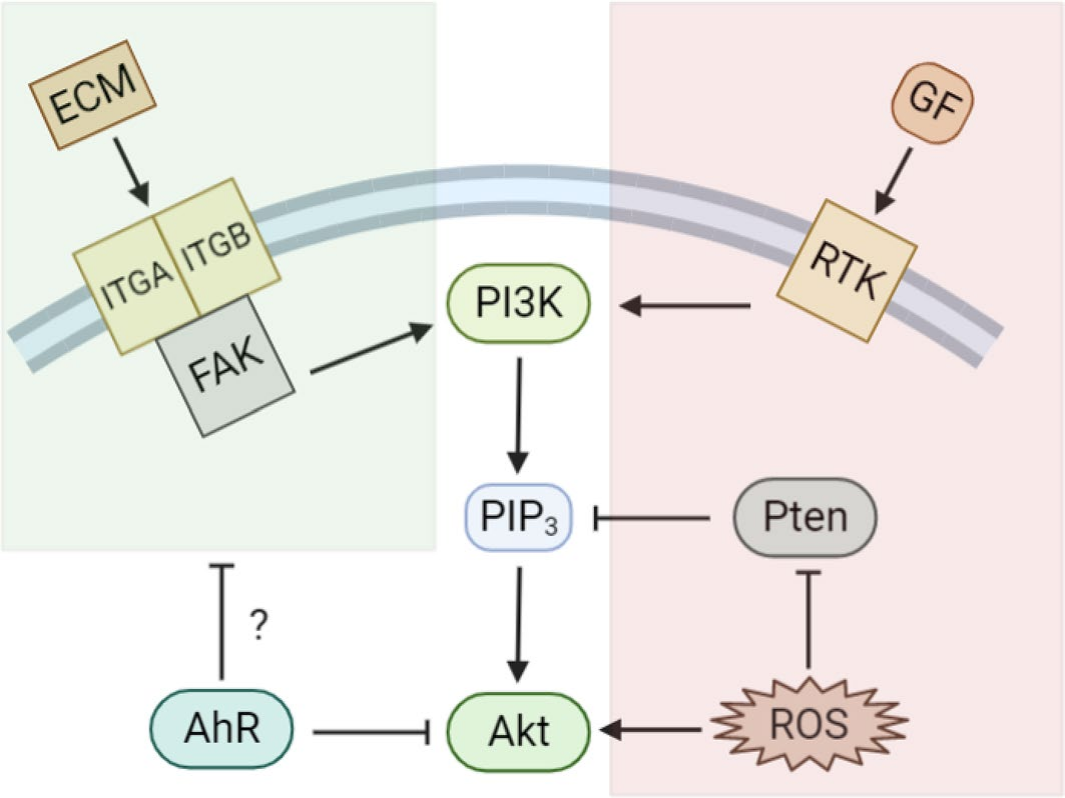
Depiction of potential AhR-dependent Akt activating pathways. In this study, we show that the presence of AhR reduced basal Akt phosphorylation. PI3K/Akt signaling pathway is activated by many types of cellular stimuli. Besides the canonical growth factor-mediated RTK/PI3K/Akt pathway, interactions between integrin and ECM can also transduce signals into the cell by activating the FAK/PI3K/Akt pathway; AhR may affect Akt phosphorylation through the regulation of these pathways. The red shaded area corresponds to the pathways not likely to be involved in AhR-dependent downregulation of Akt phosphorylation. The green shaded area represents the potential pathway by which AhR could modulate Akt phosphorylation. *ECM* extracellular matrix, *GF* growth factors, *ITGA* integrin subunit alpha, *ITGB* integrin subunit beta, *FAK* focal adhesion kinase, *RTK* receptor tyrosine kinase, *PI3K* phosphoinositide 3-kinase, *AhR* aryl hydrocarbon receptor, *PIP*_*3*_ phosphatidylinositol 3,4,5-triphosphate, *Akt* AKT serine/threonine kinase, *Pten* phosphatase and tensin homolog, *ROS* reactive oxygen species.

In conclusion, we report for the first time that the AhR controls the phosphorylation of Akt under basal conditions but not in response to growth factors, CSE, or AhR ligands. We also show that basal Akt activation in MLFs was dependent on the PI3K pathway. Our findings raise intriguing questions on how AhR regulates Akt-related pathways in mammalian cells. Nevertheless, the finding that absence of the AhR results in differential phosphorylation of a number of proteins, including fibrillin and fibronectin, gave rise to the speculation that increased Akt phosphorylation in *Ahr*^−/−^ MLFs was associated with dysregulation of the ECM (Fig. 10). Given that lung fibroblasts are important producers of the ECM raises the intriguing possibility of a role for the AhR in fibrotic diseases. Further molecular investigations into the proteomic changes caused by AhR ablation will lead to the identification of novel interactions between AhR and other cellular pathways and advance our understanding of the physiological functions of AhR in the respiratory system.

## Supplementary Information

Supplementary Information.

## Data Availability

Data are available from the corresponding author upon request.
