# Supplementary material for: Endogenous regulation of the Akt pathway by the aryl hydrocarbon receptor (AhR) in lung fibroblasts
Source: Sci Rep. 2021 Nov 30;11:23189. doi: 10.1038/s41598-021-02339-3 (PMC8632926; doi:10.1038/s41598-021-02339-3)

Online Supplement

Endogenous regulation of the Akt pathway by the aryl hydrocarbon receptor (AhR) in lung fibroblasts

Fangyi Shi, Noof Aloufi, Hussein Traboulsi, Jean-François Trempe, David H. Eidelman and Carolyn J. Baglole

Figure 1A. FBS: p-Akt

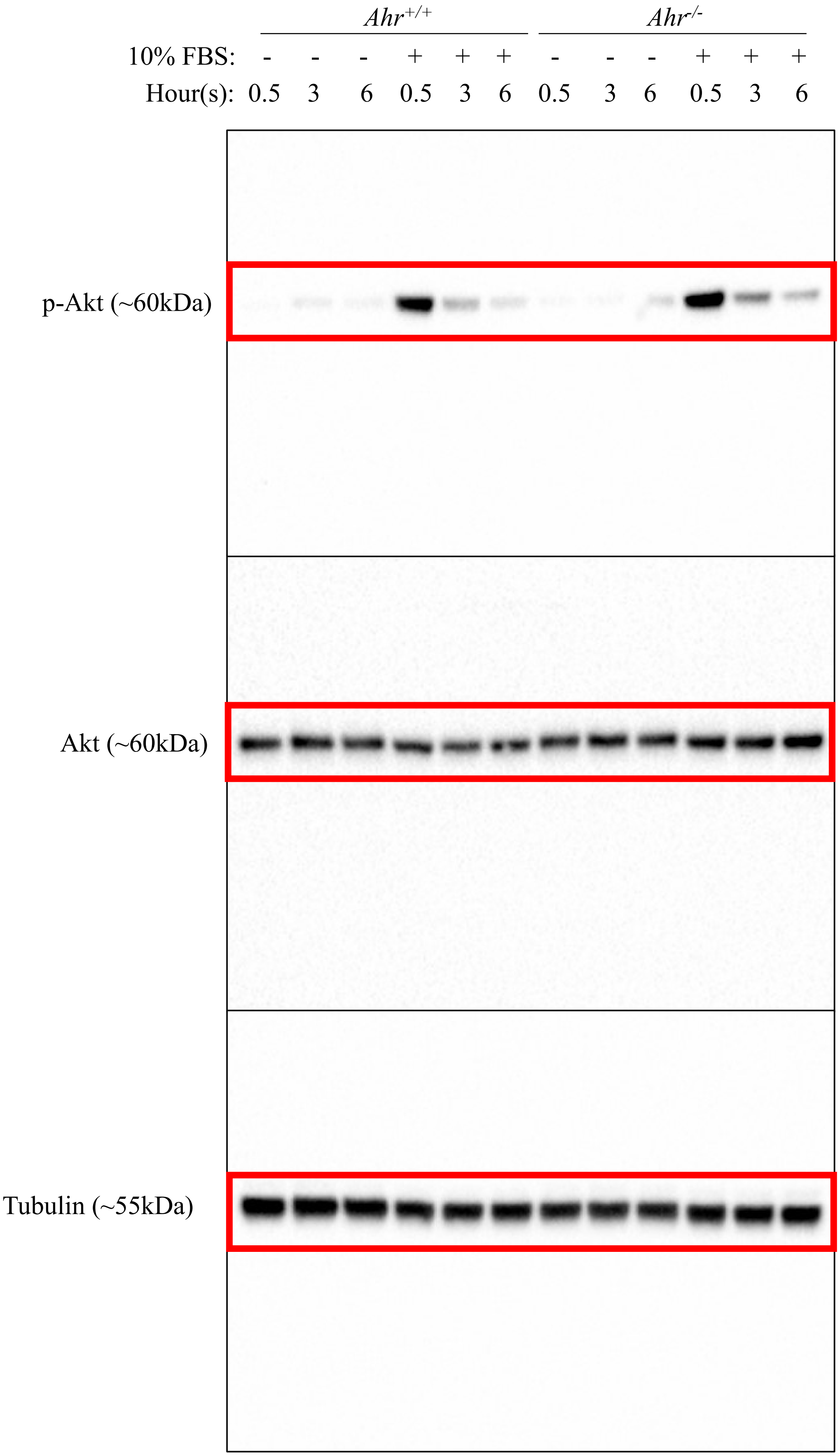

Figure 1A. FBS: p-Akt (second exposure)

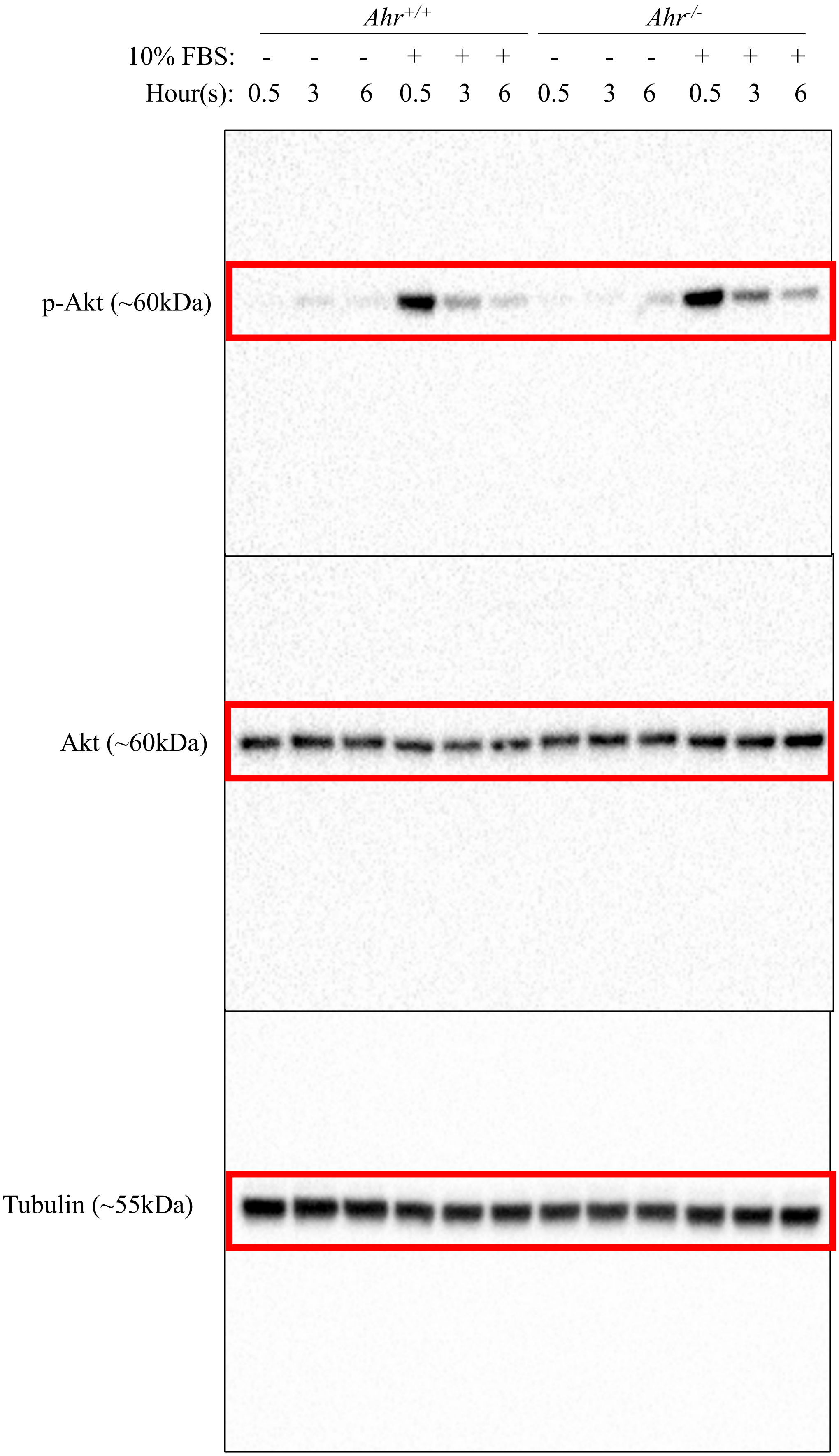

Figure 1A. FBS: p-Akt (protein ladder)

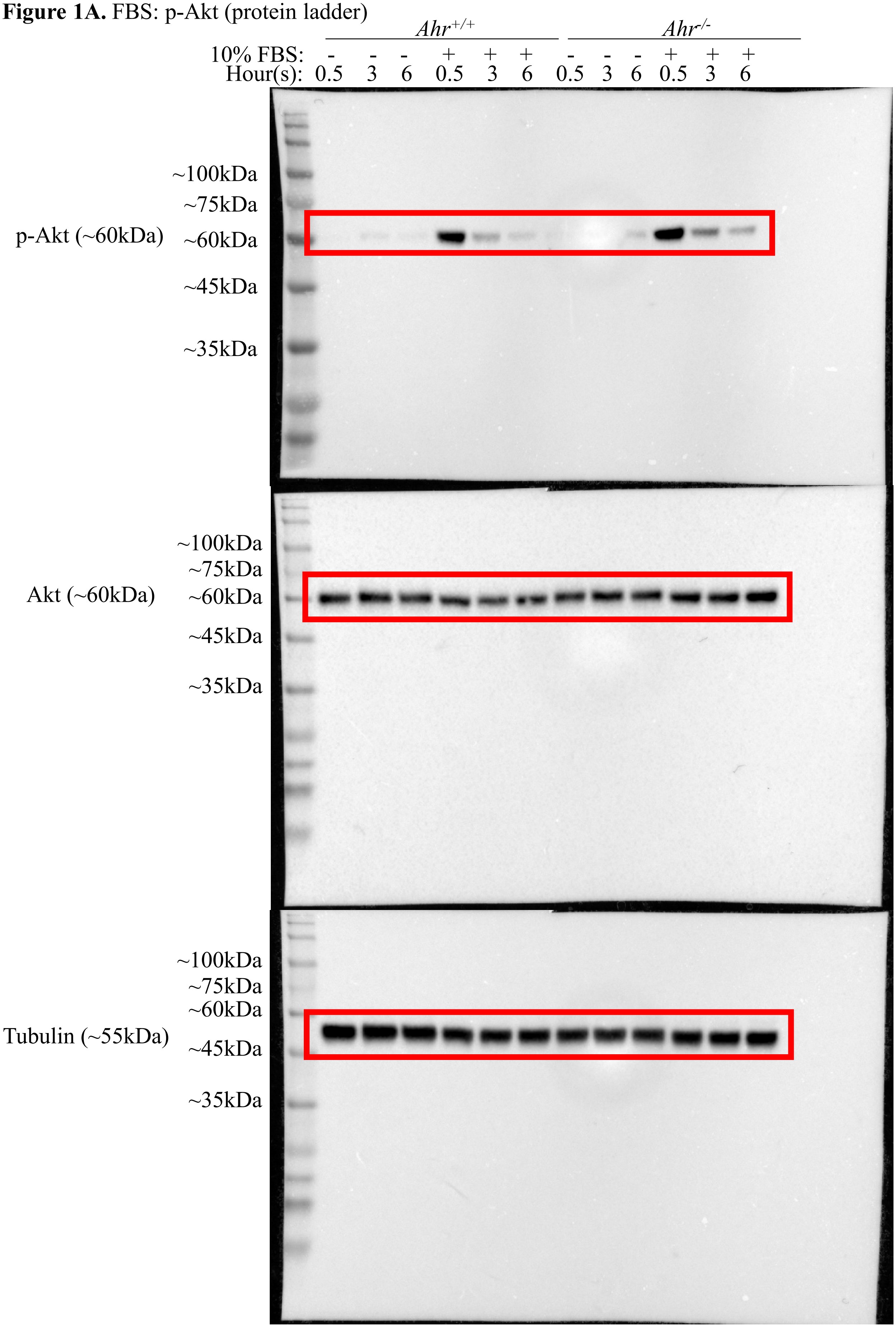

Figure 1B. FBS: p-GSK3β

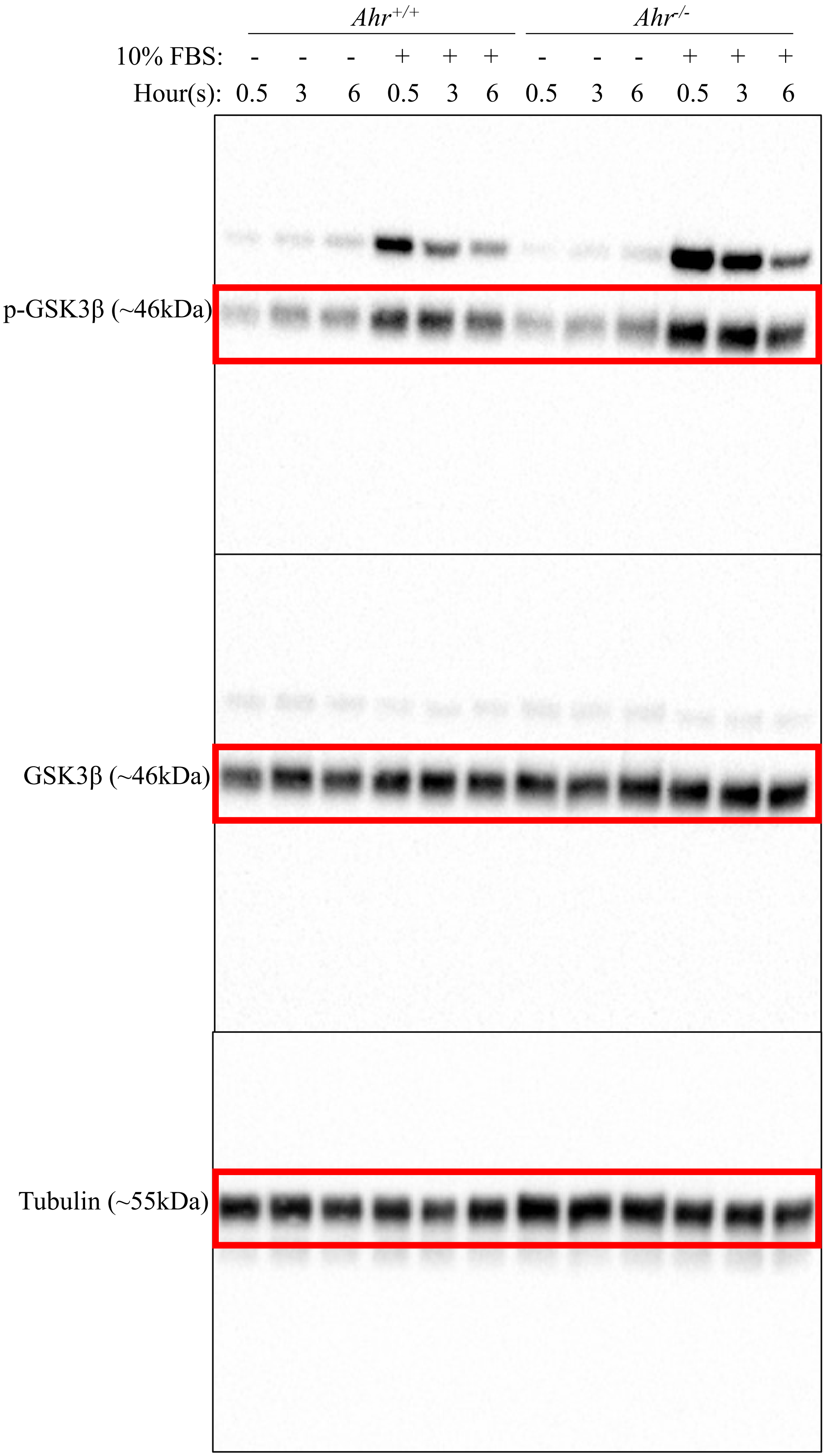

Figure 1B. FBS: p-GSK3β (second exposure)

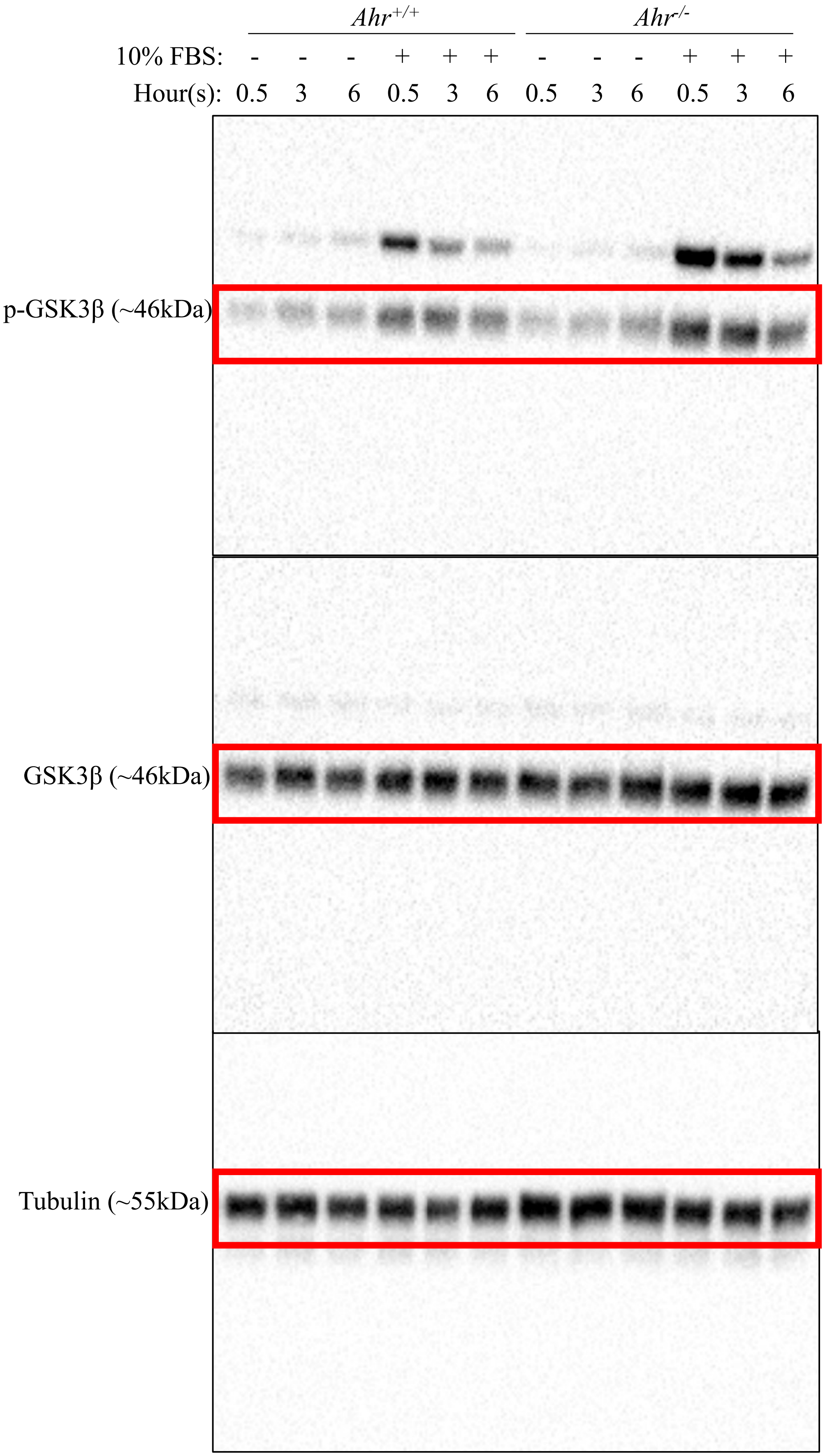

Figure 1B. FBS: p-GSK3β (protein ladder)

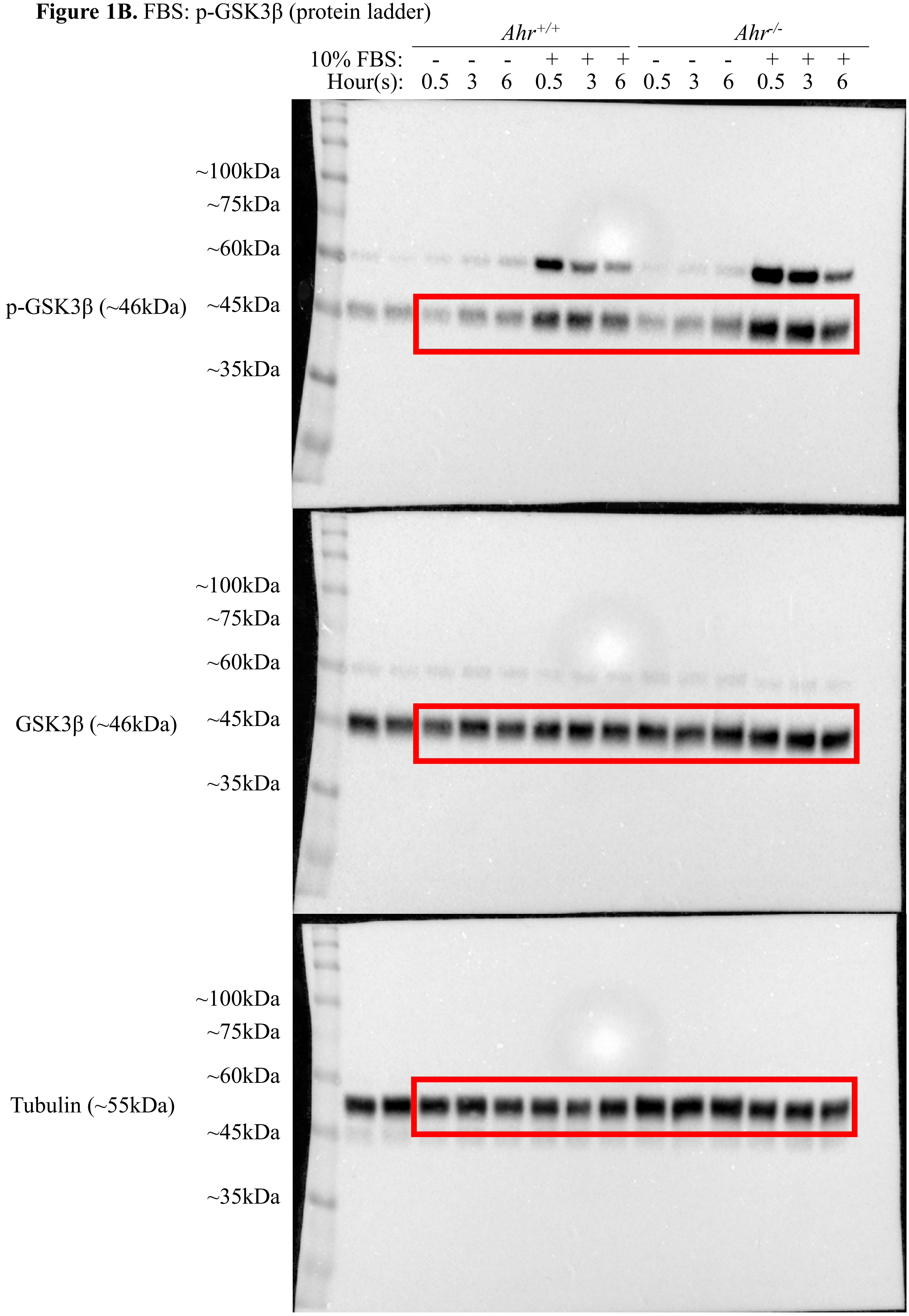

Figure 1C. PDGF-BB (30 min): p-Akt

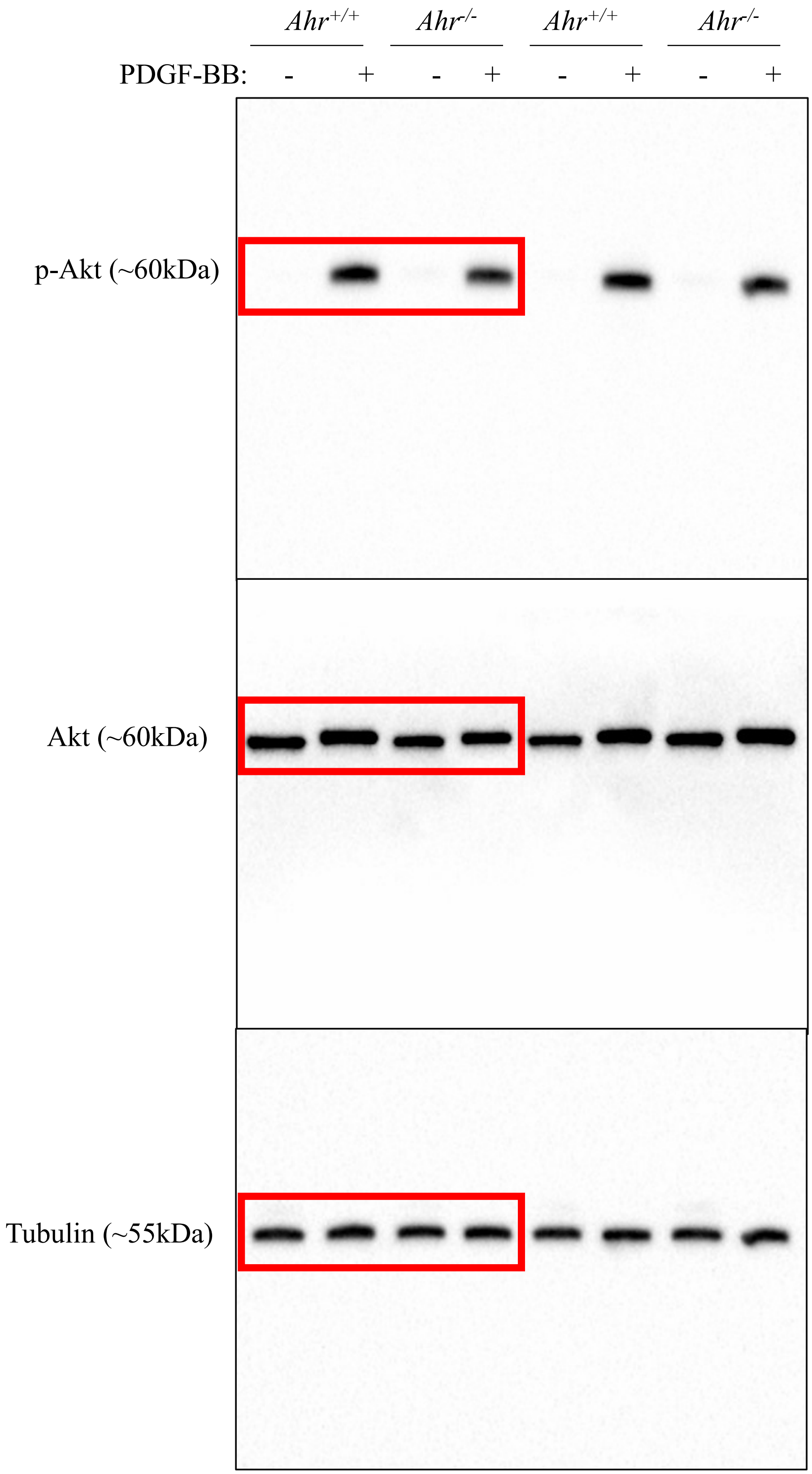

Figure 1C. PDGF-BB (30 min): p-Akt (second exposure)

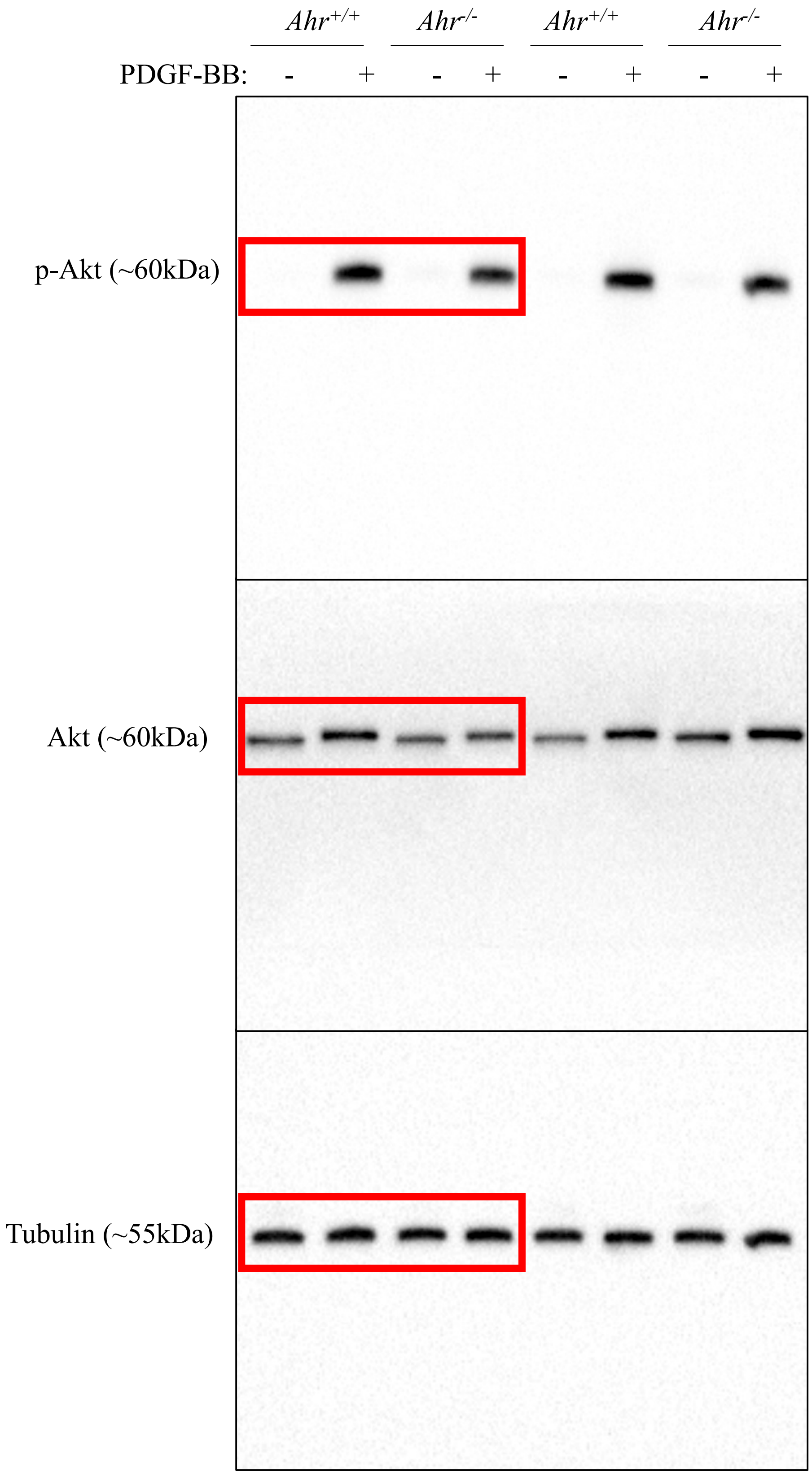

Figure 1C. PDGF-BB (30 min): p-Akt (protein ladder)

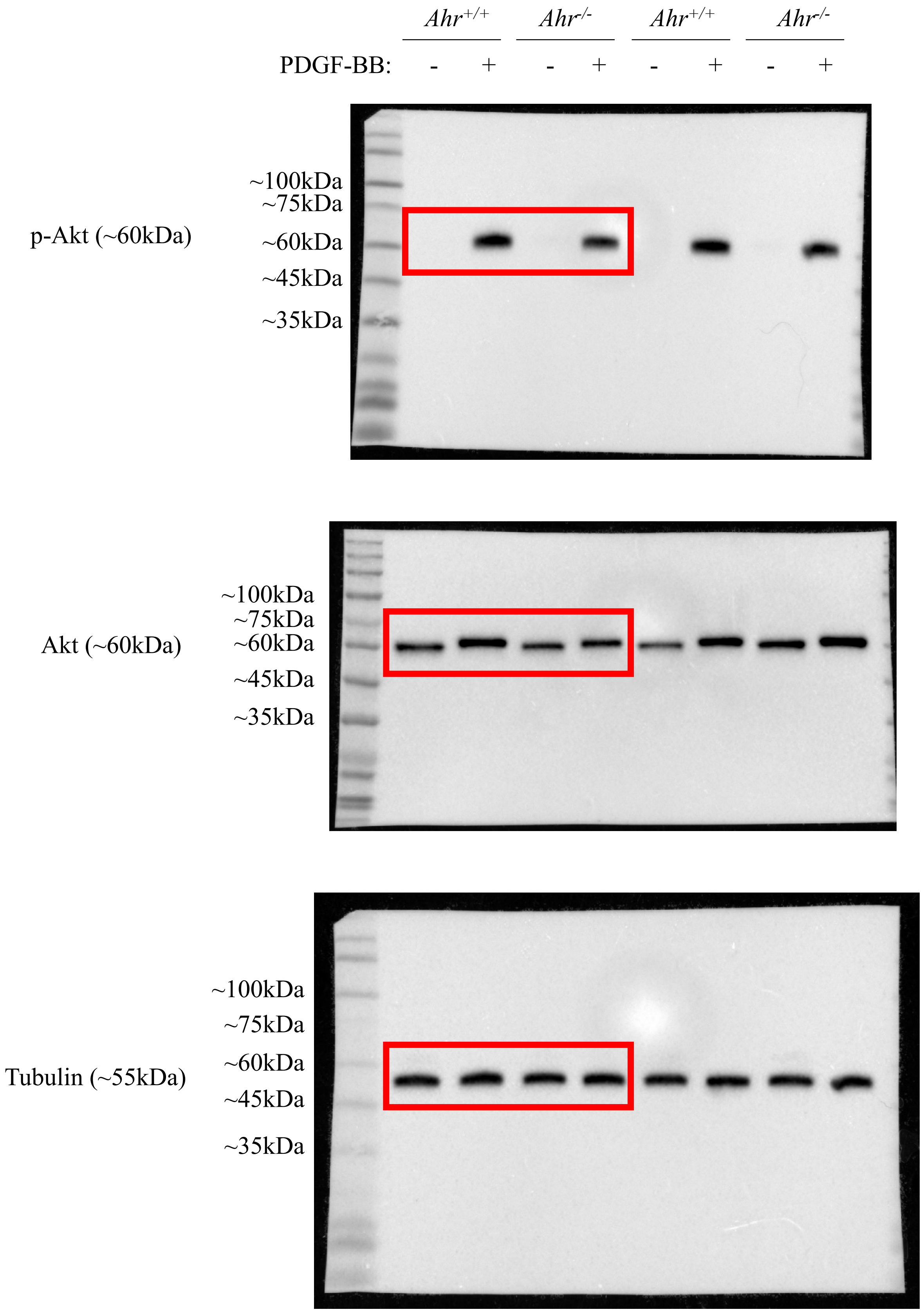

Figure 1D. PDGF-BB (30 min): p-GSK3β

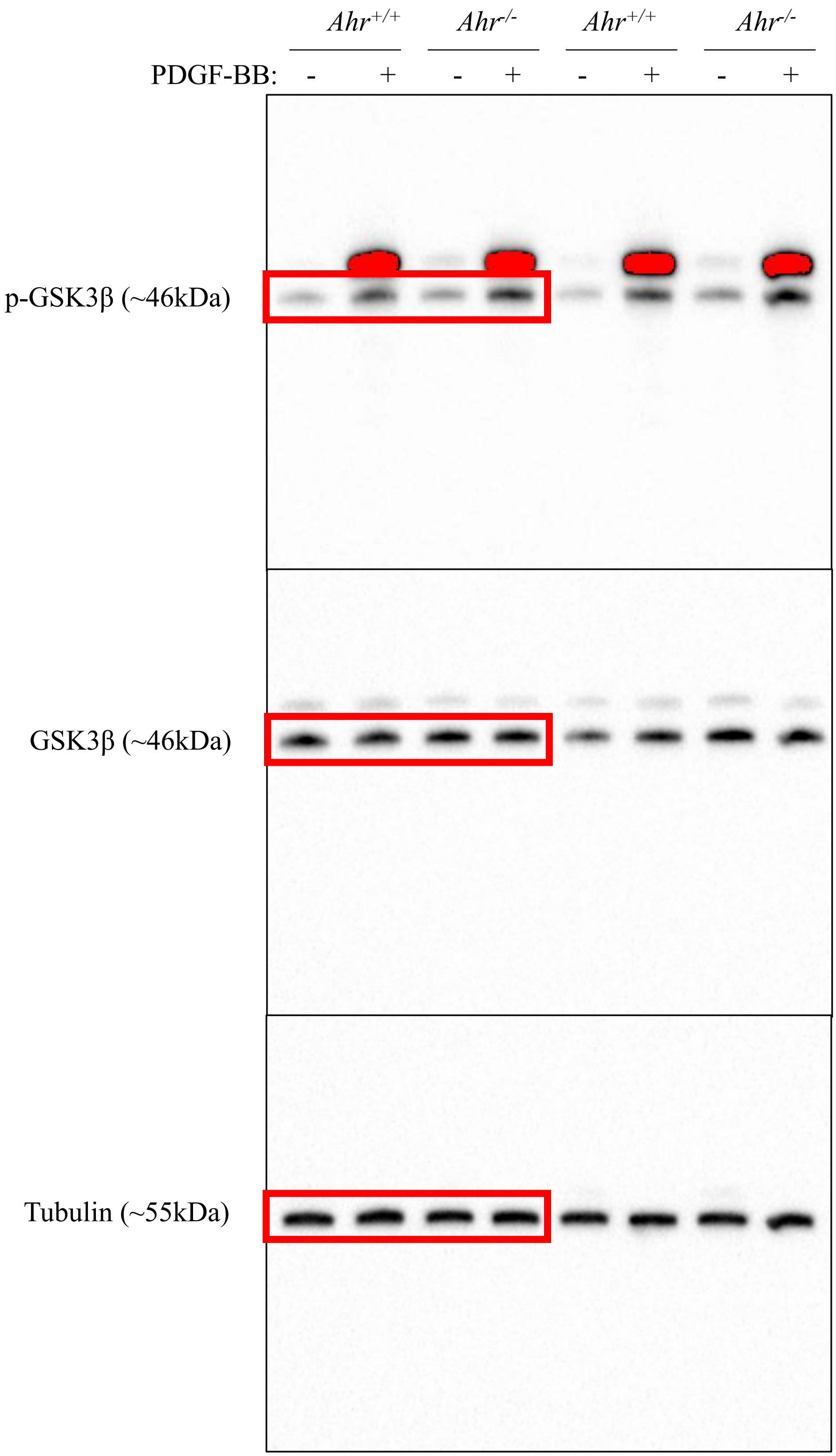

Figure 1D. PDGF-BB (30 min): p-GSK3β (second exposure)

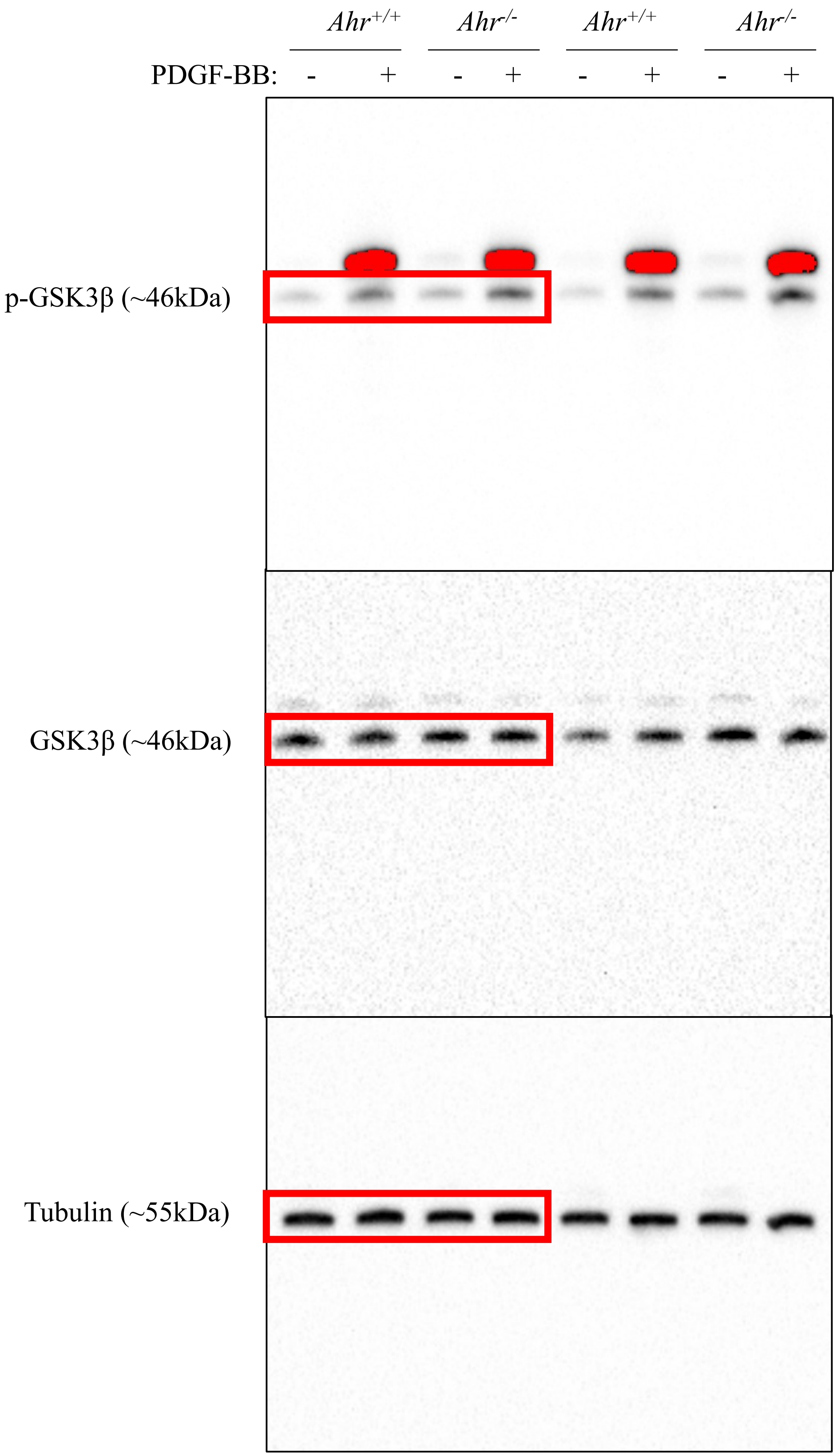

Figure 1D. PDGF-BB (30 min): p-GSK3β (protein ladder)

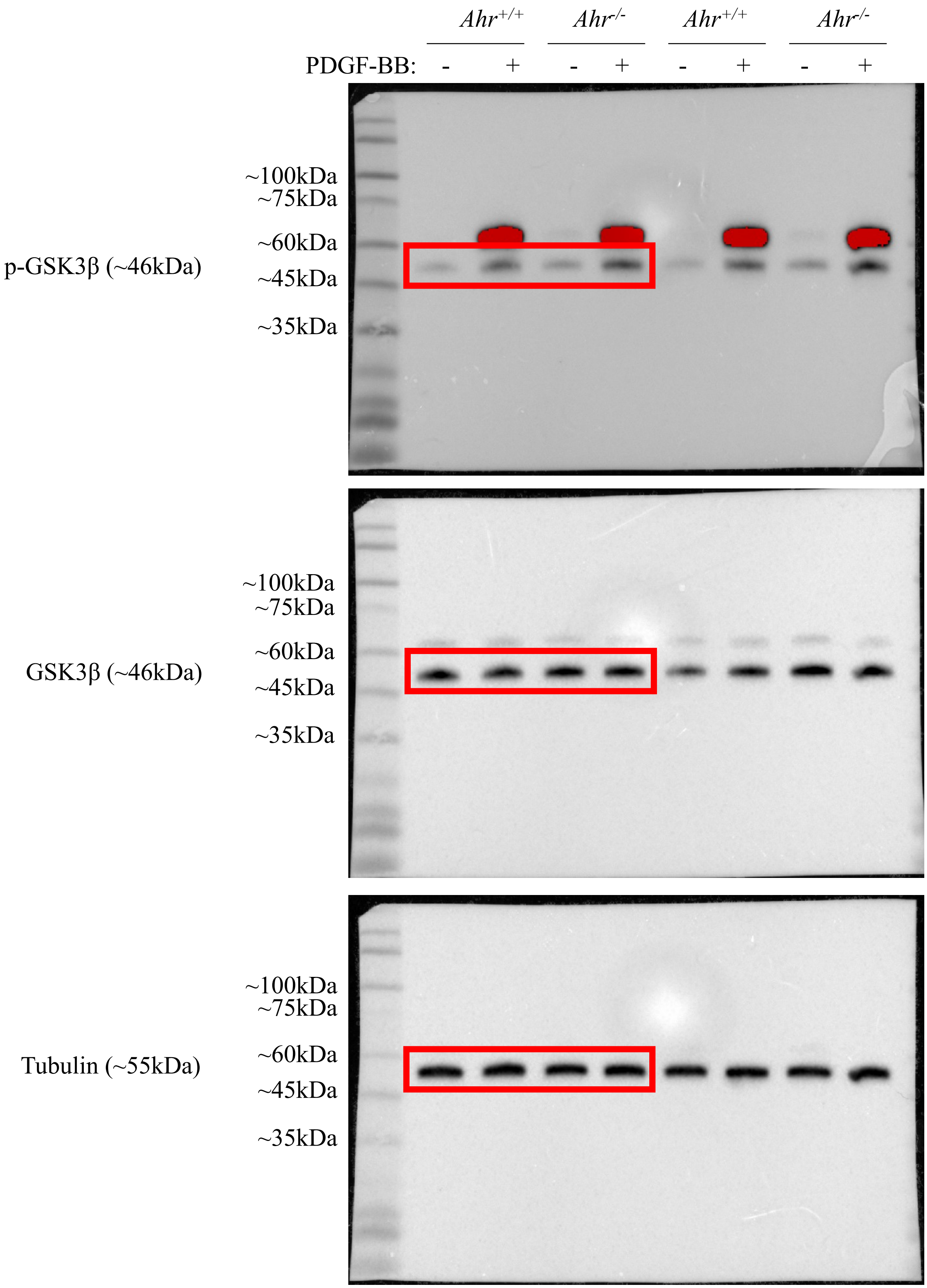

Figure 2A. 5 min CSE: p-Akt (right = second exposure)

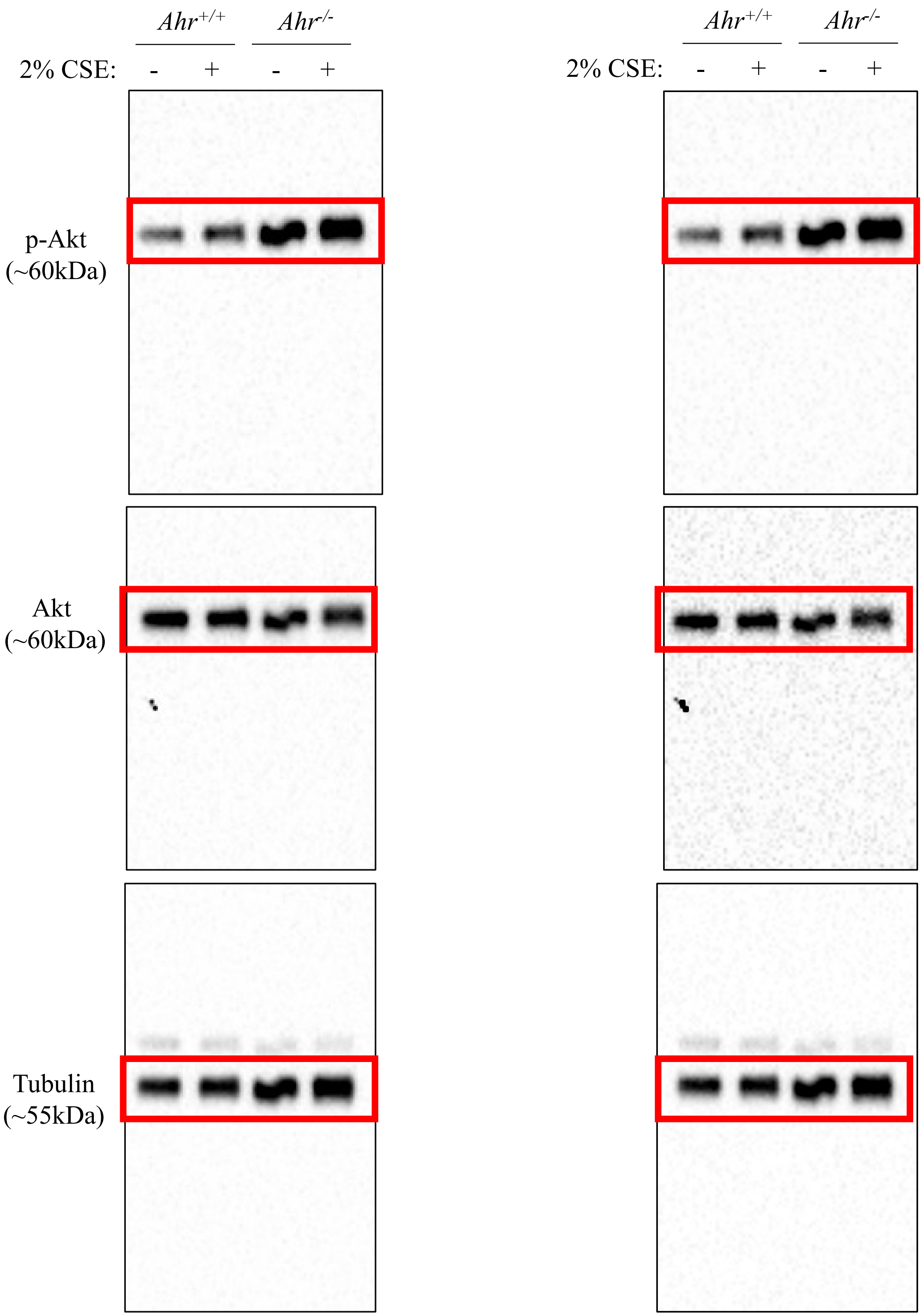

Figure 2A. 5 min CSE: p-Akt (protein ladder)

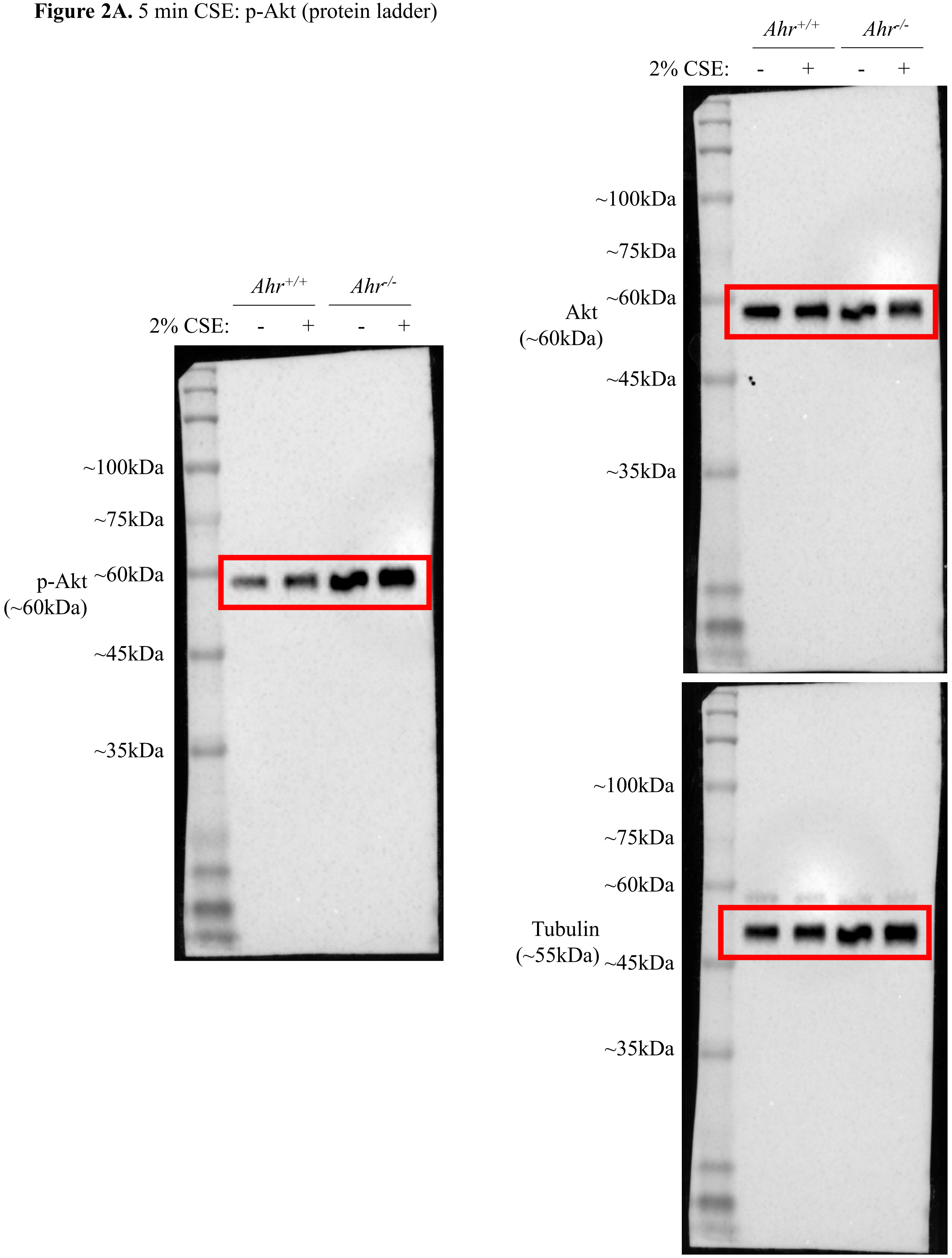

Figure 2B. 5 min CSE: p-GSK3β (right = second exposure)

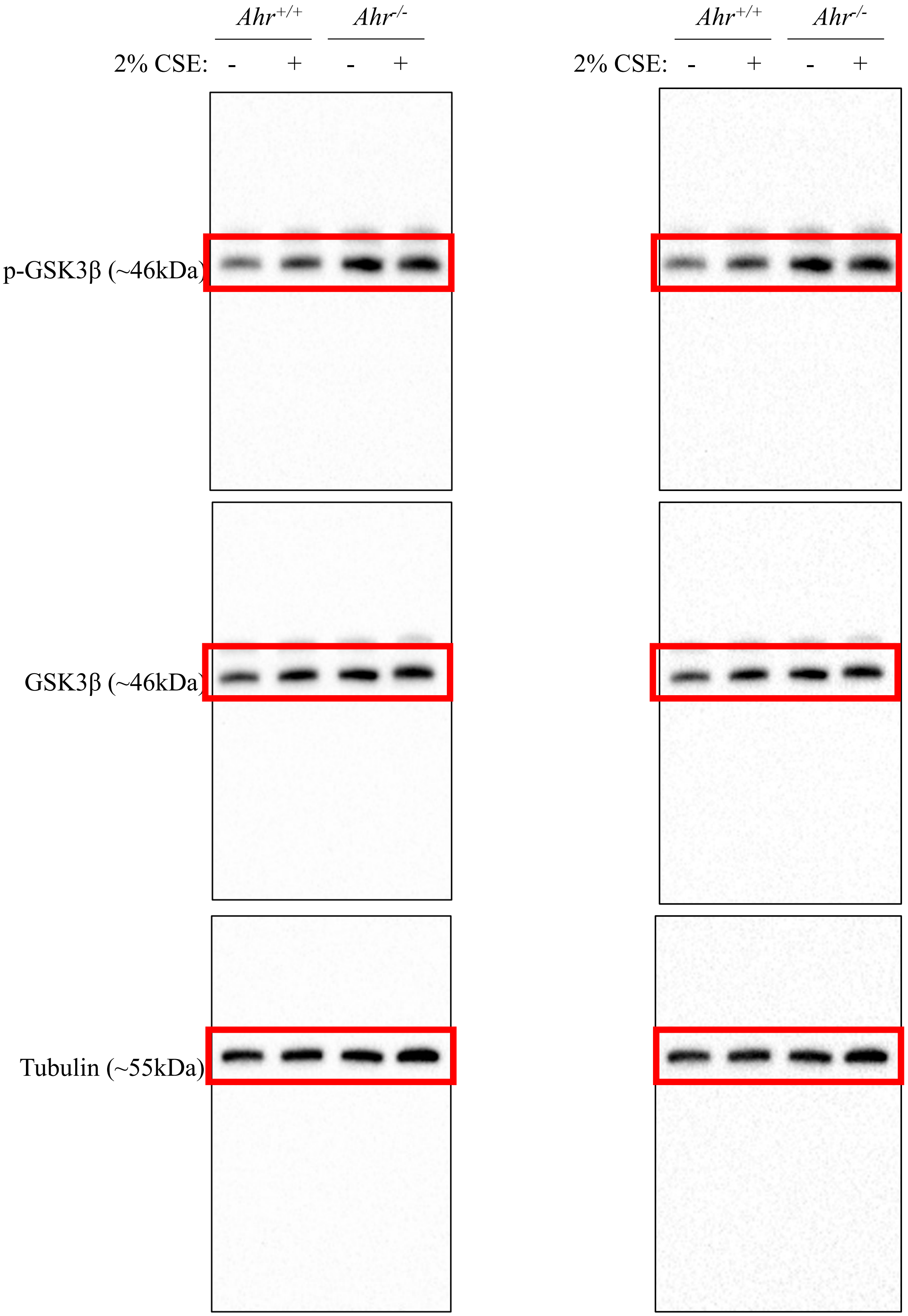

Figure 2B. 5 min CSE: p-GSK3β (protein ladder)

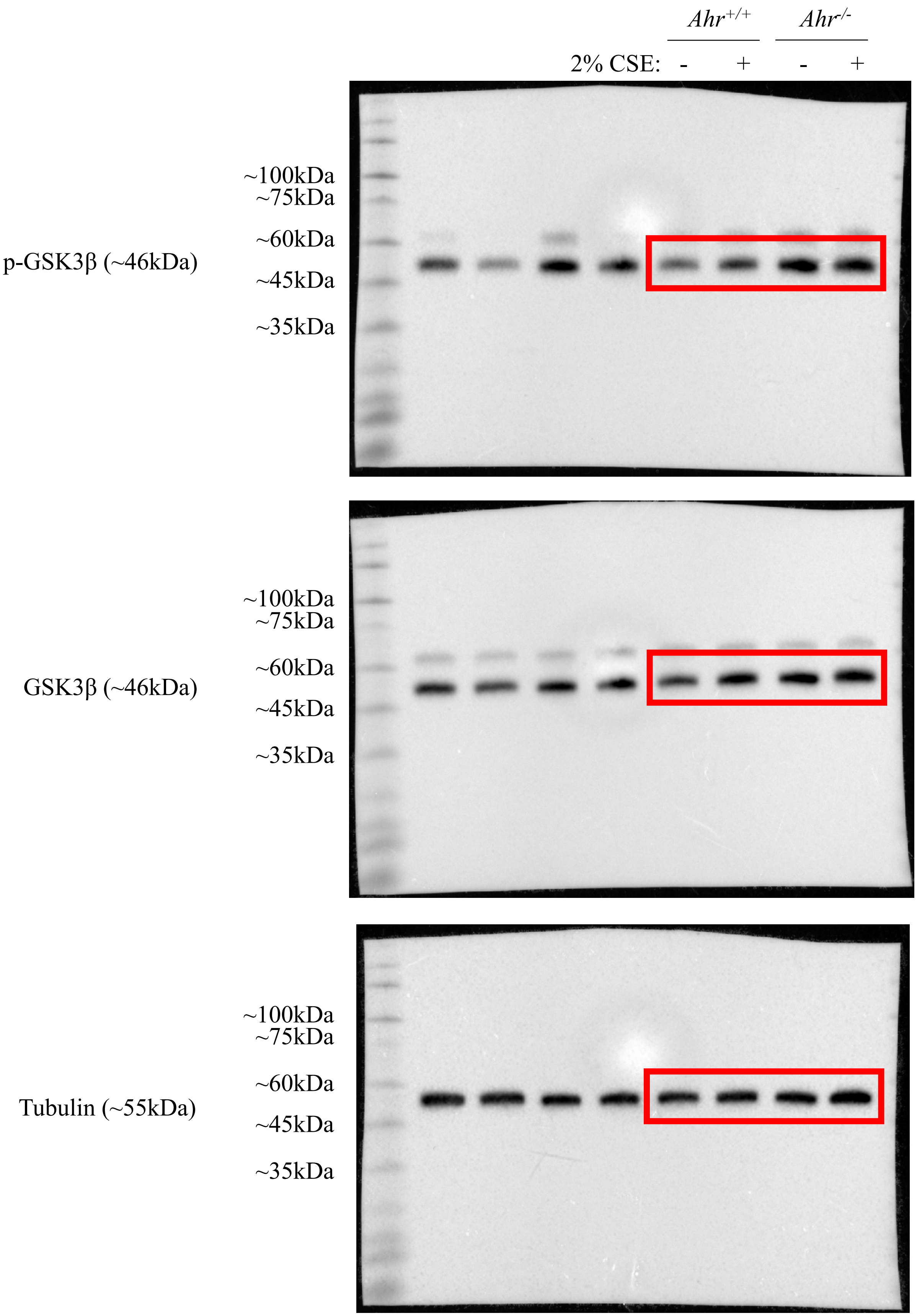

Figure 2C. CSE time course: p-Akt

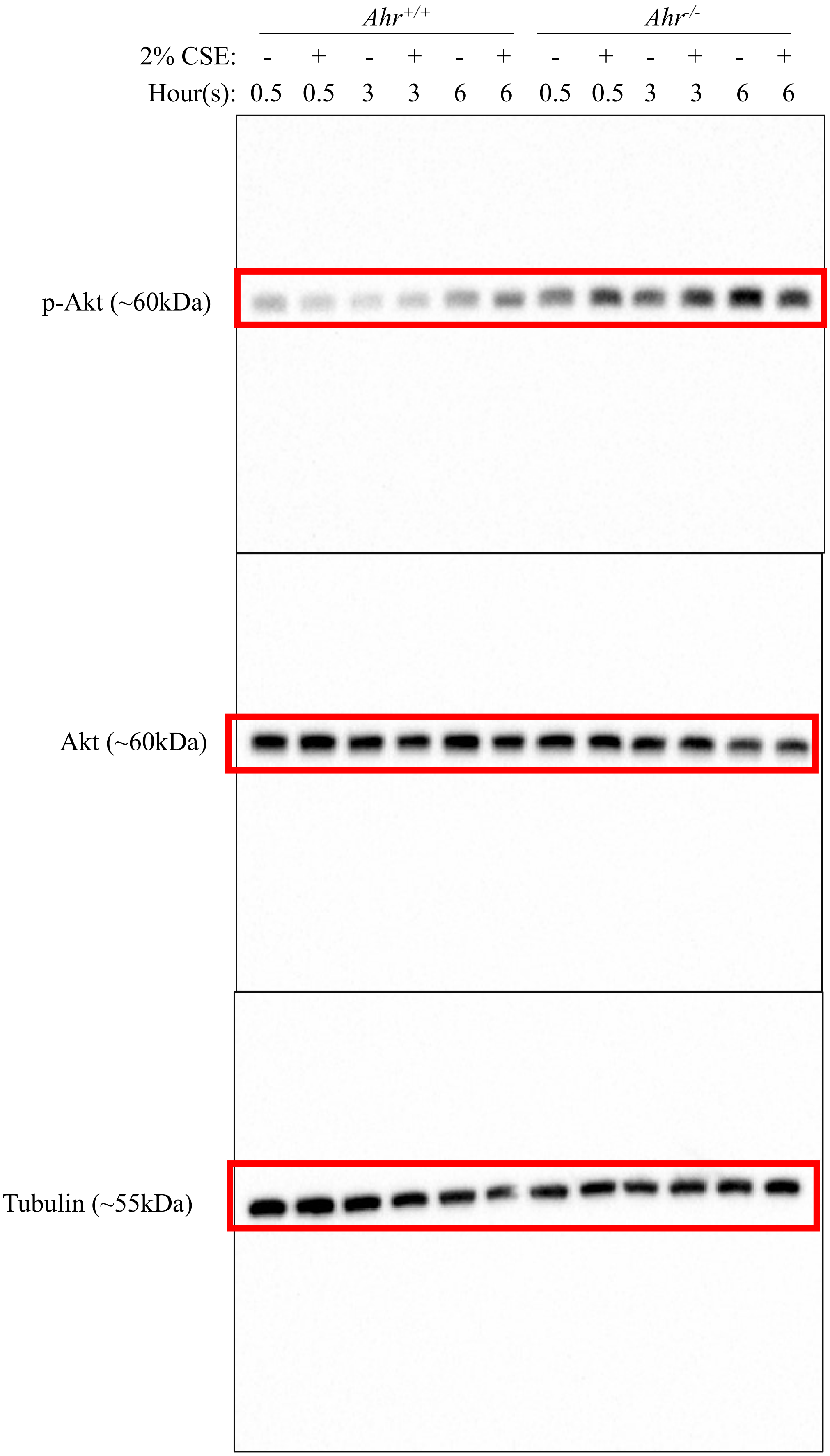

Figure 2C. CSE time course: p-Akt (second exposure)

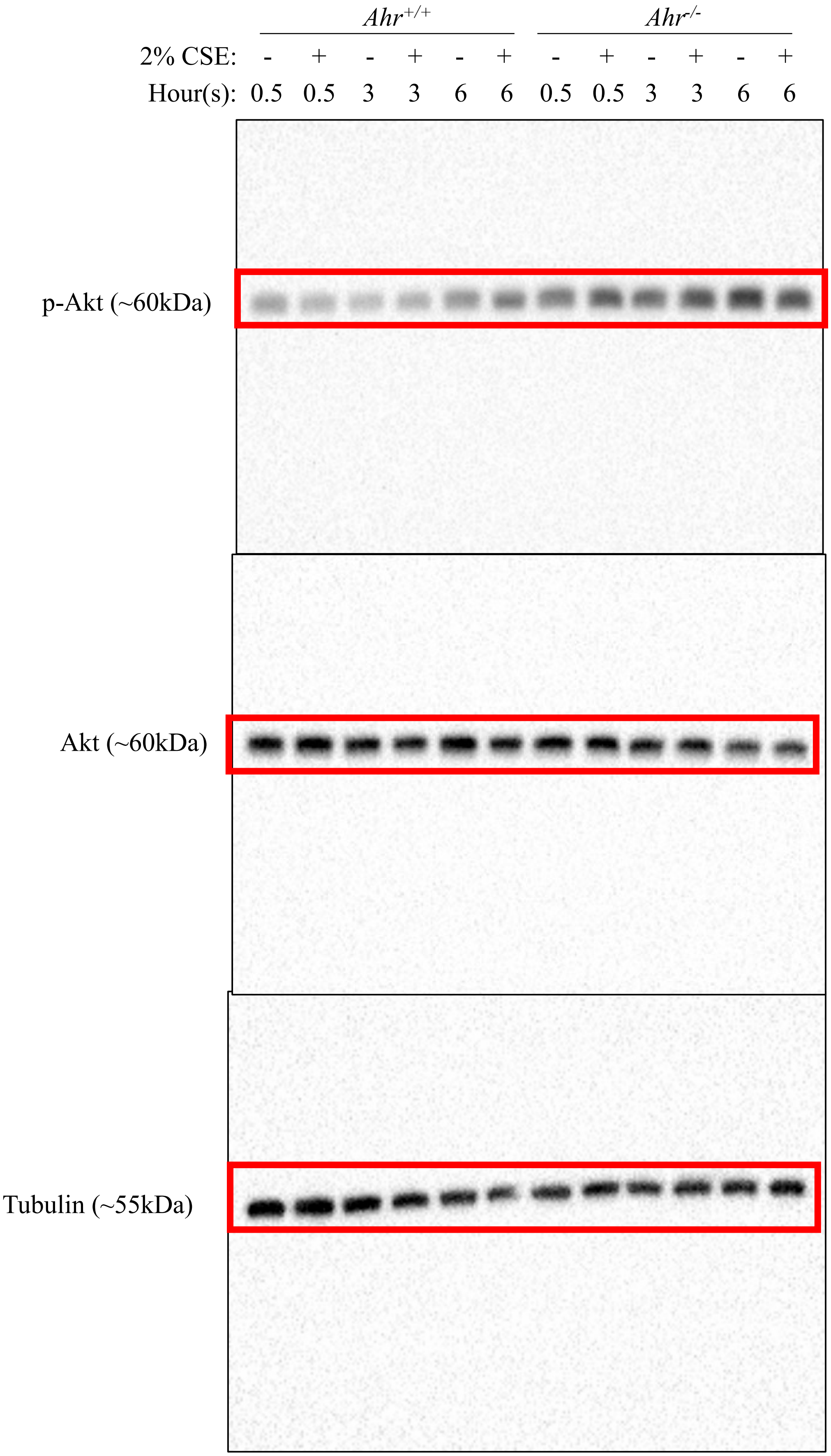

Figure 2C. CSE time course: p-Akt (protein ladder)

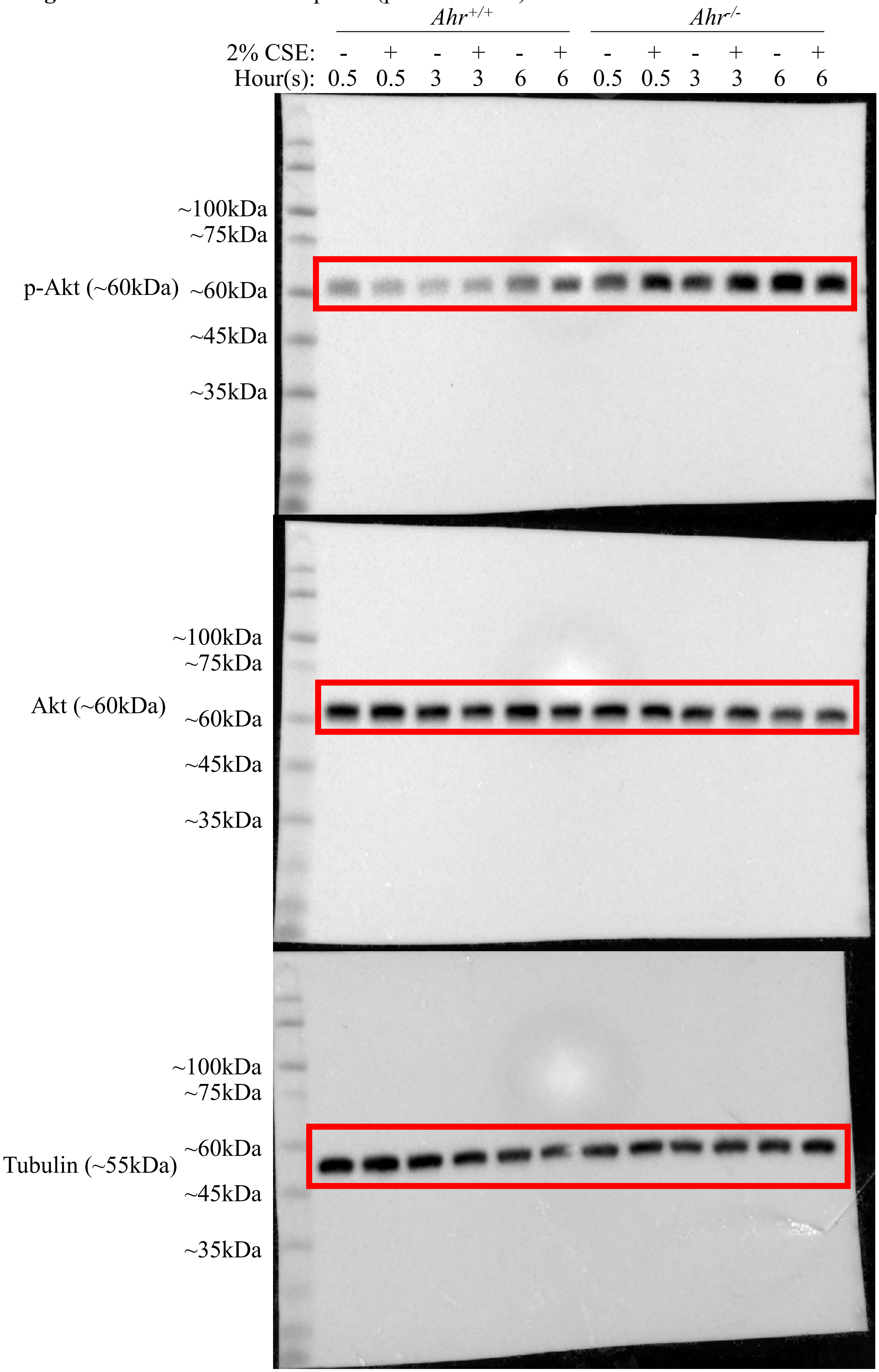

Figure 2D. CSE time course: p-GSK3β

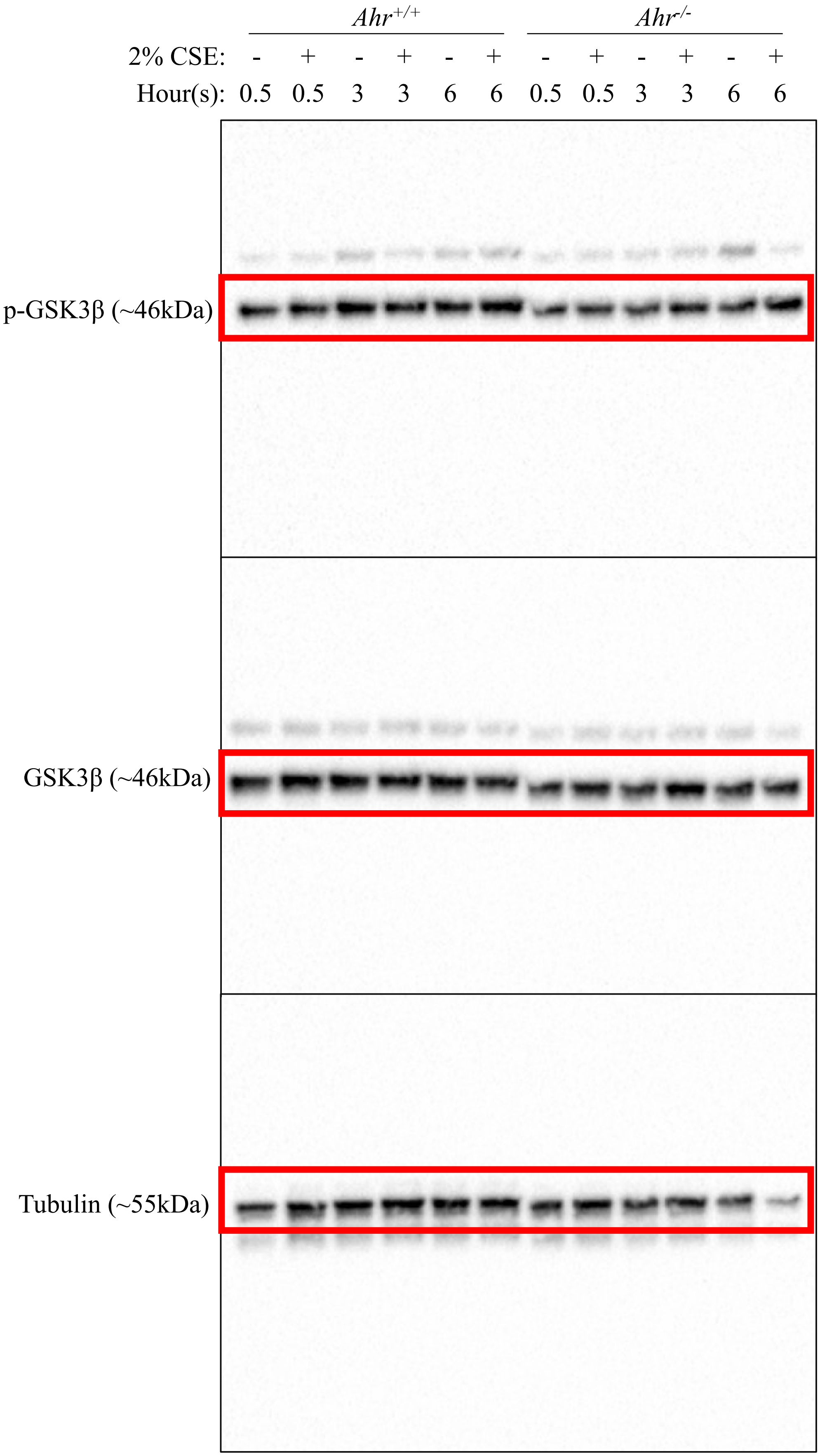

Figure 2D. CSE time course: p-GSK3β (second exposure)

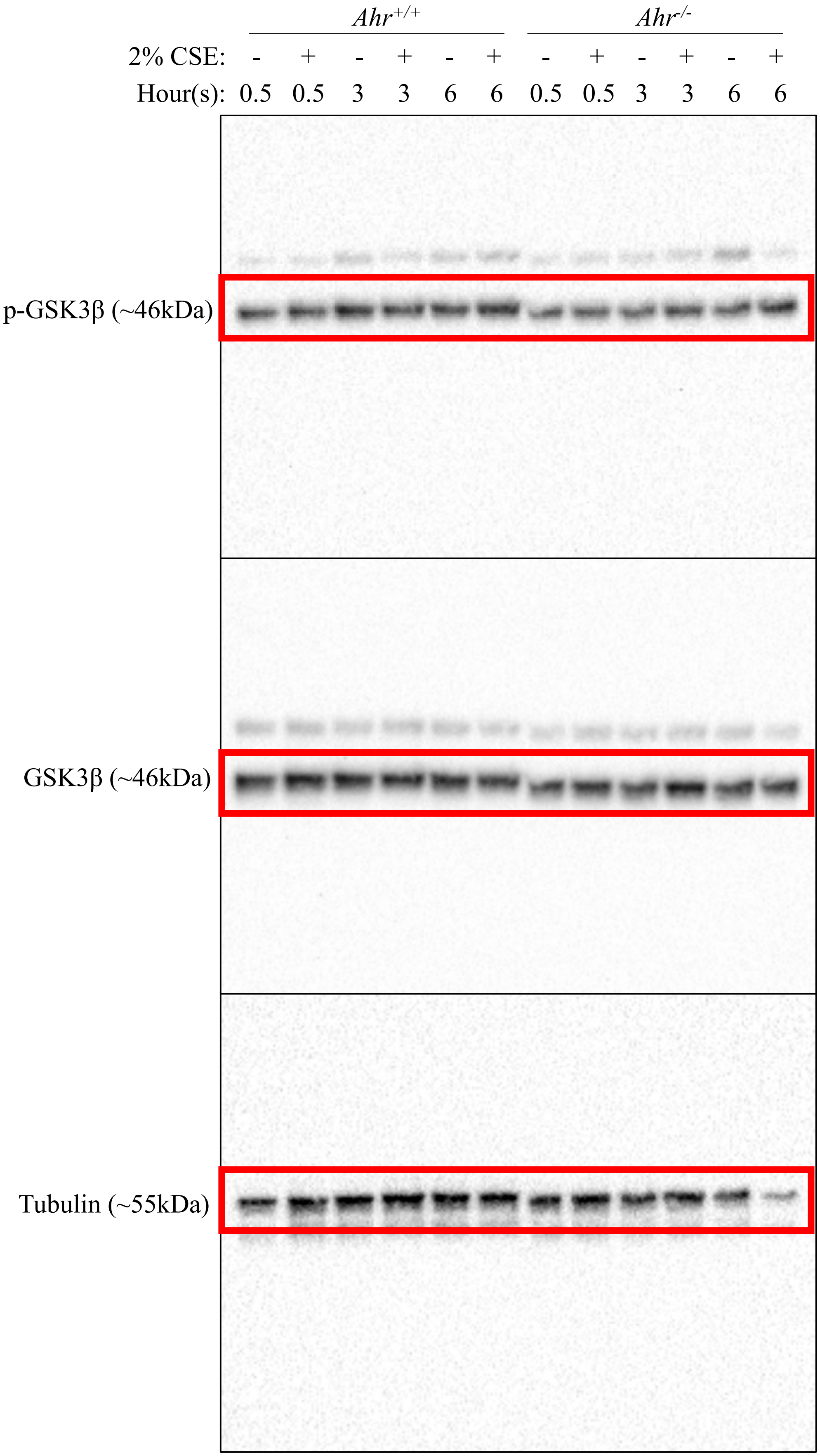

Figure 2D. CSE time course: p-GSK3β (protein ladder)

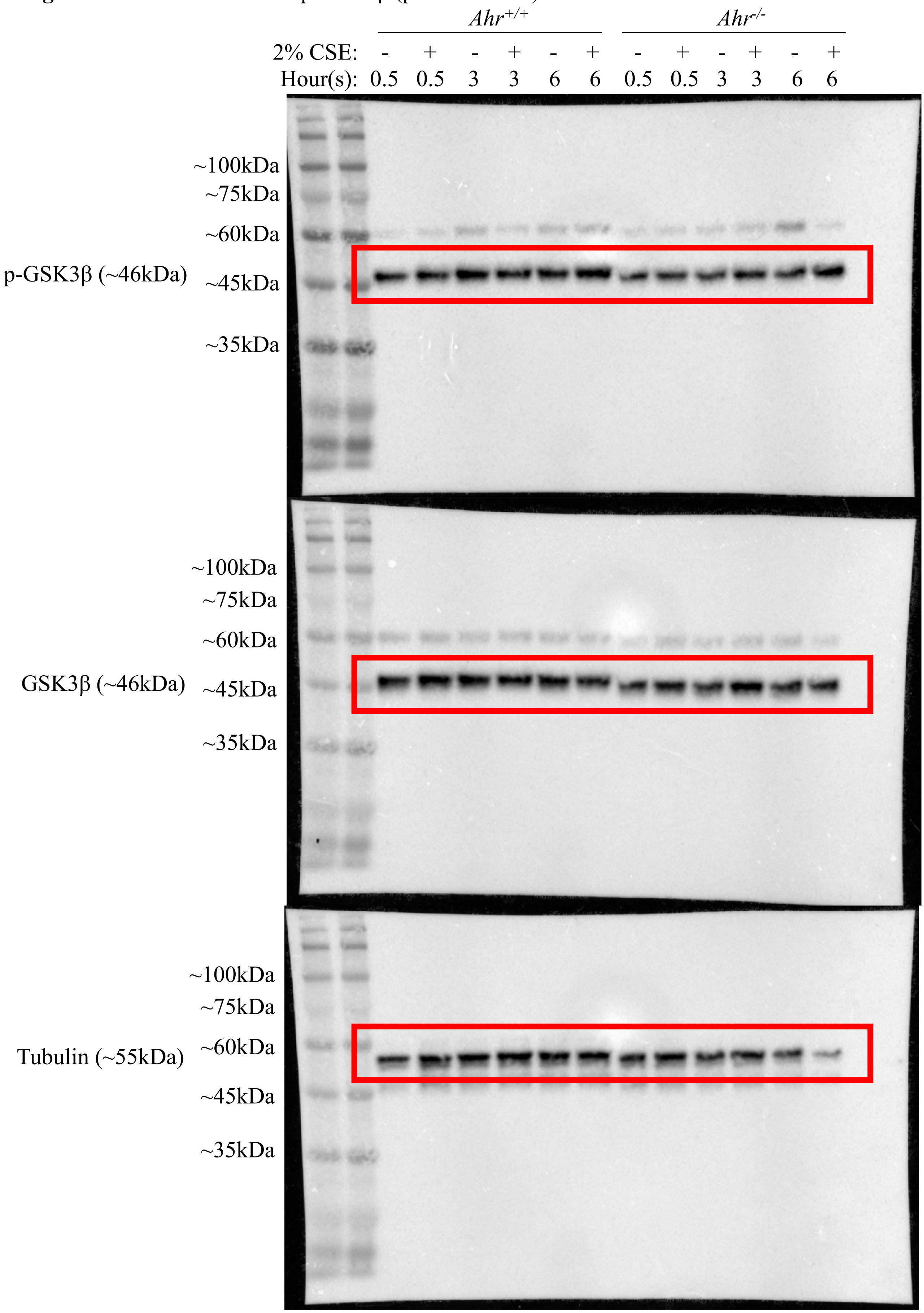

Figure 3A. B[a]P: p-Akt (right = second exposure)

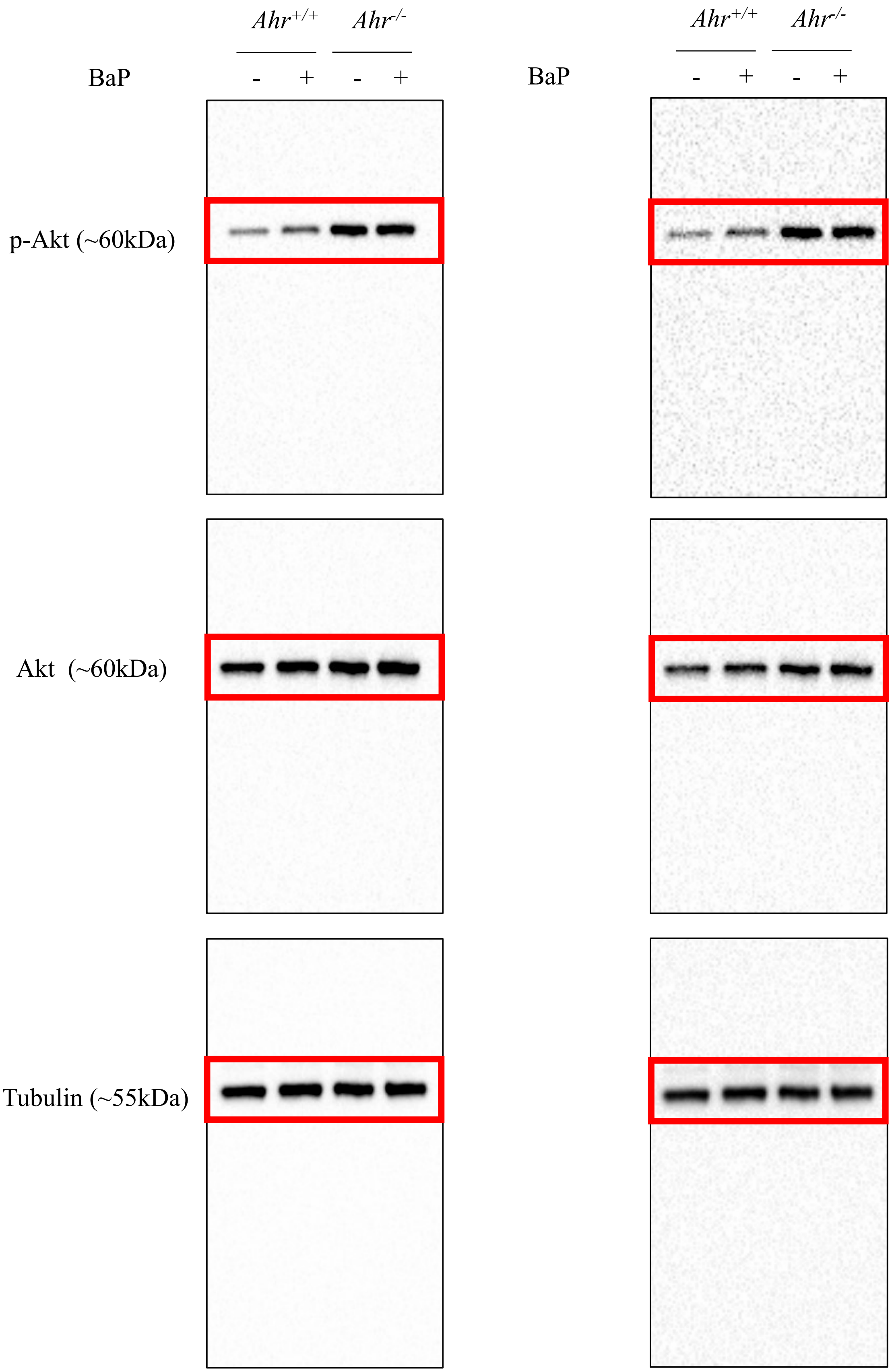

Figure 3A. B[a]P: p-Akt (protein ladder)

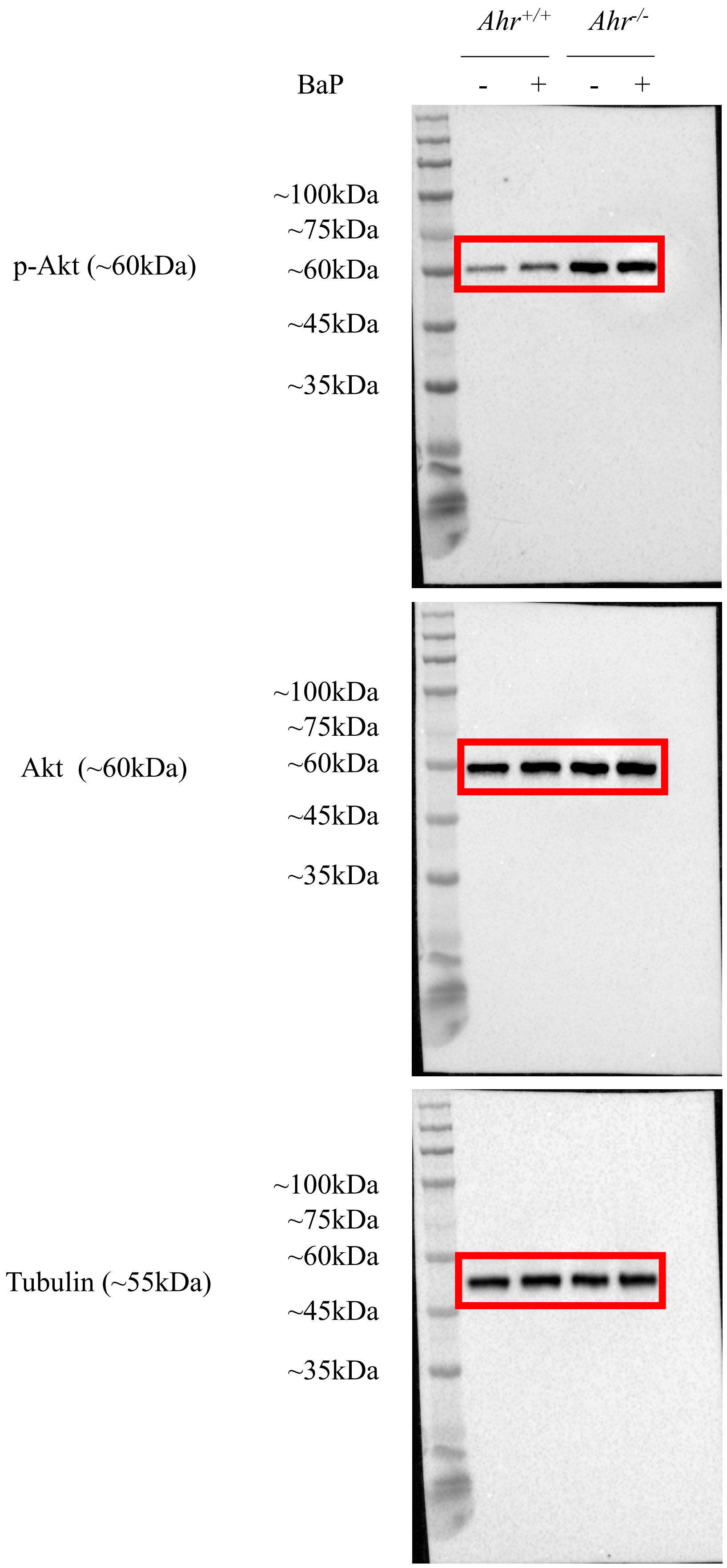

Figure 3B. B[a]P: p-GSK3β (right = second exposure)

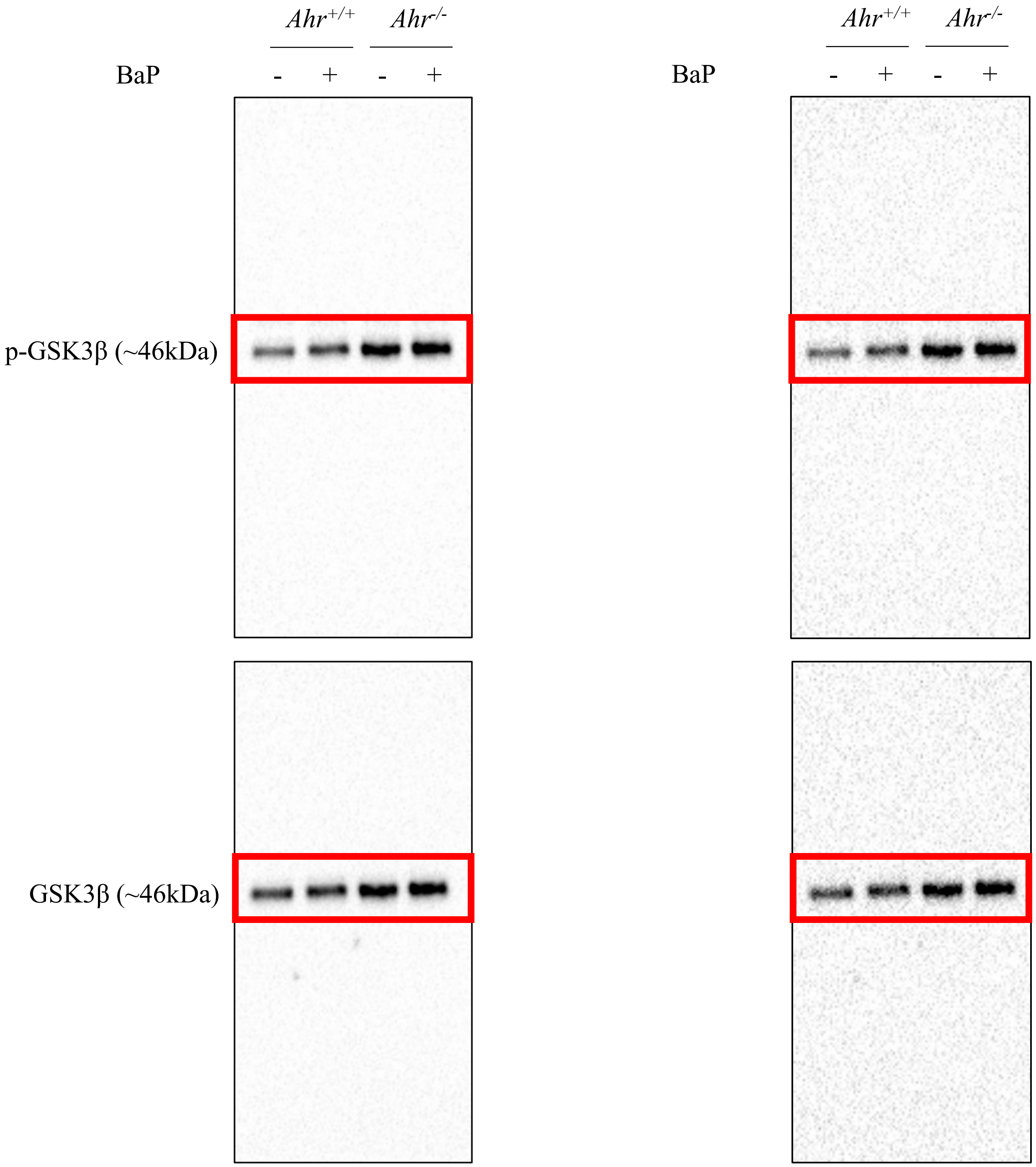

Figure 3B. B[a]P: p-GSK3β (protein ladder)

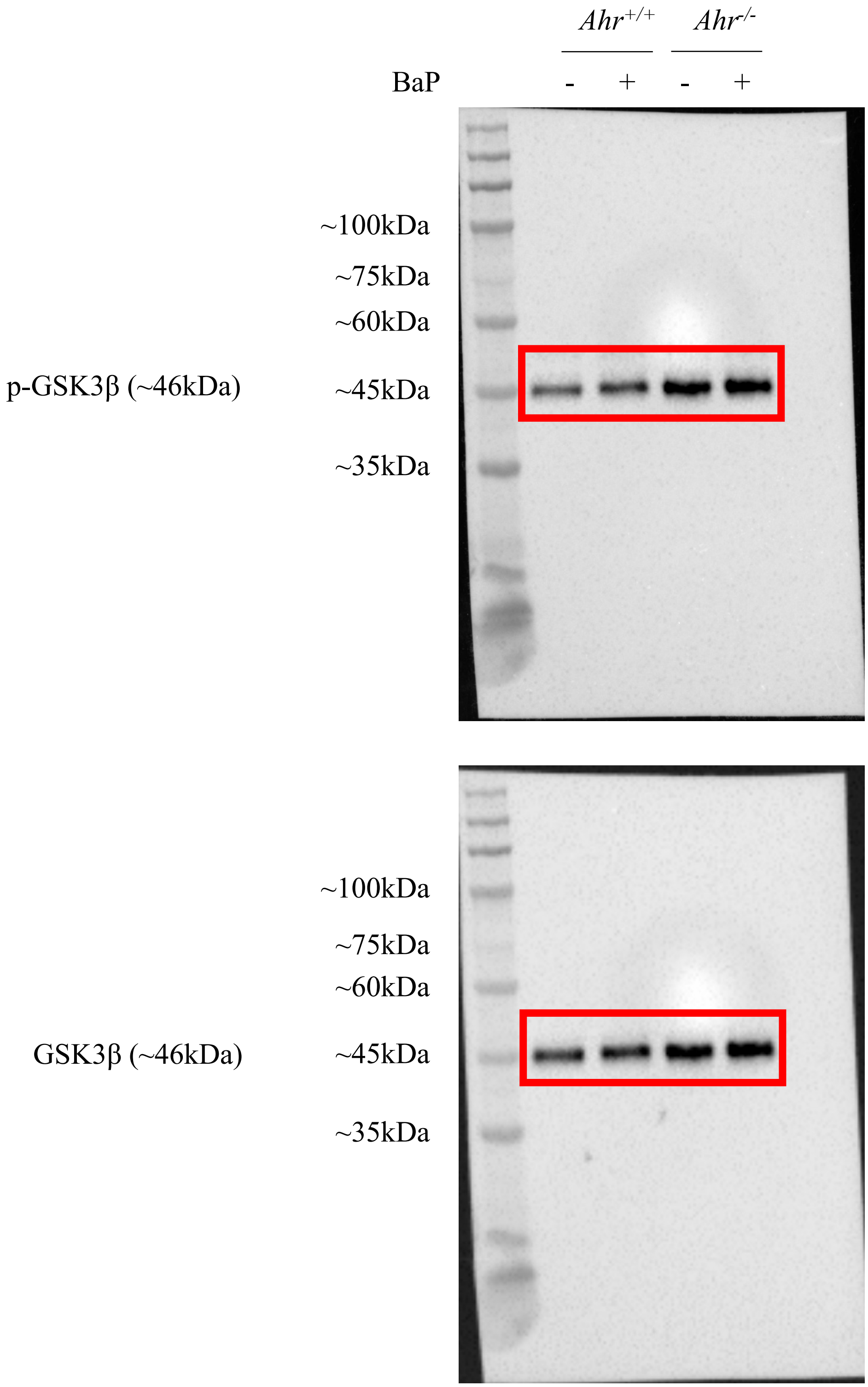

Figure 3C. B[a]P and CH-223191

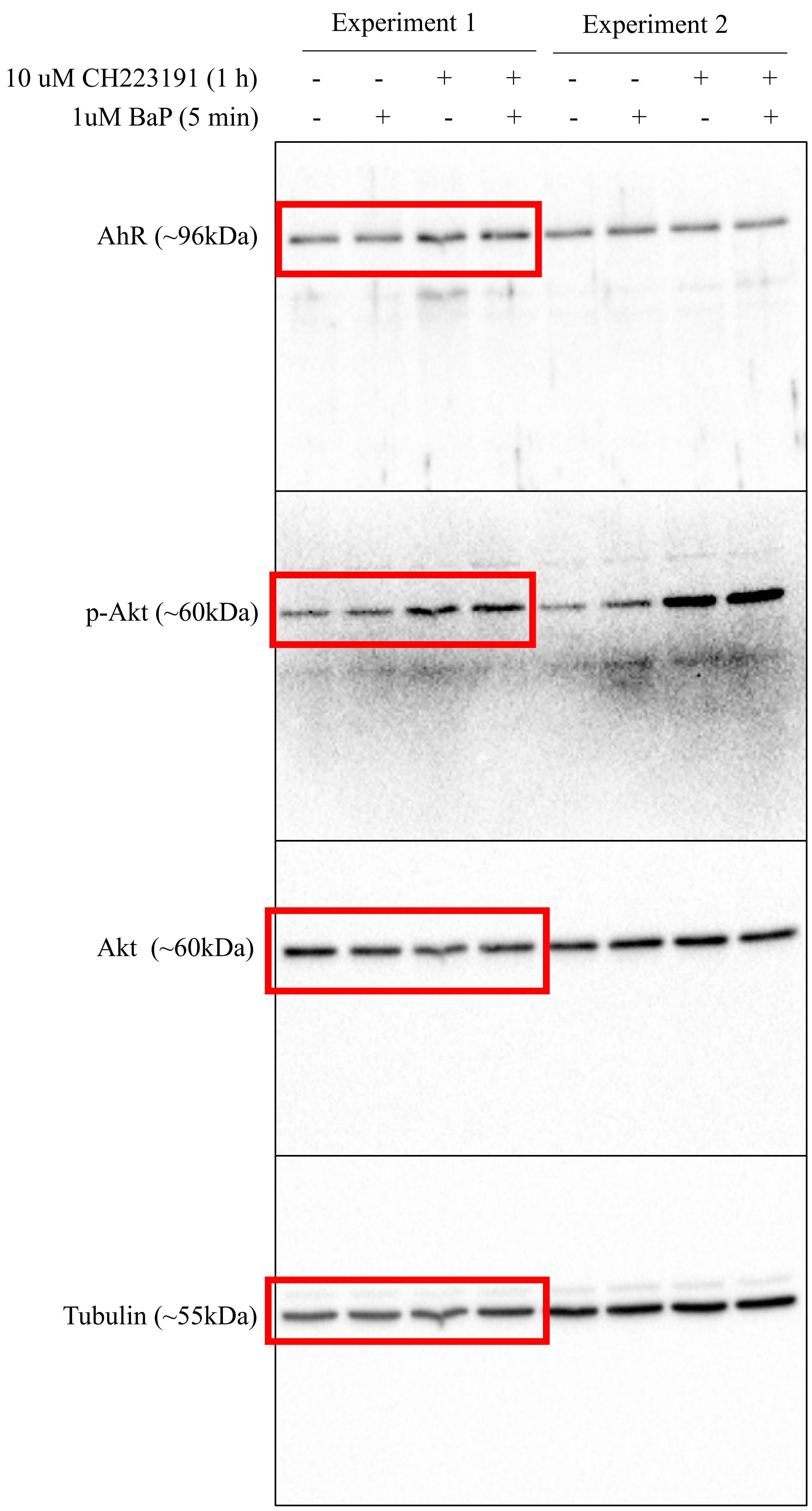

Figure 3C. B[a]P and CH-223191 (second exposure)

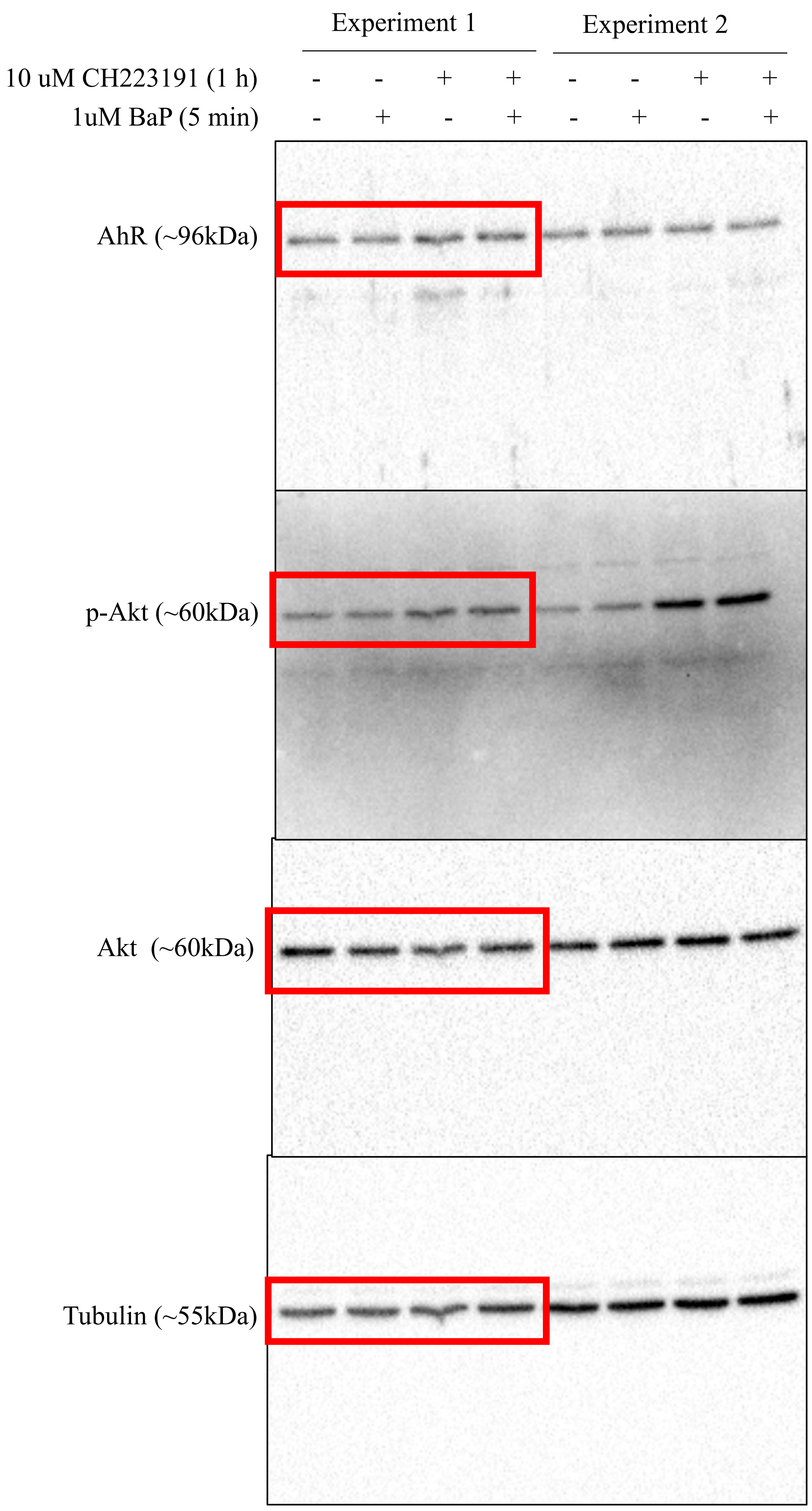

Figure 3C. B[a]P and CH-223191 (protein ladder)

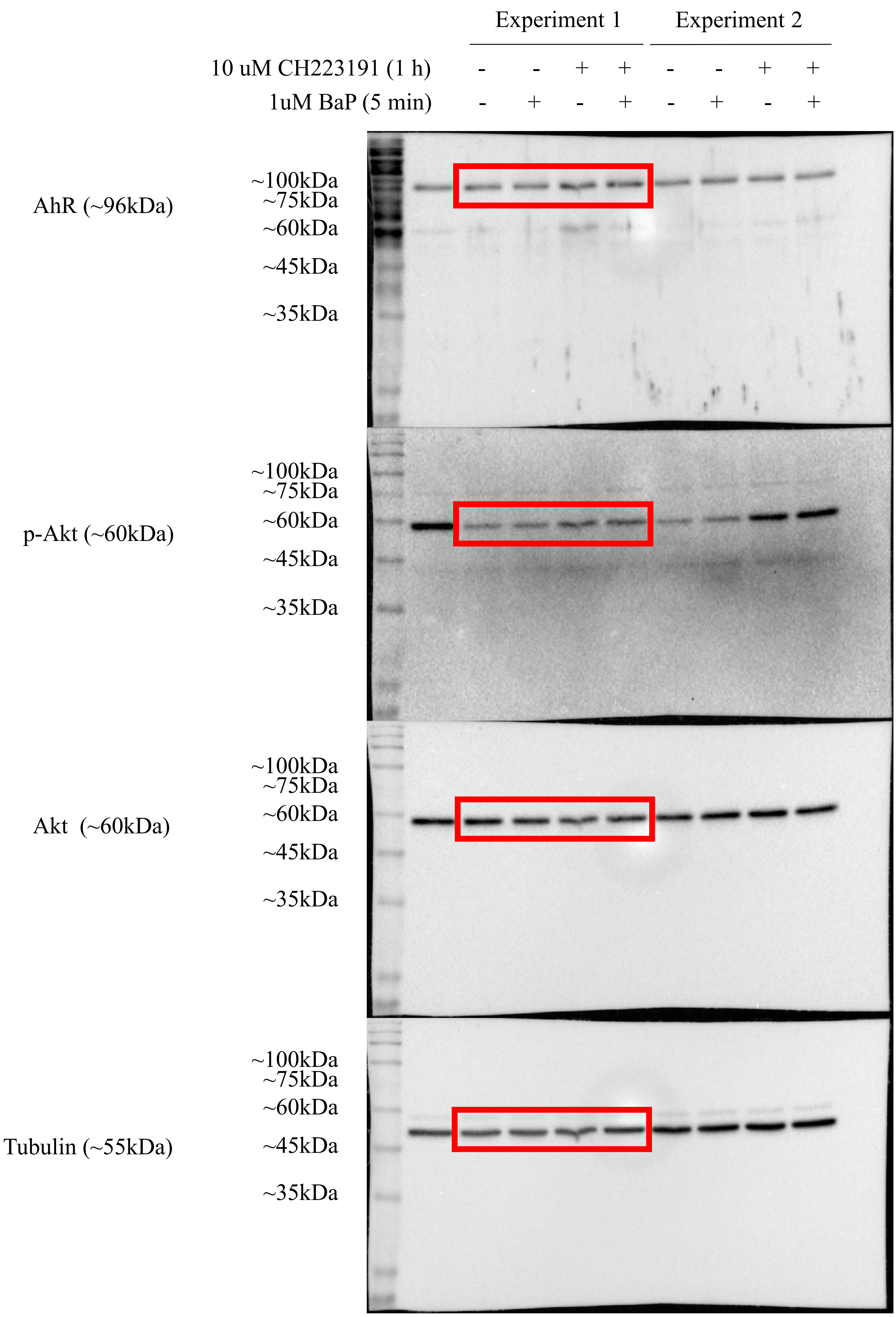

Figure 4.

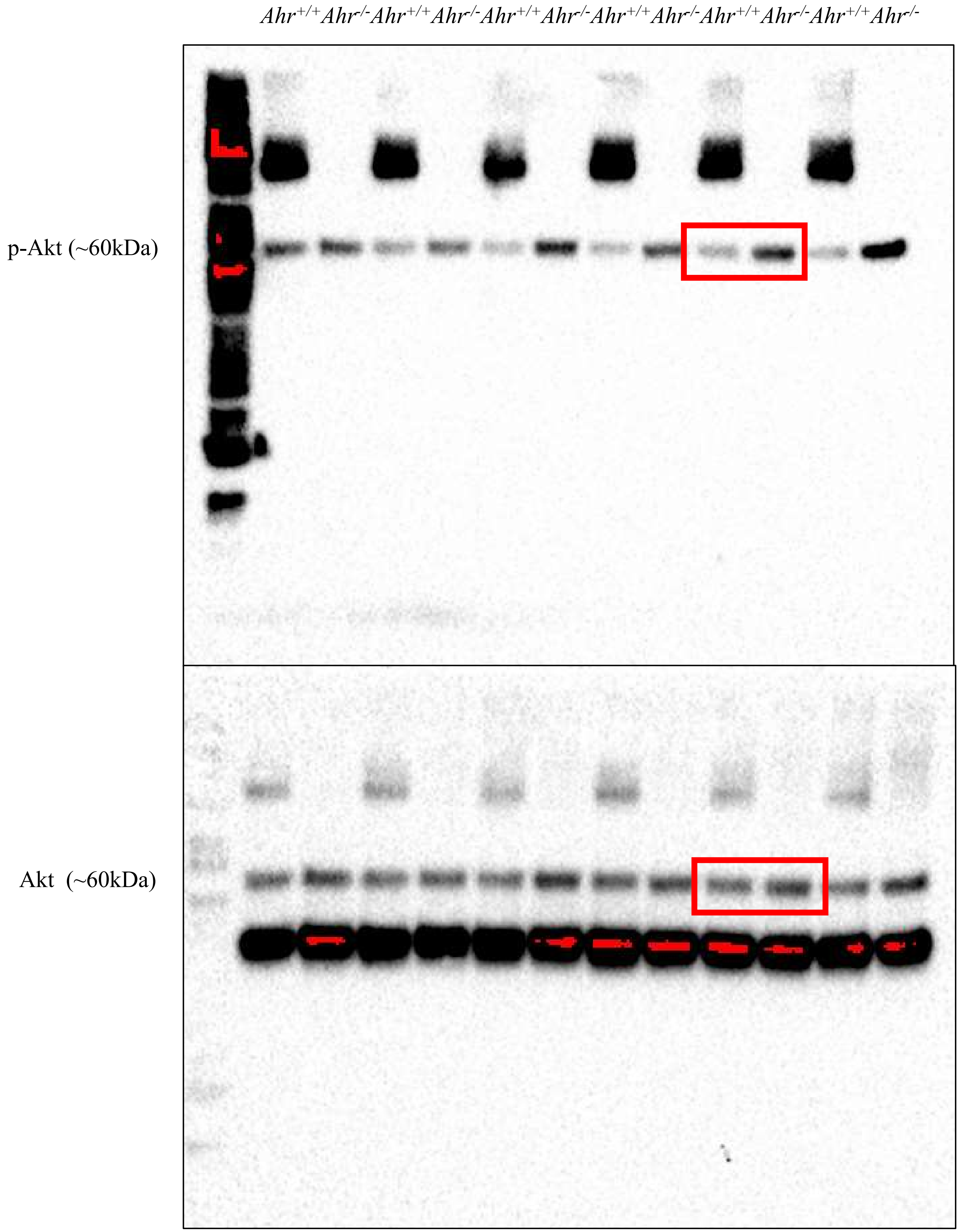
Ahr+/+ Ahr-/- Ahr+/+ Ahr-/- Ahr+/+ Ahr-/- Ahr+/+ Ahr-/- Ahr+/+ Ahr-/- Ahr+/+ Ahr-/-
p-Akt (~60kDa)
Akt (~60kDa)

Figure 4. (second exposure)

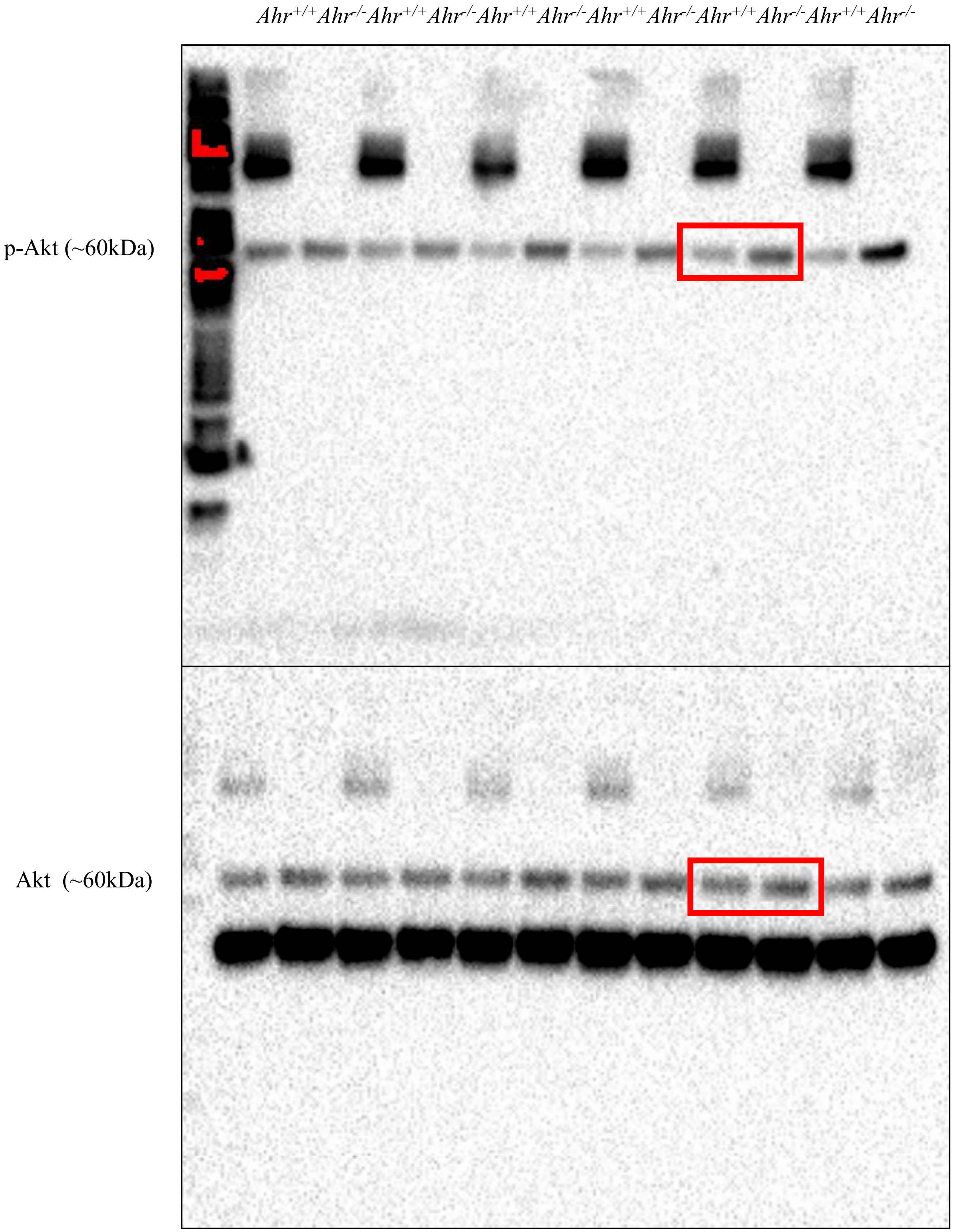

Figure 4. (protein ladder)

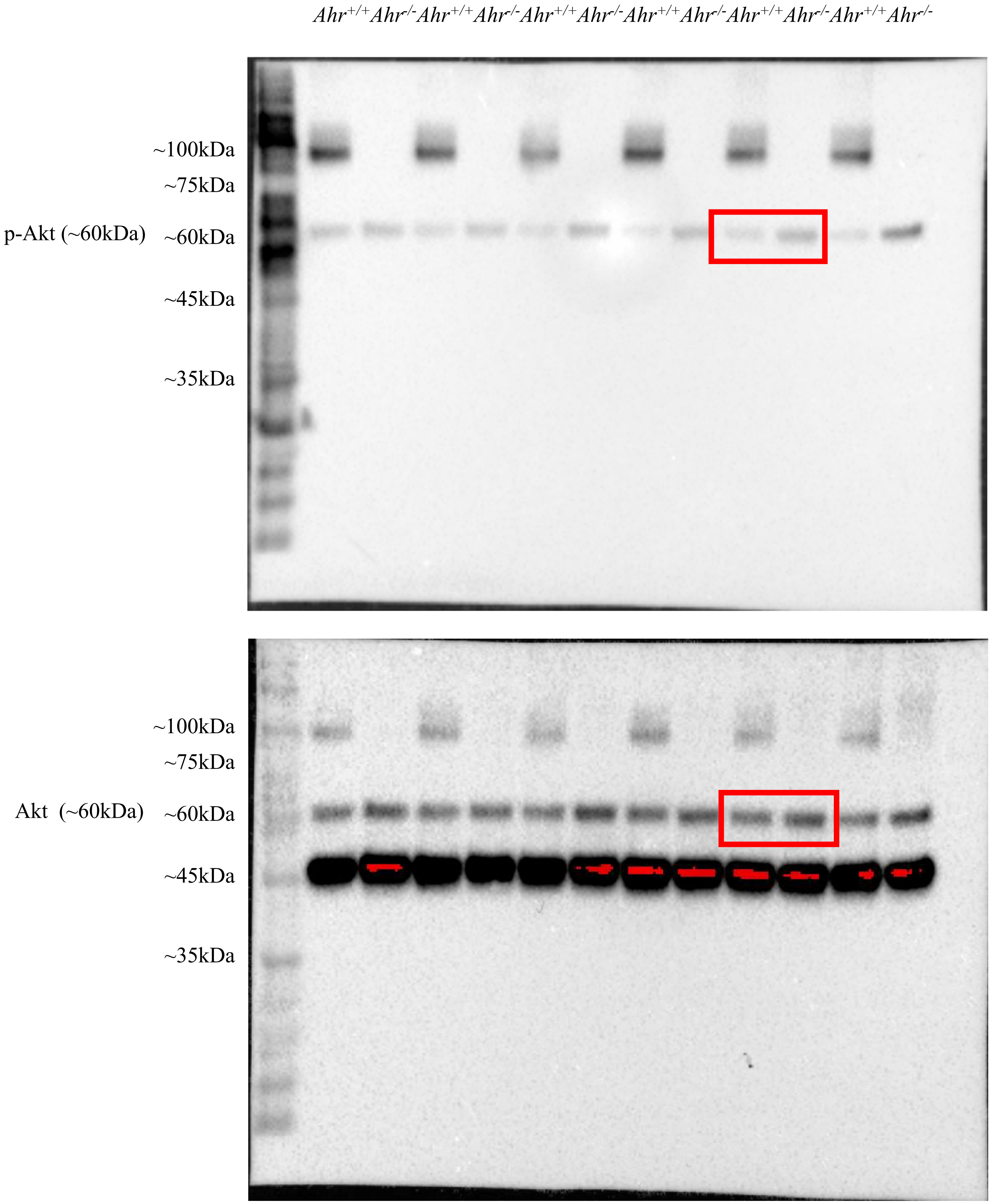

Figure 4.

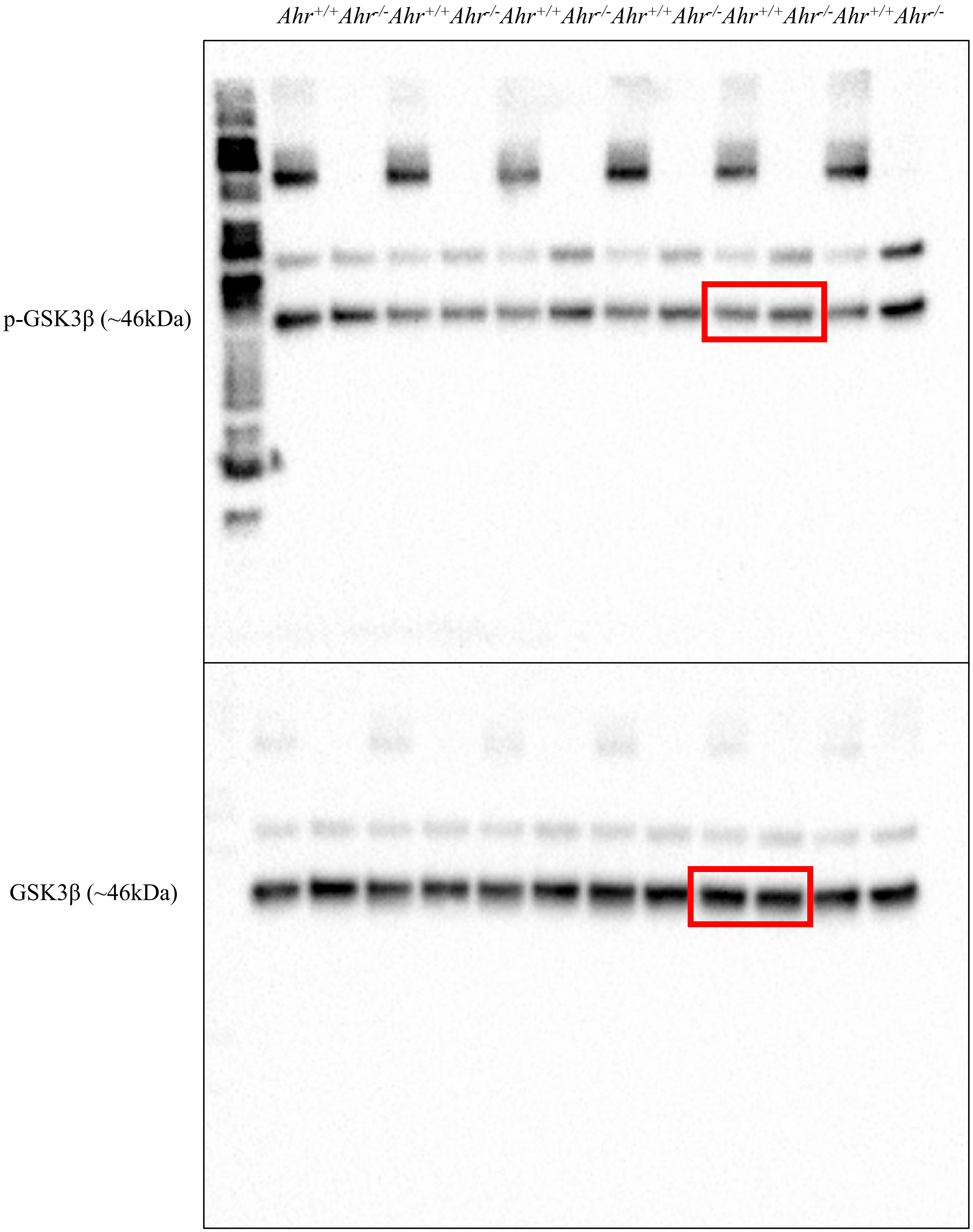

Ahr+/+ Ahr-/- Ahr+/+ Ahr-/- Ahr+/+ Ahr-/- Ahr+/+ Ahr-/- Ahr+/+ Ahr-/- Ahr+/+ Ahr-/-
p-GSK3β (~46kDa)
GSK3β (~46kDa)

Figure 4. (Second exposure)

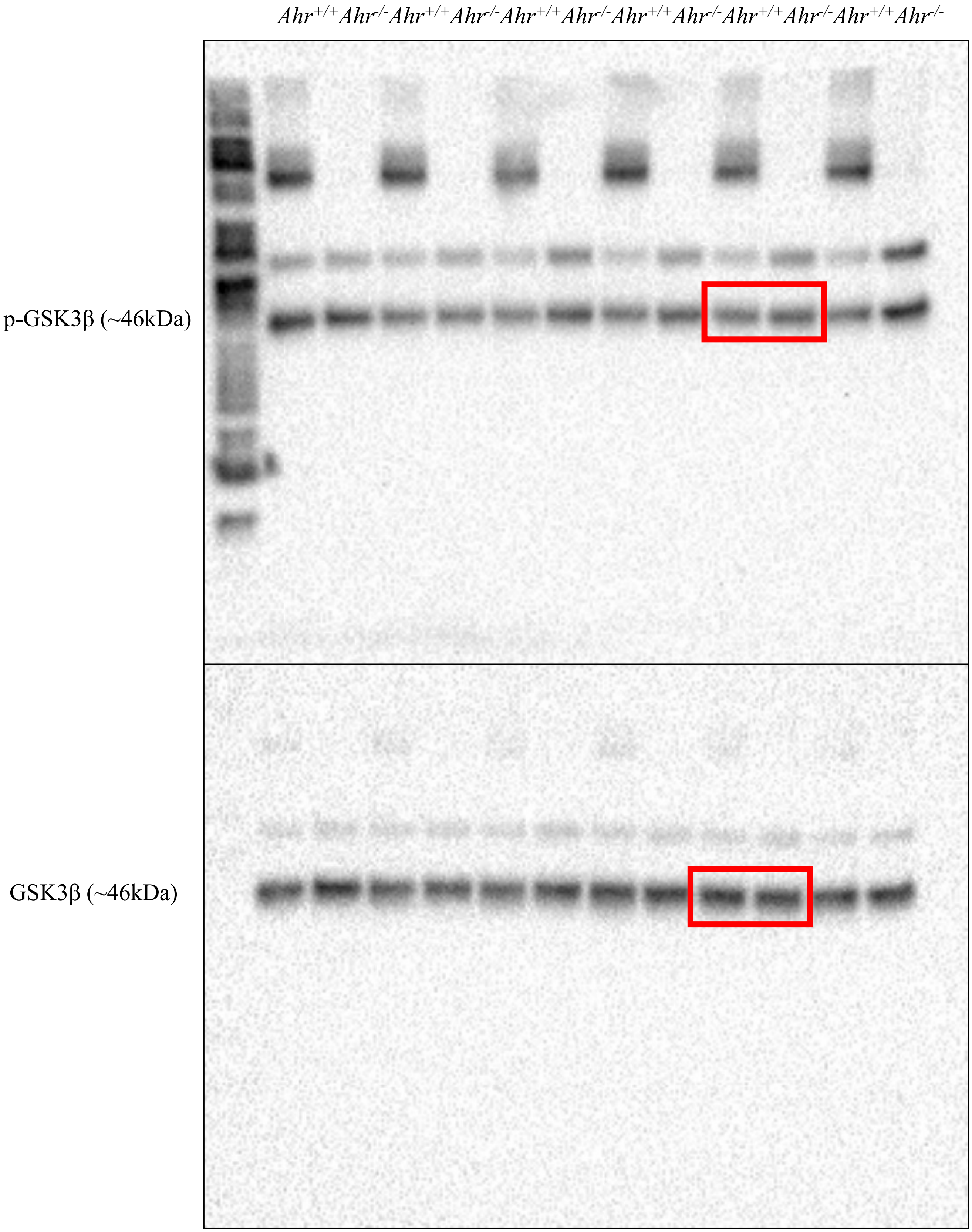

Figure 4.(protein ladder)

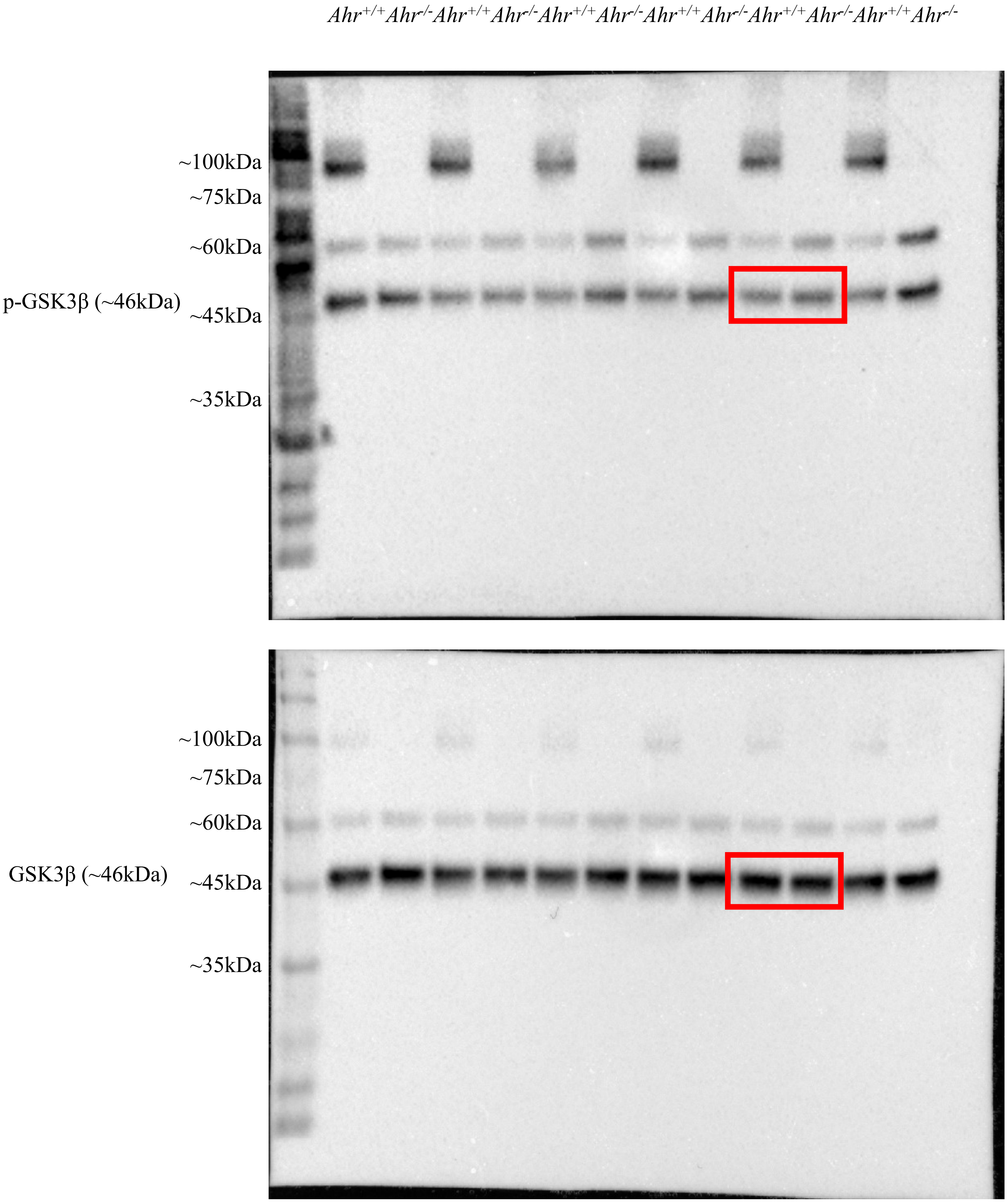

Figure 4.

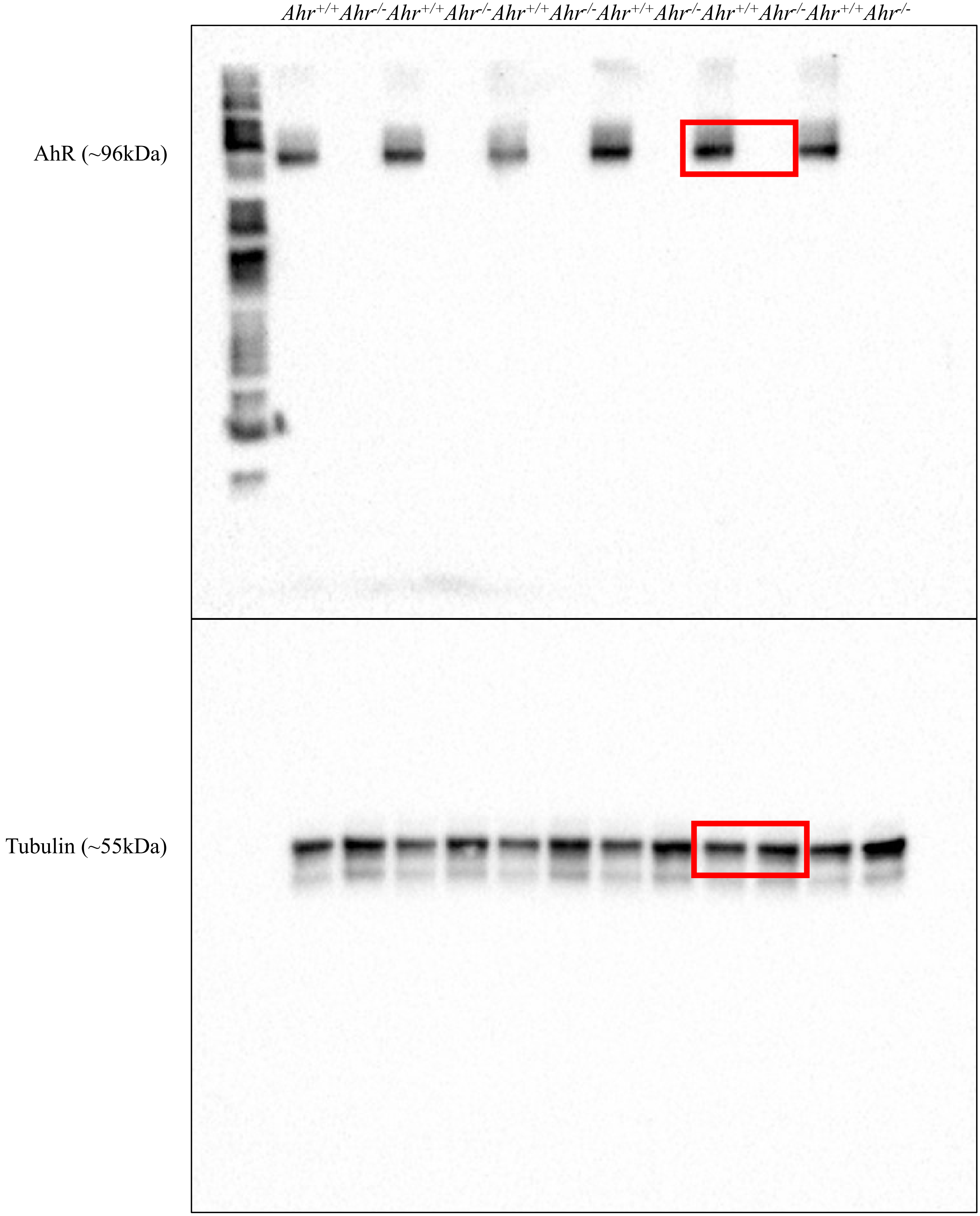
Ahr+/+ Ahr-/- Ahr+/+ Ahr-/- Ahr+/+ Ahr-/- Ahr+/+ Ahr-/- Ahr+/+ Ahr-/- Ahr+/+ Ahr-/-
AhR (~96kDa)
Tubulin (~55kDa)

Figure 4. (Second exposure)

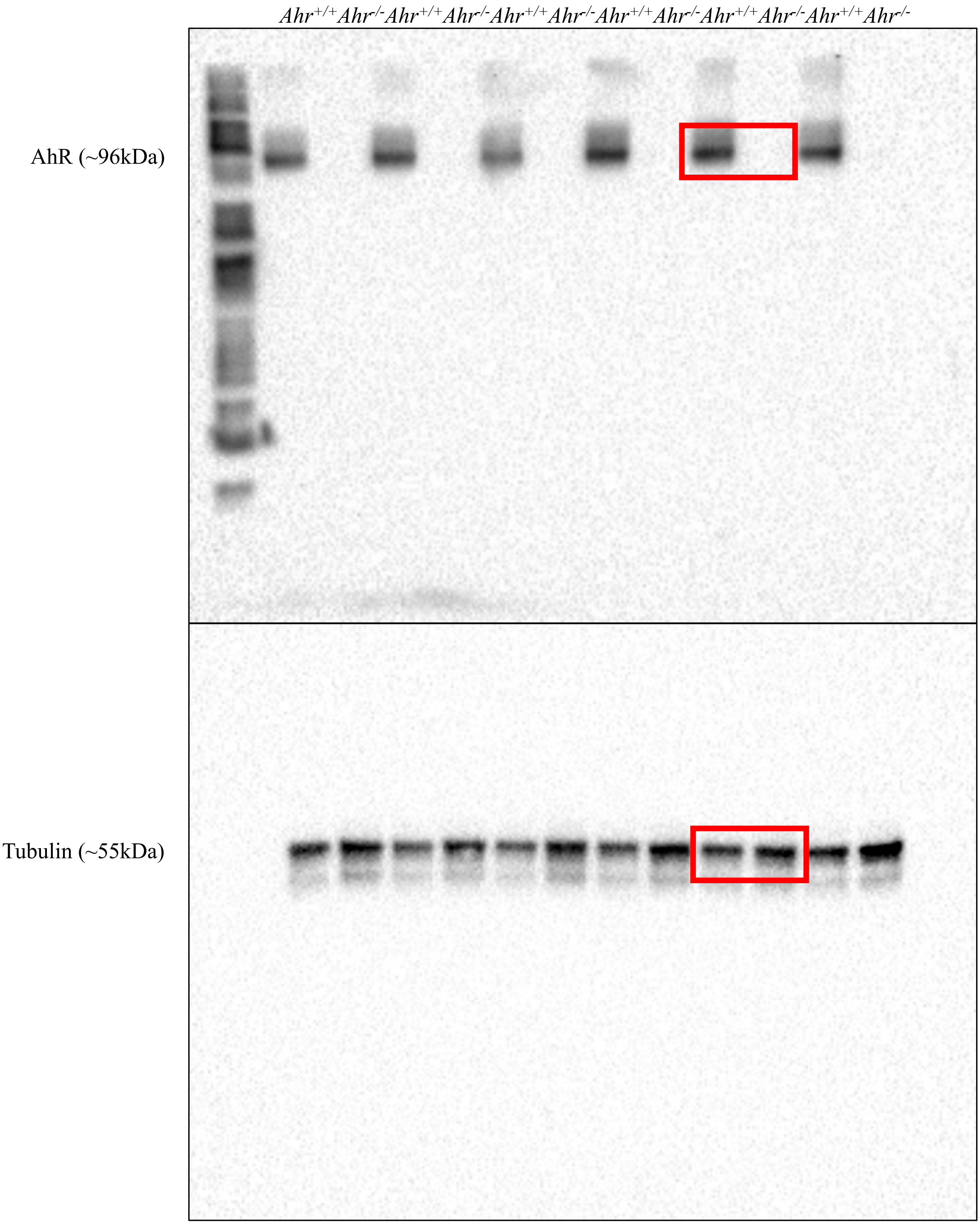

Figure 4. (protein ladder)

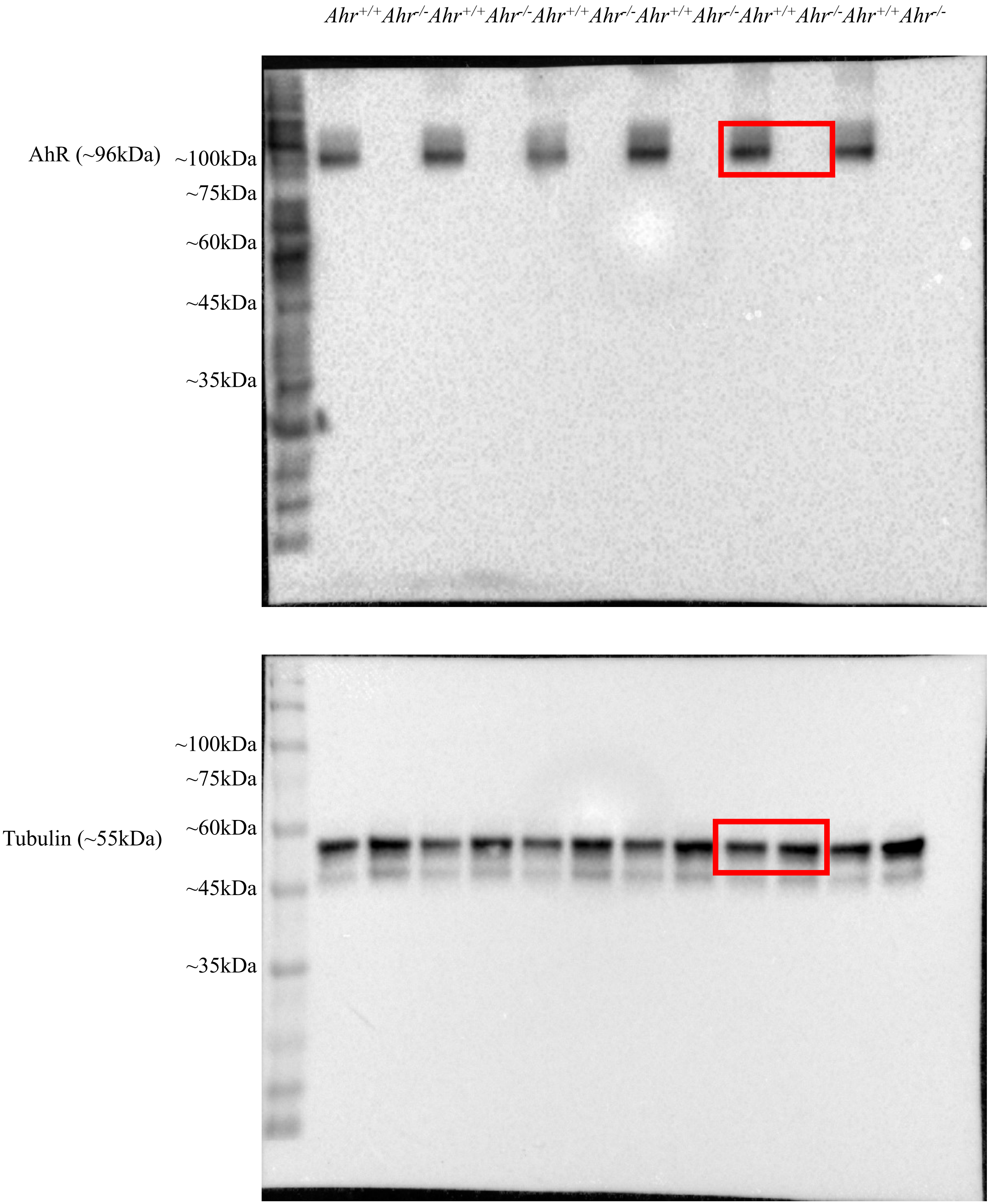

Figure 5A. PTEN

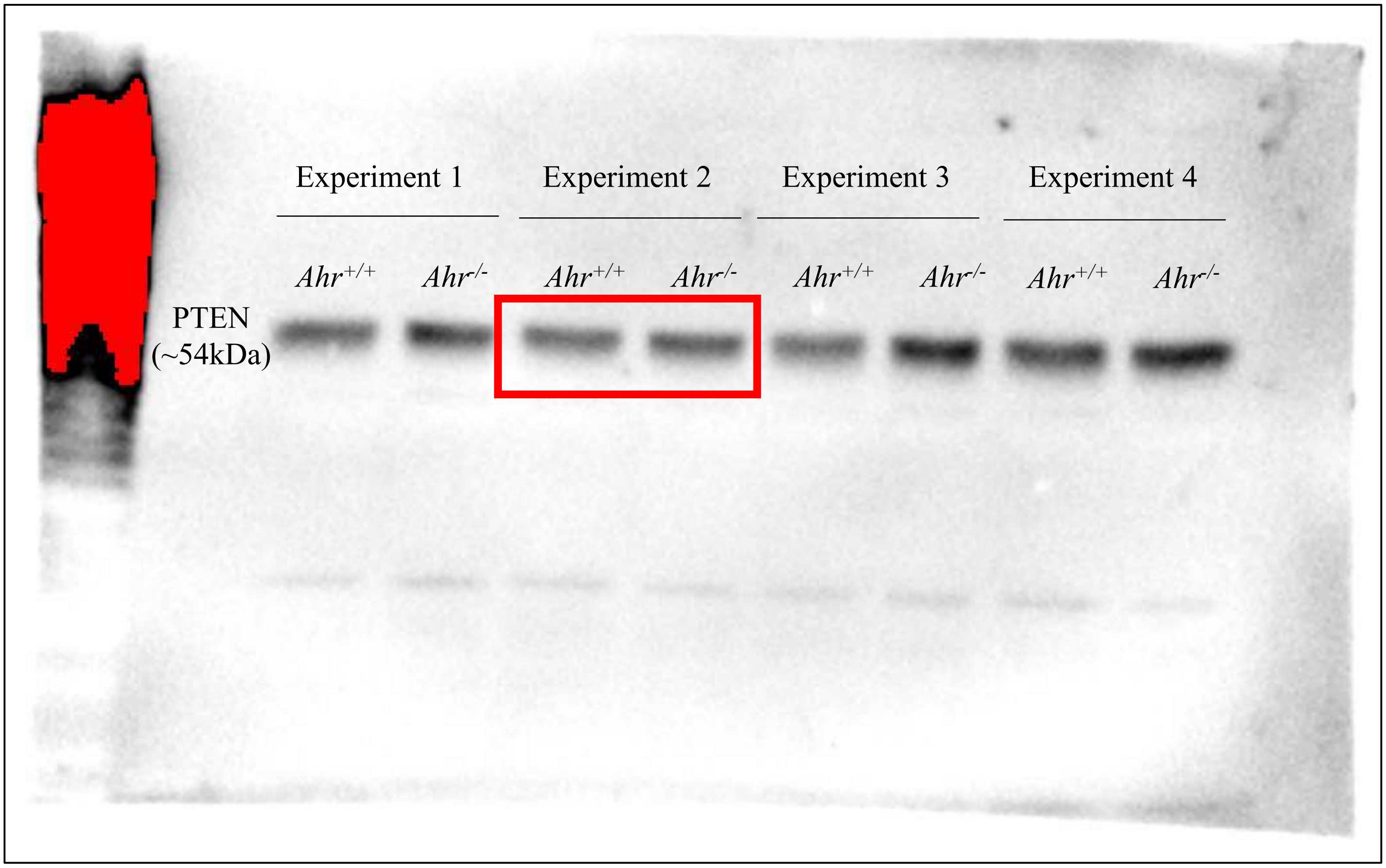

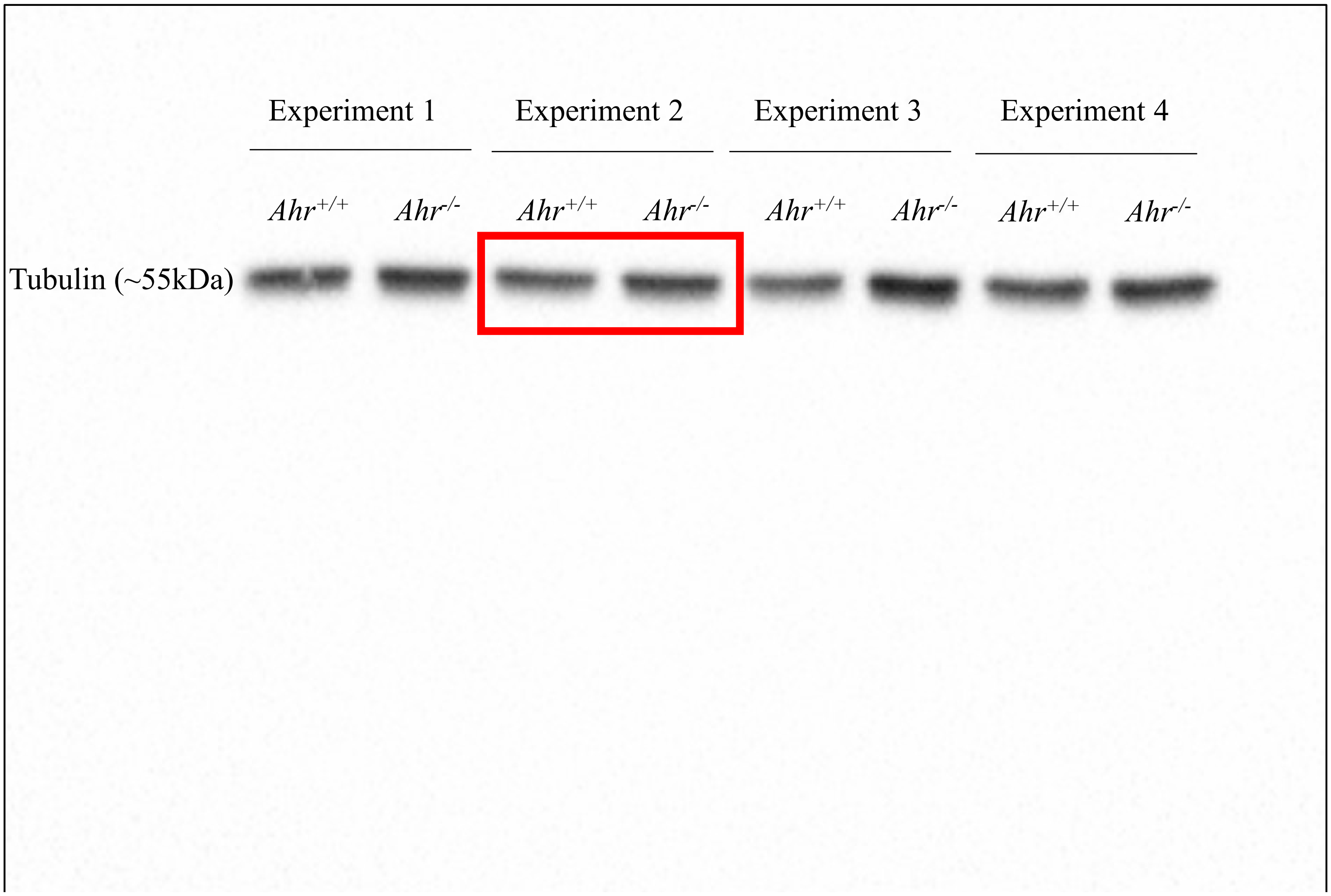

Figure 5A. PTEN (Second exposure)

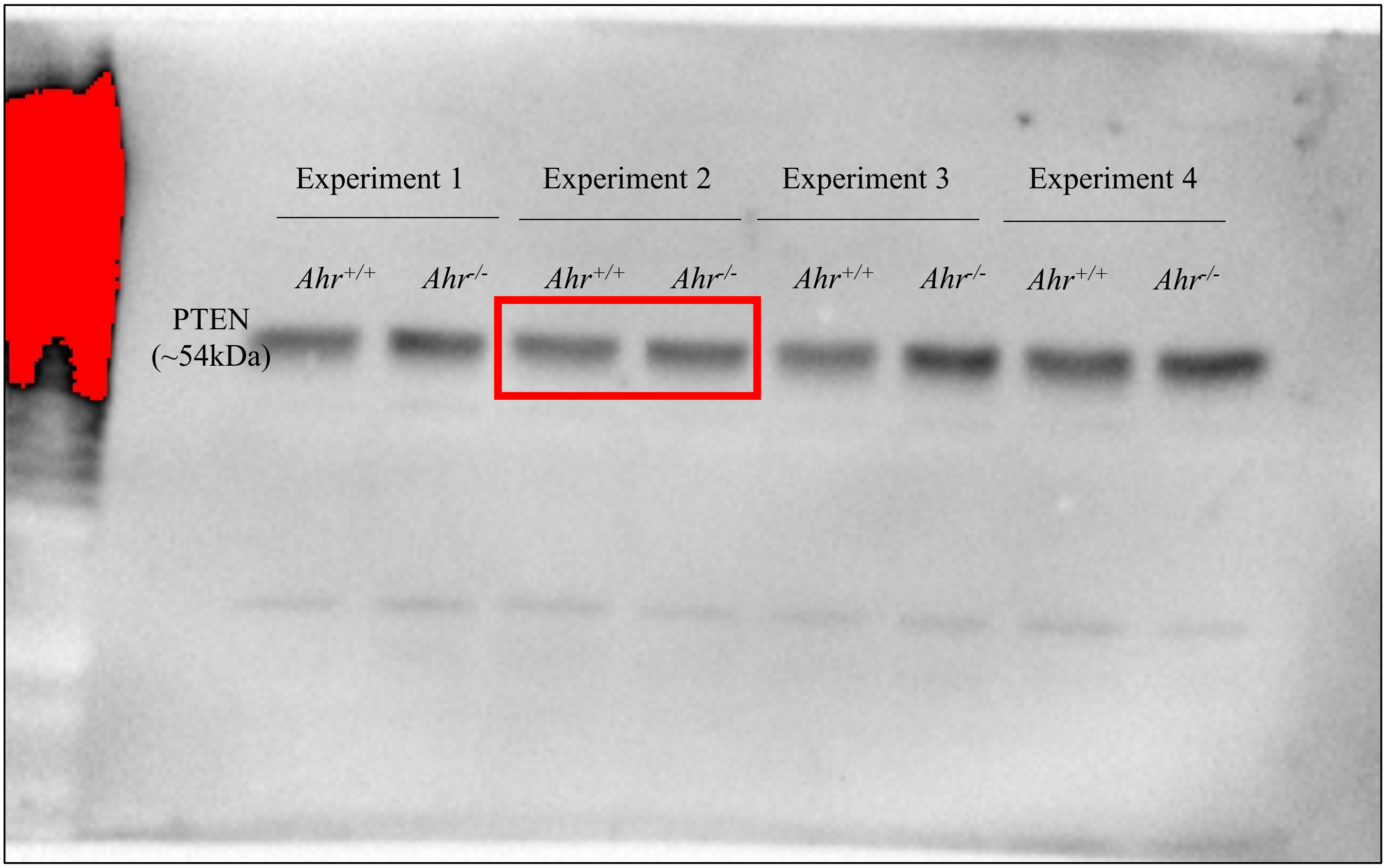

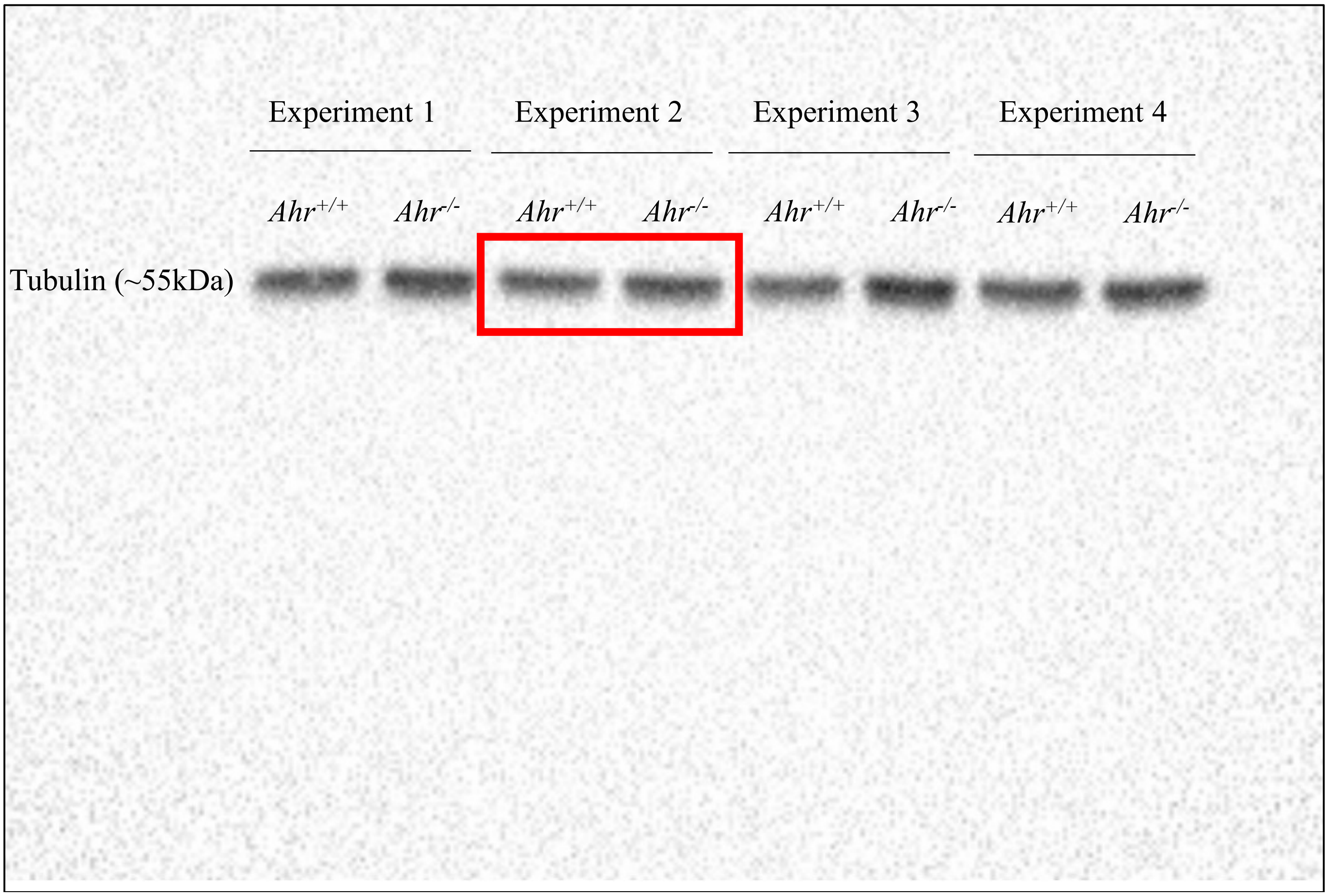

Figure 5A. PTEN (protein ladder)

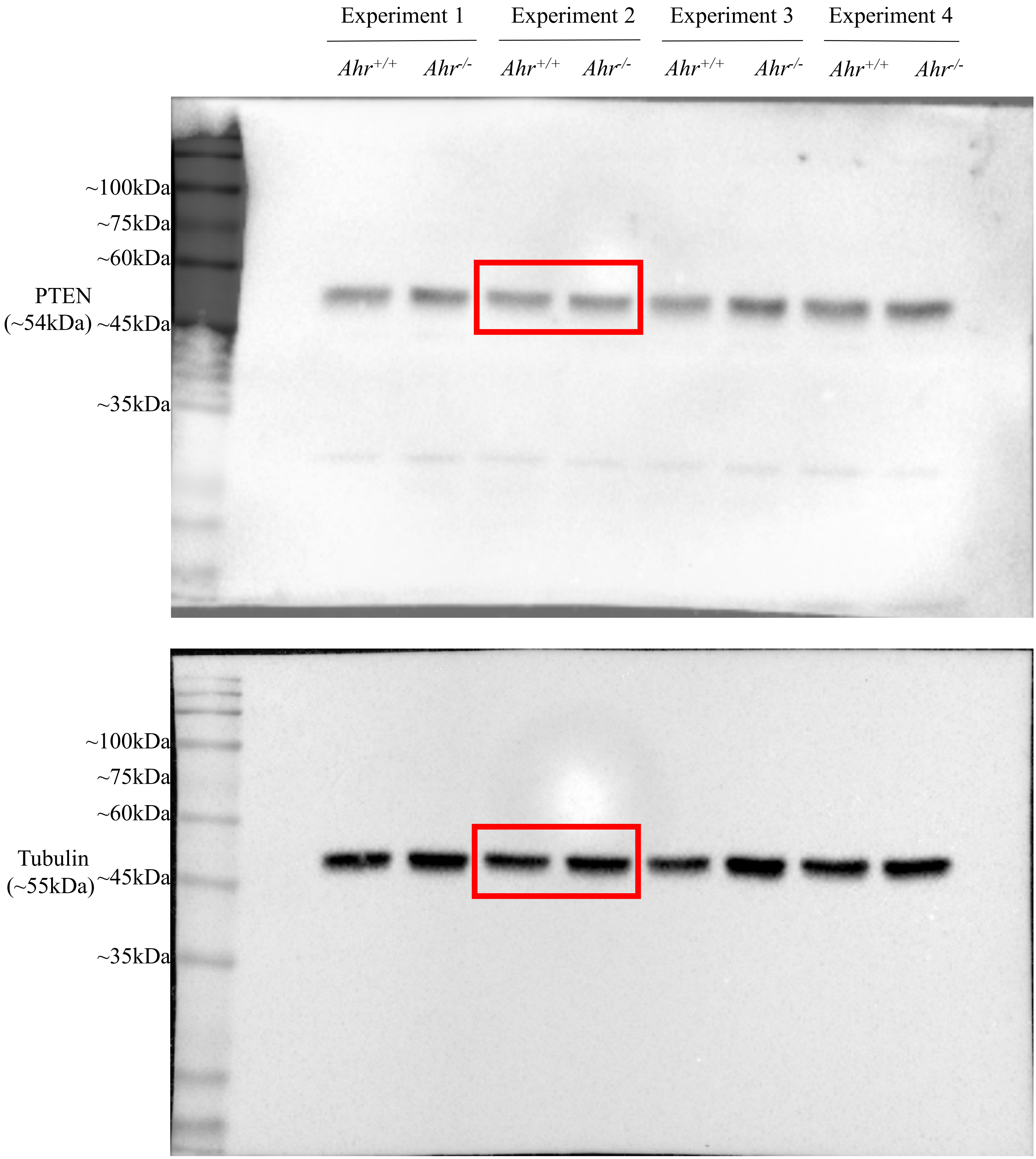

Figure 5B. GSH-MEE

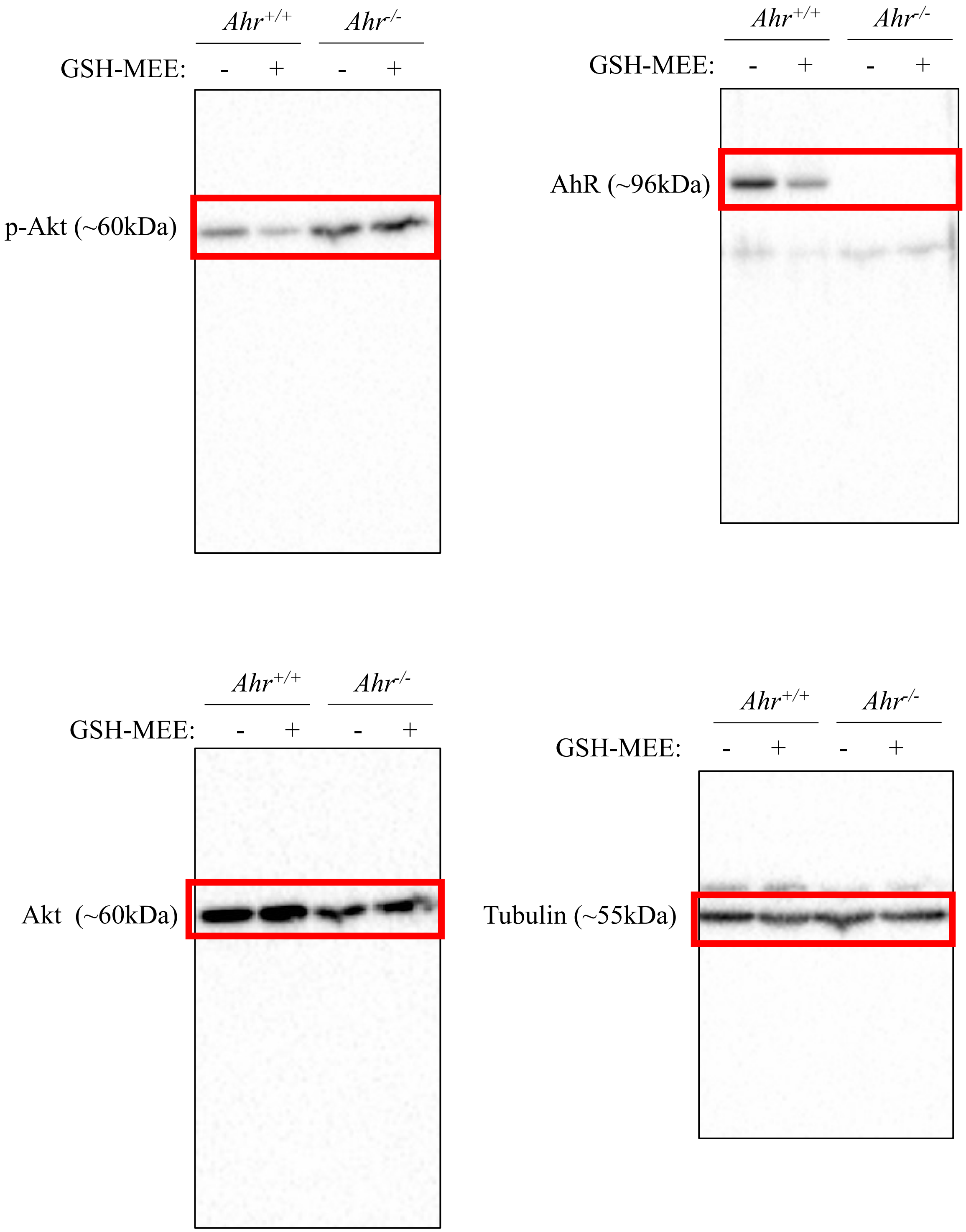

Figure 5B. GSH-MEE (second exposure)

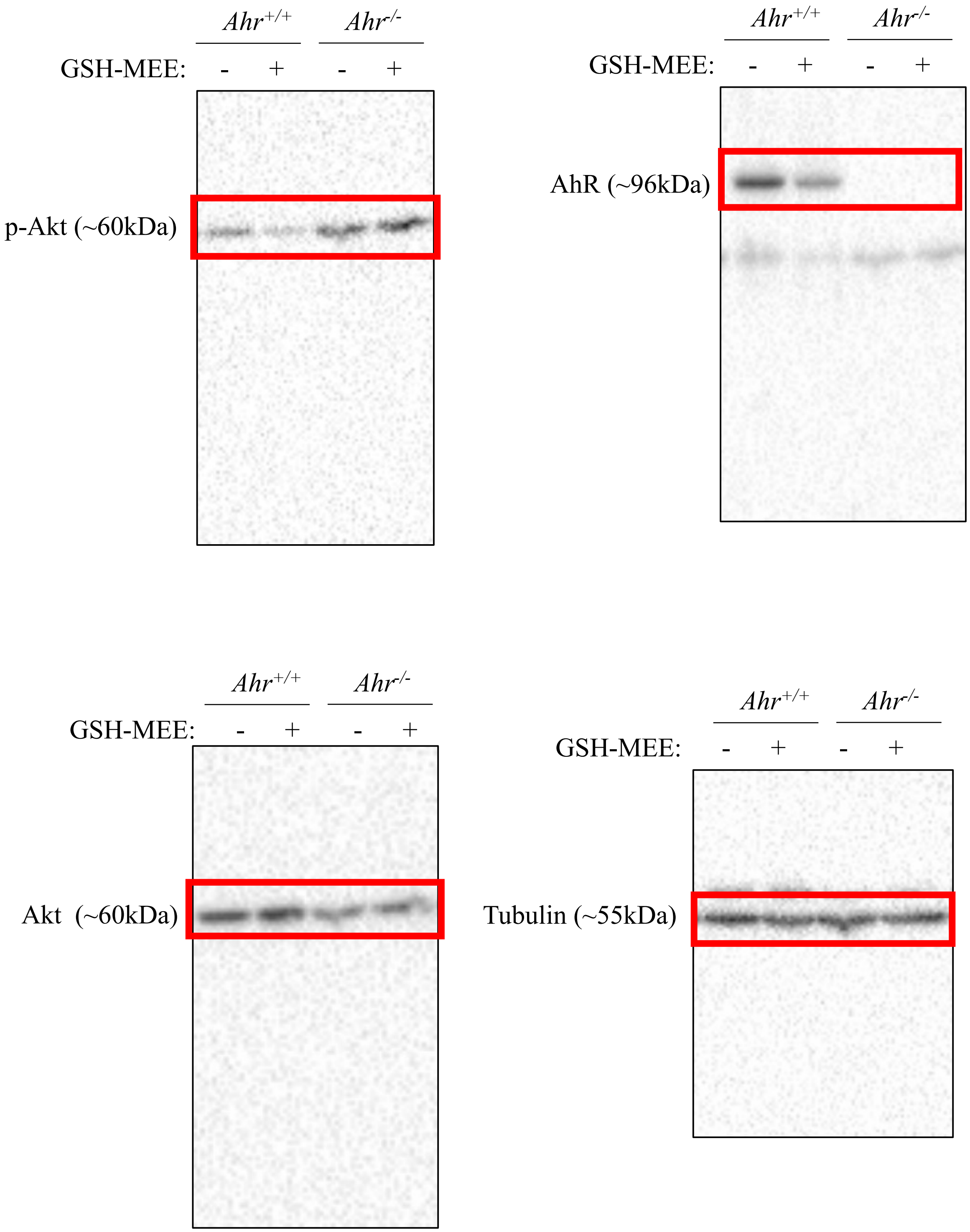

Figure 5B. GSH-MEE (protein ladder)

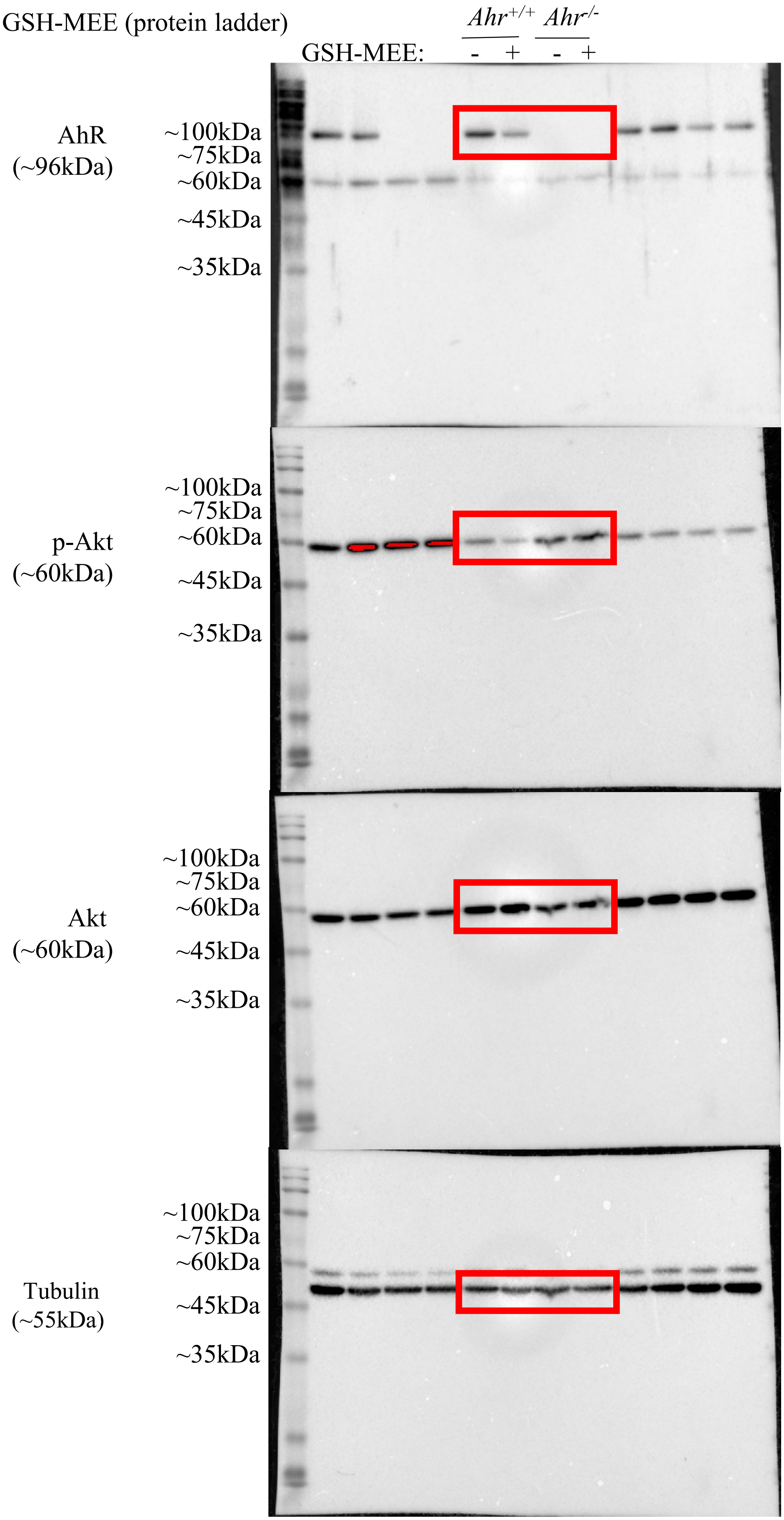

Figure 5C. P85α (right = second exposure)

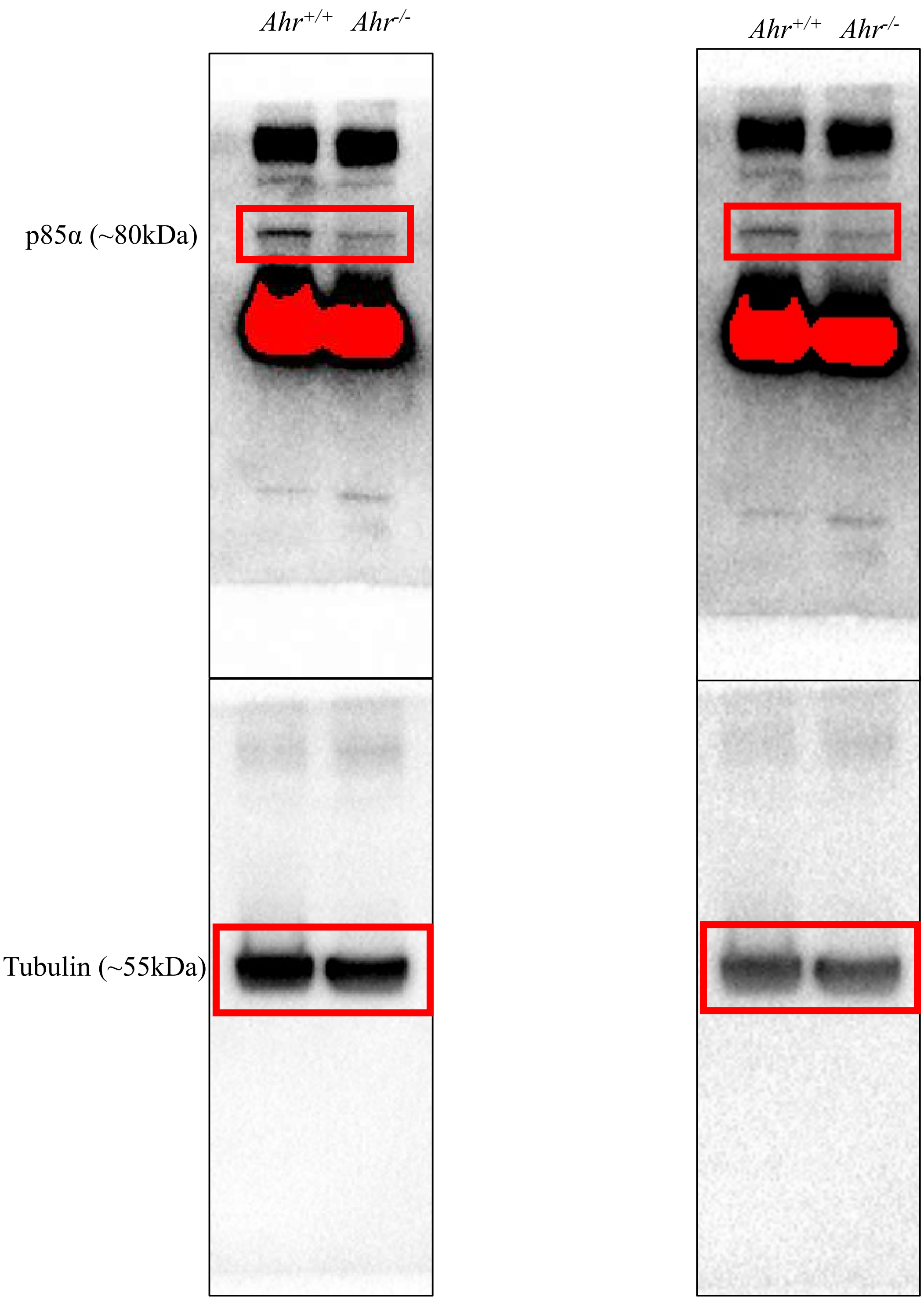

Figure 5C. P85α (protein ladder)

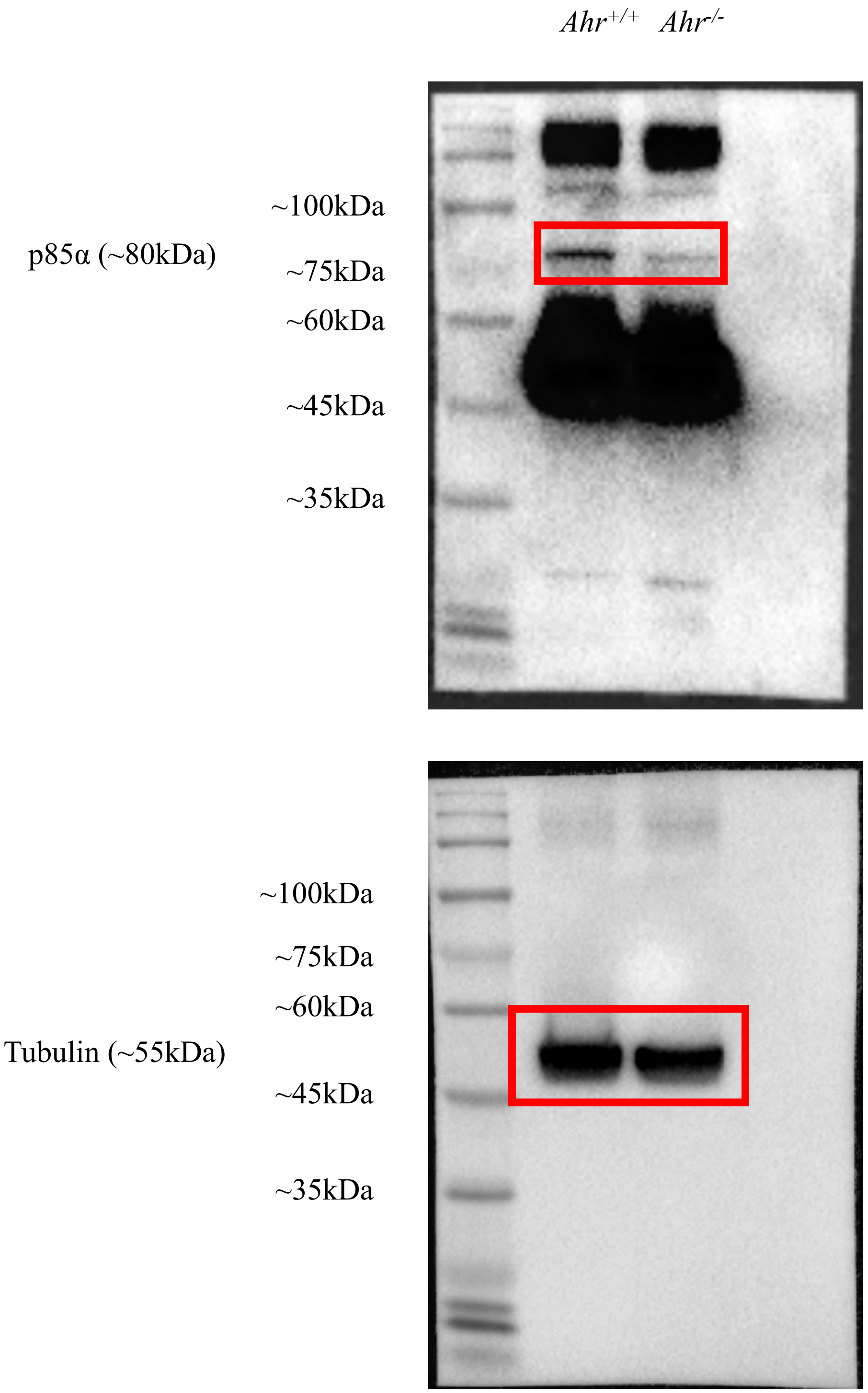

Figure 5D. p85α siRNA

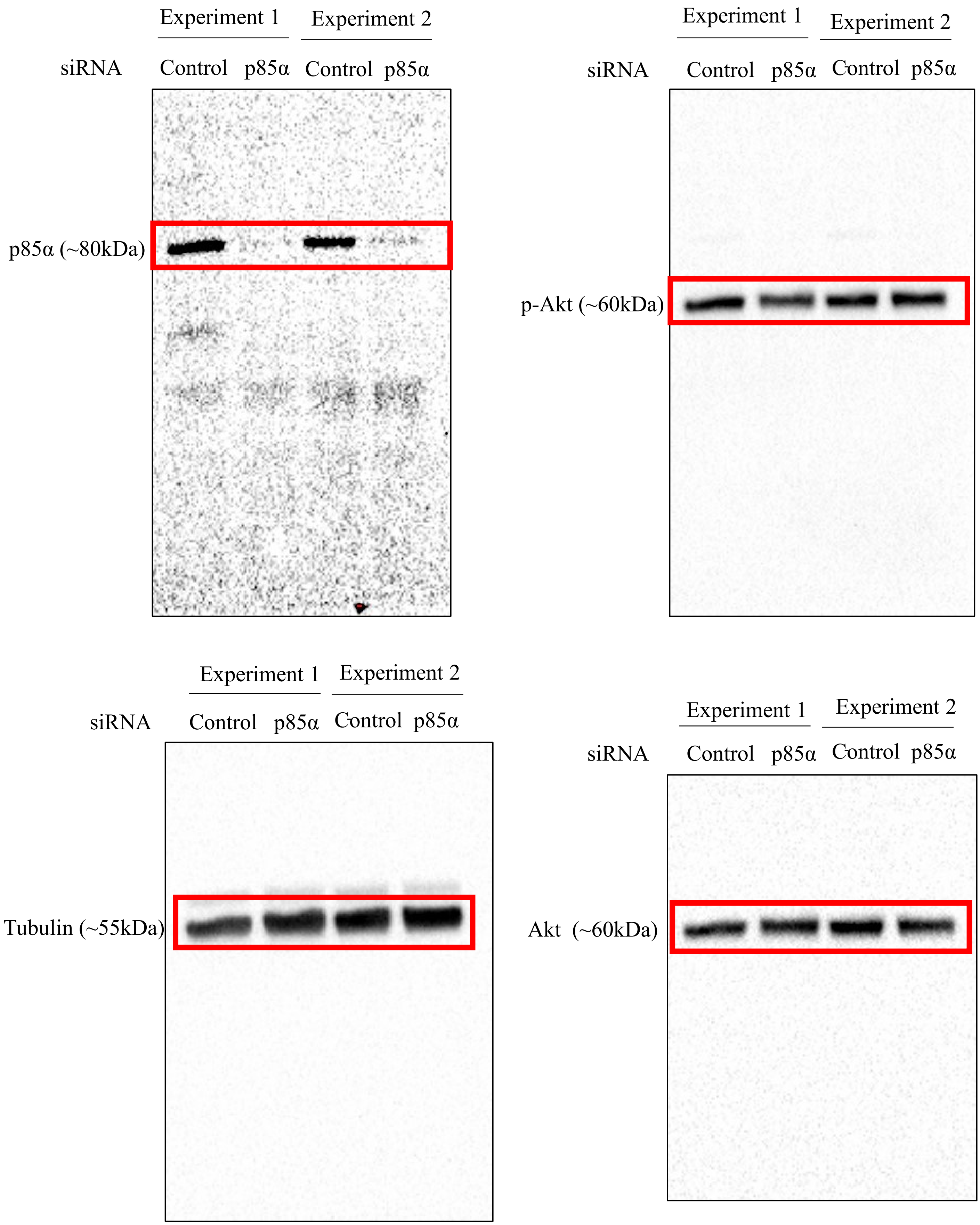

Figure 5D. p85α siRNA (Second exposure)

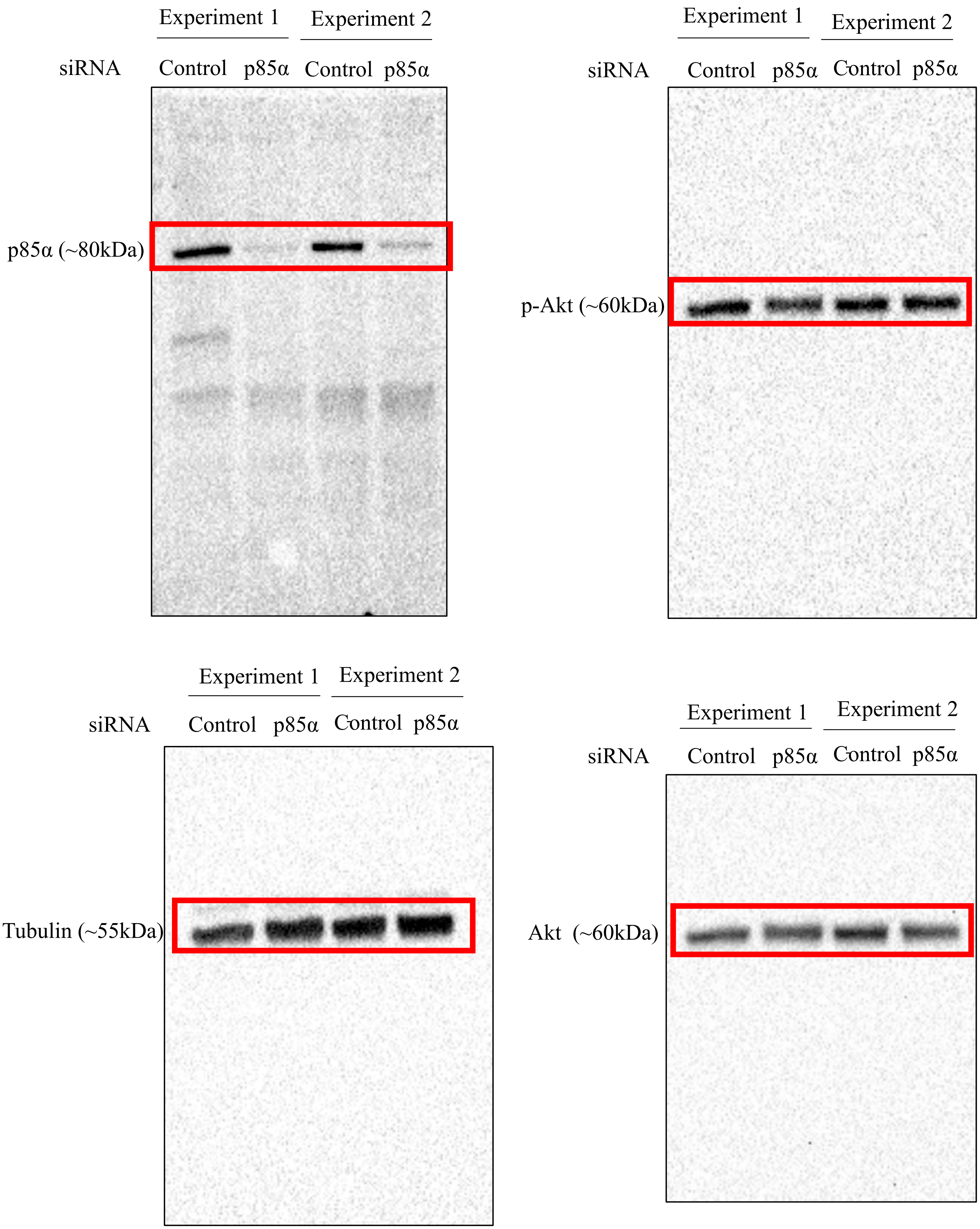

Figure 5D. p85α siRNA (protein ladder)

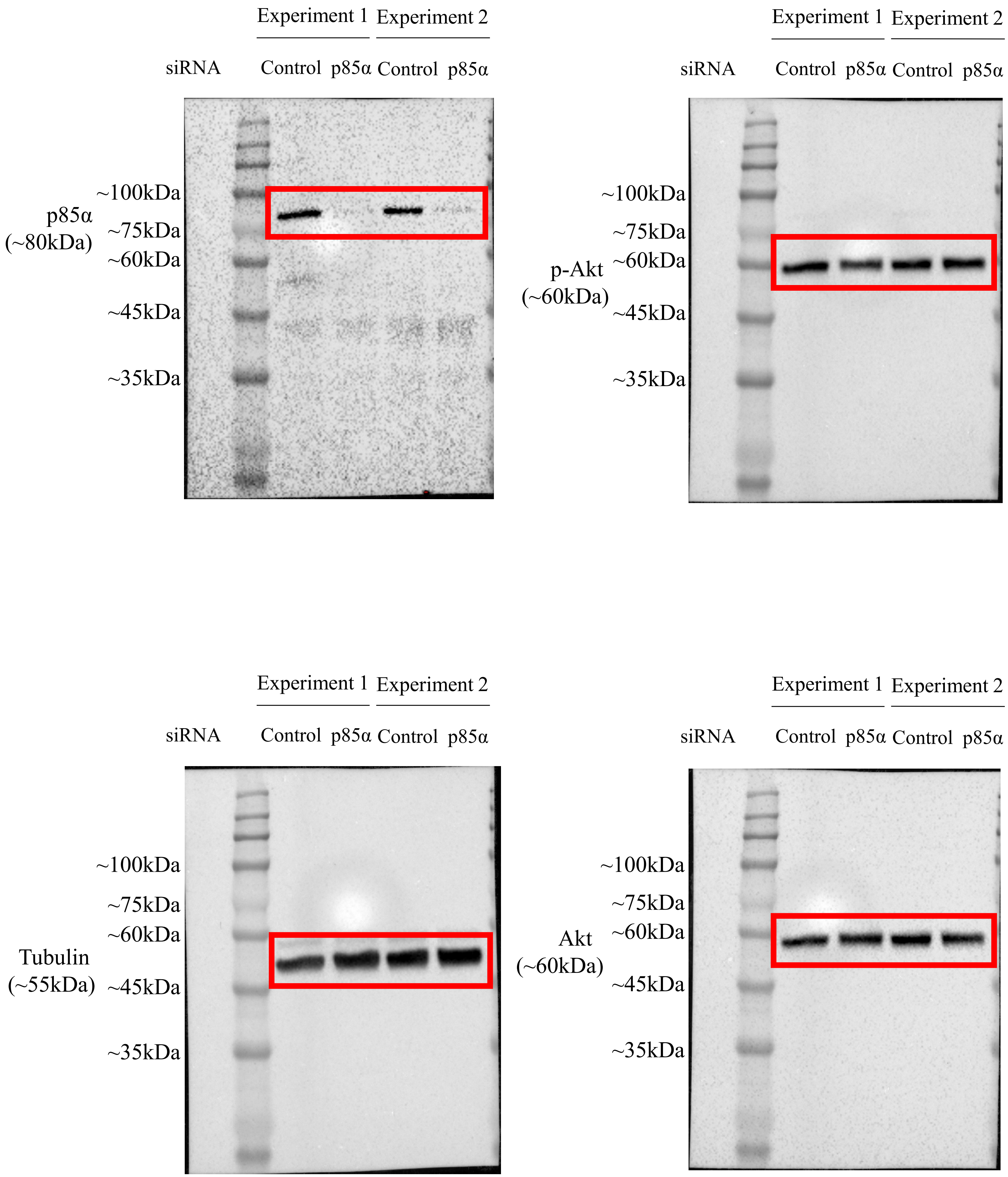

Figure 6A. LY294002: p-Akt

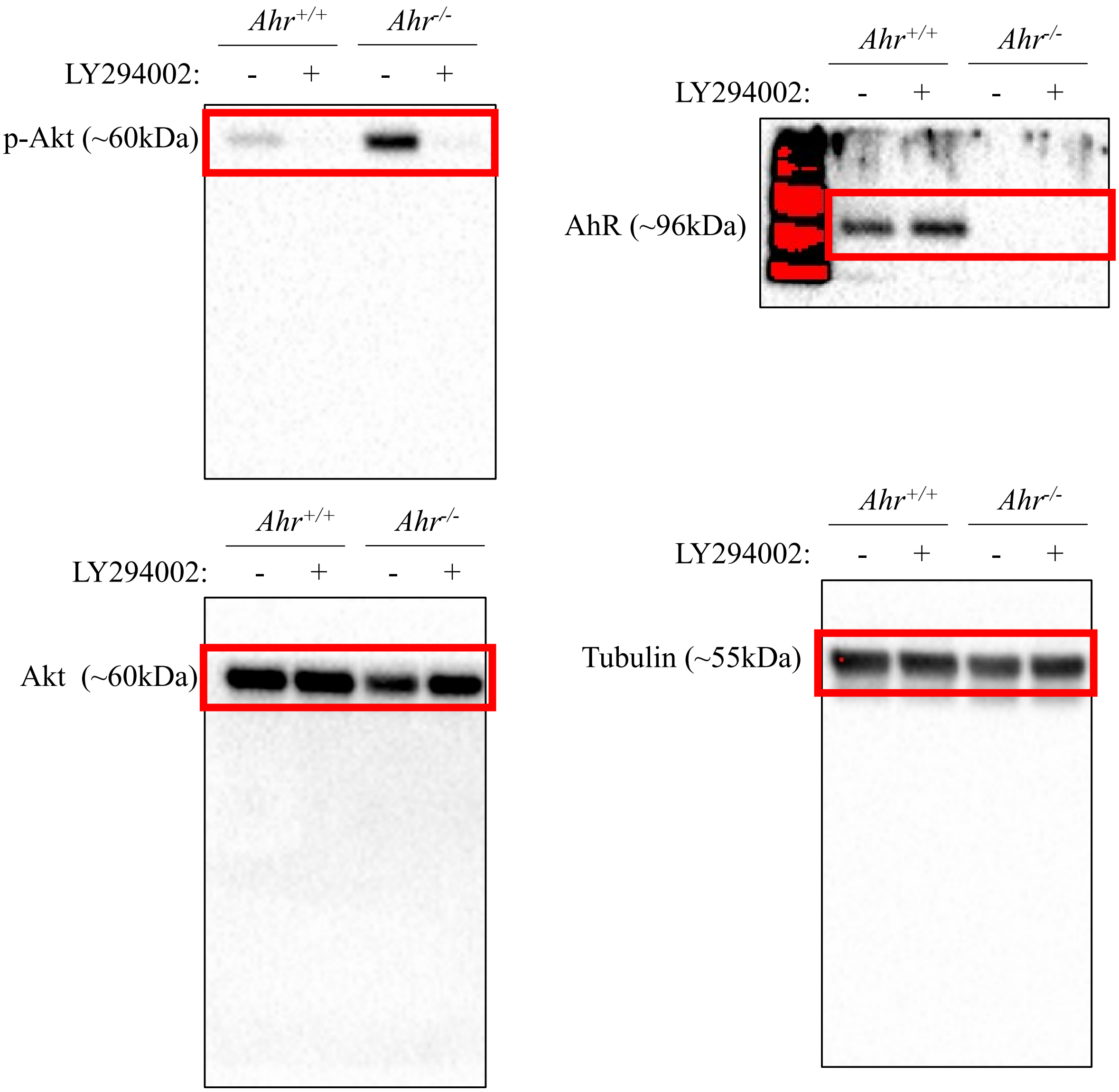

Figure 6A. LY294002: p-Akt (second exposure)

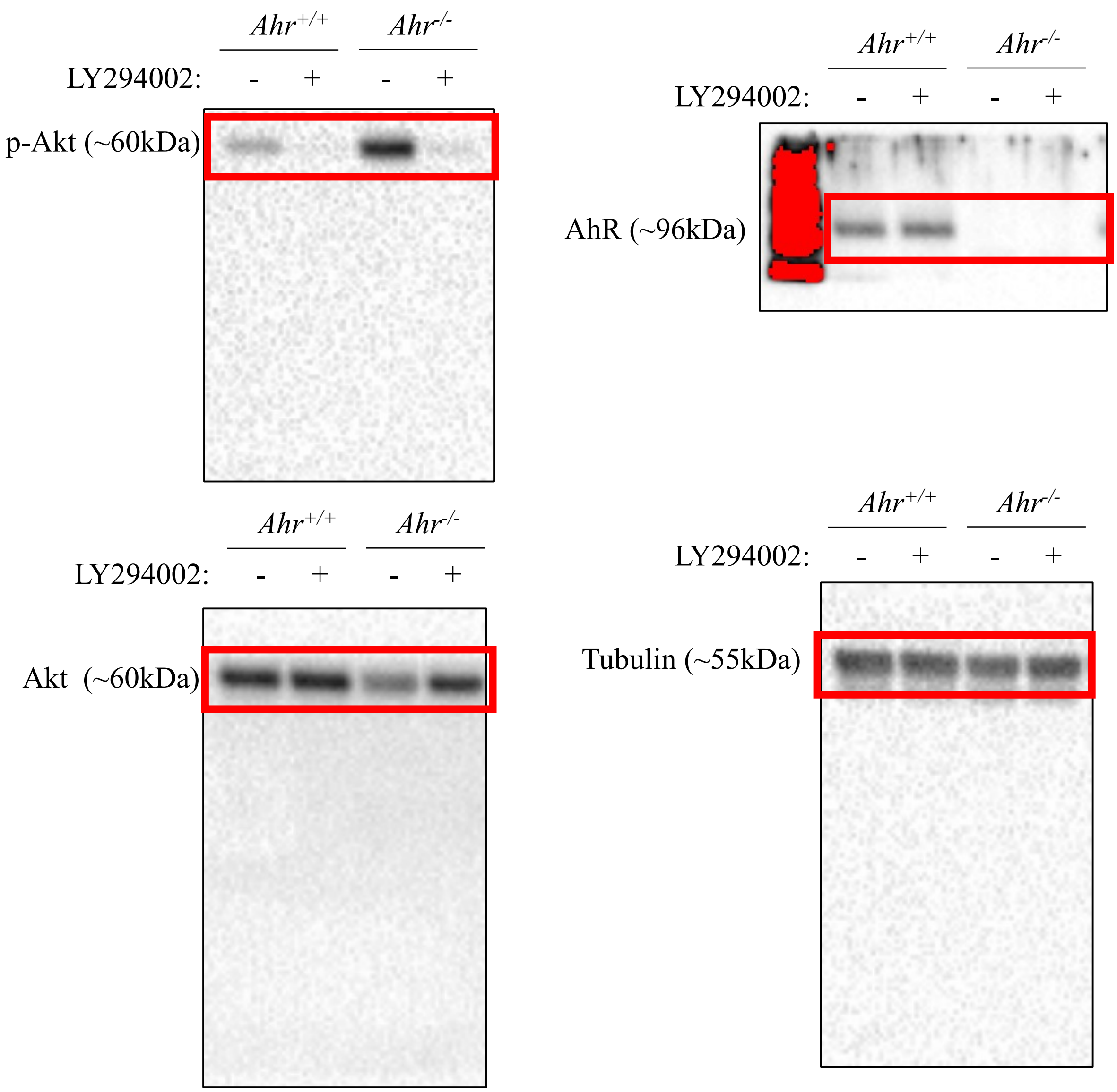

Figure 6A. LY294002: p-Akt (protein ladder)

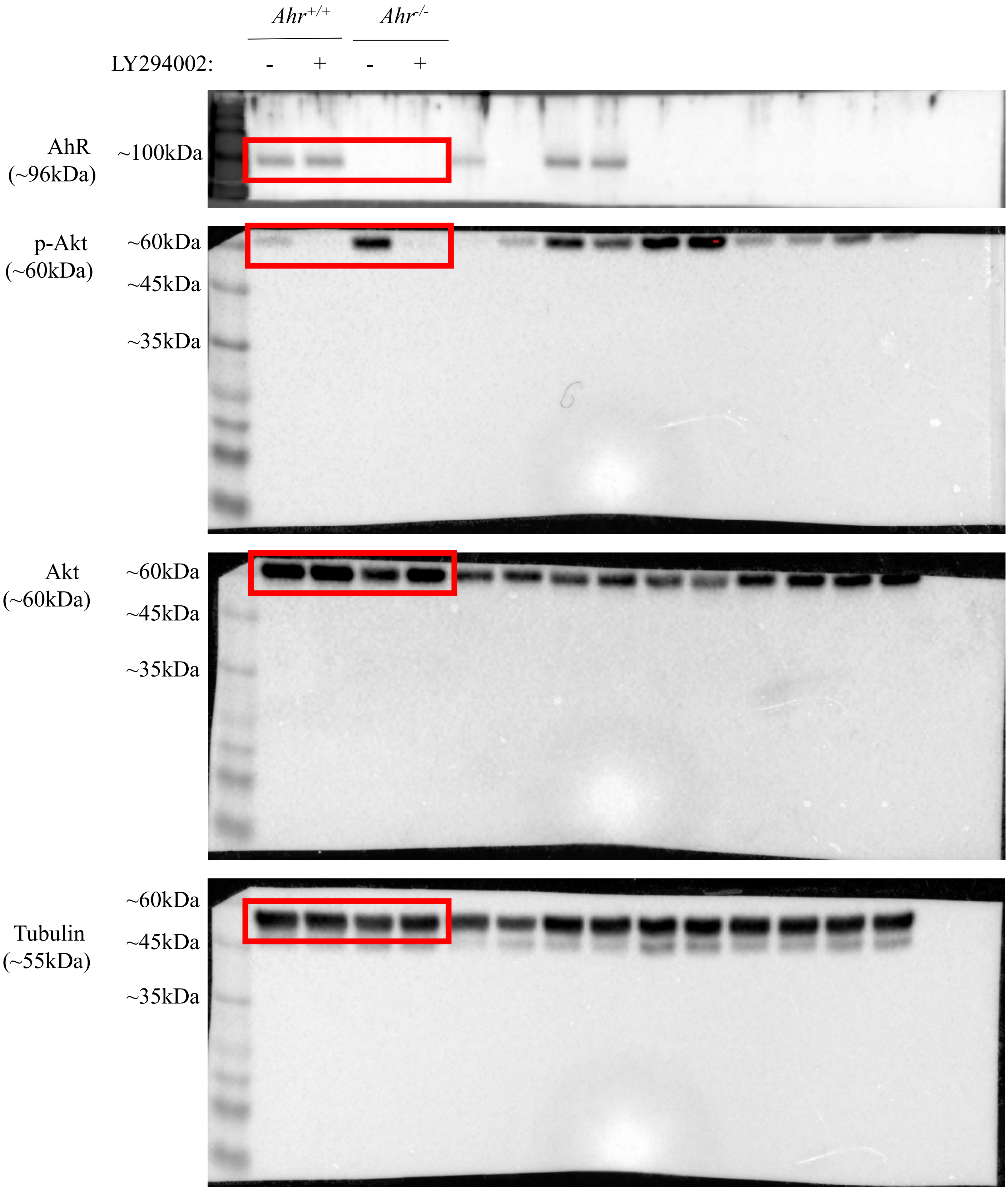

Figure 6B. LY294002: p-GSK3β (right = second exposure)

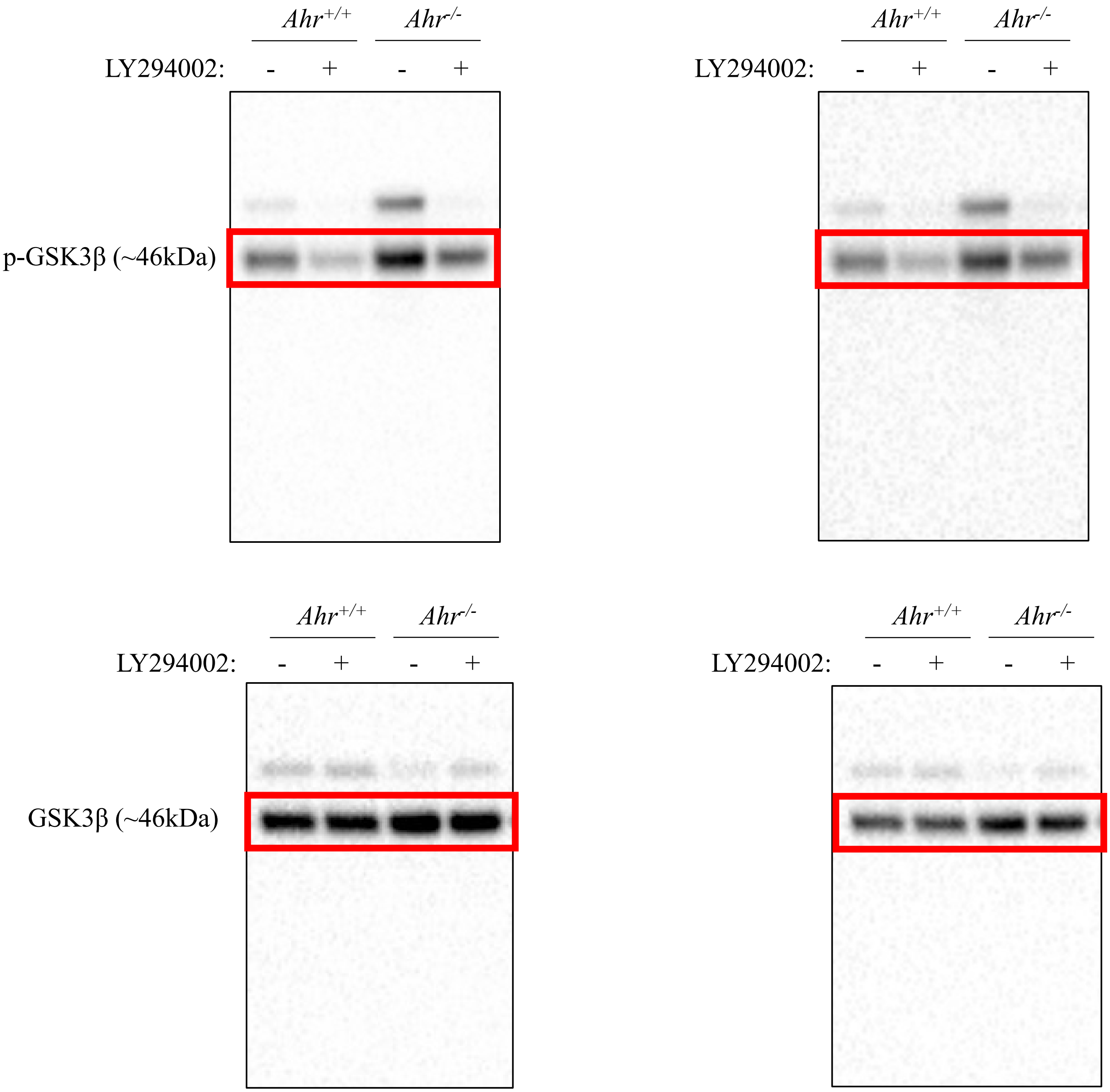

Figure 6B. LY294002: p-GSK3β (protein ladder)

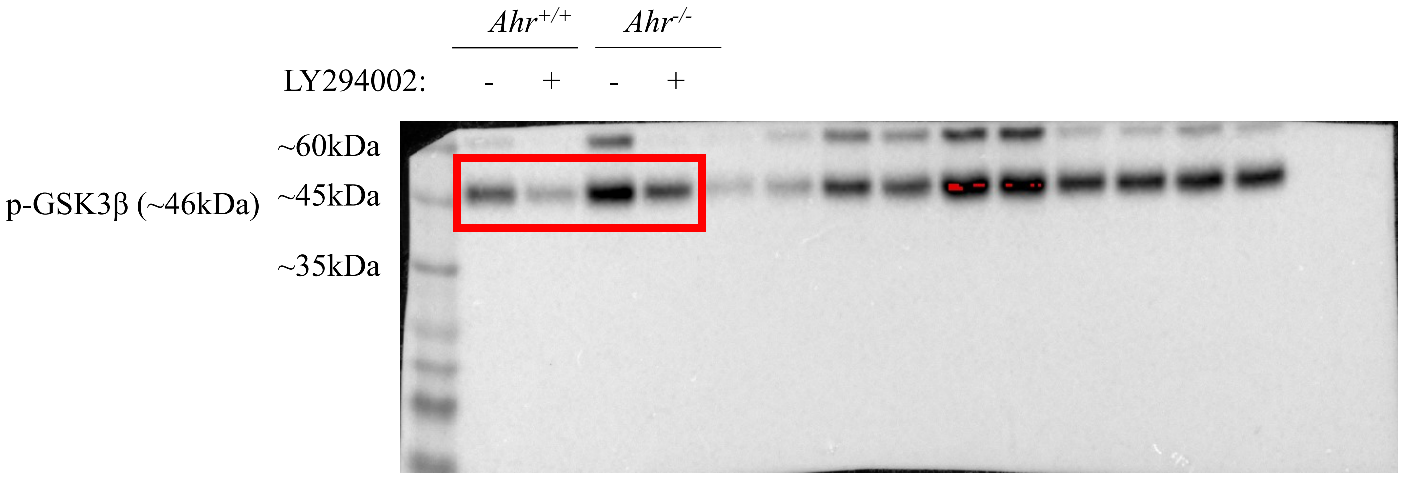

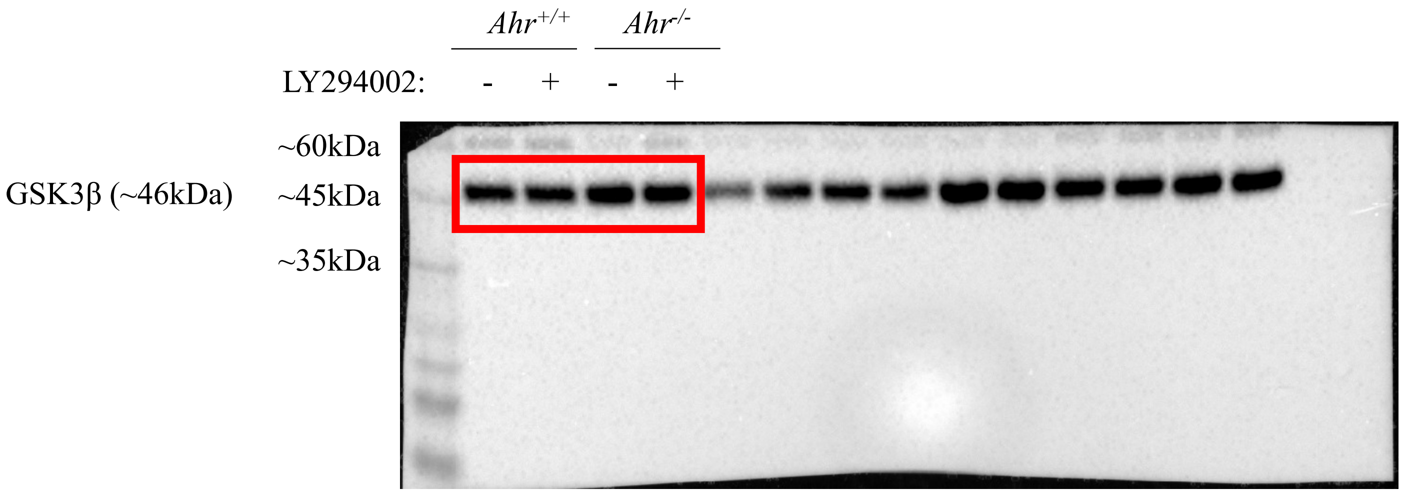

Figure 6C.. LY294002: p-Akt substrates

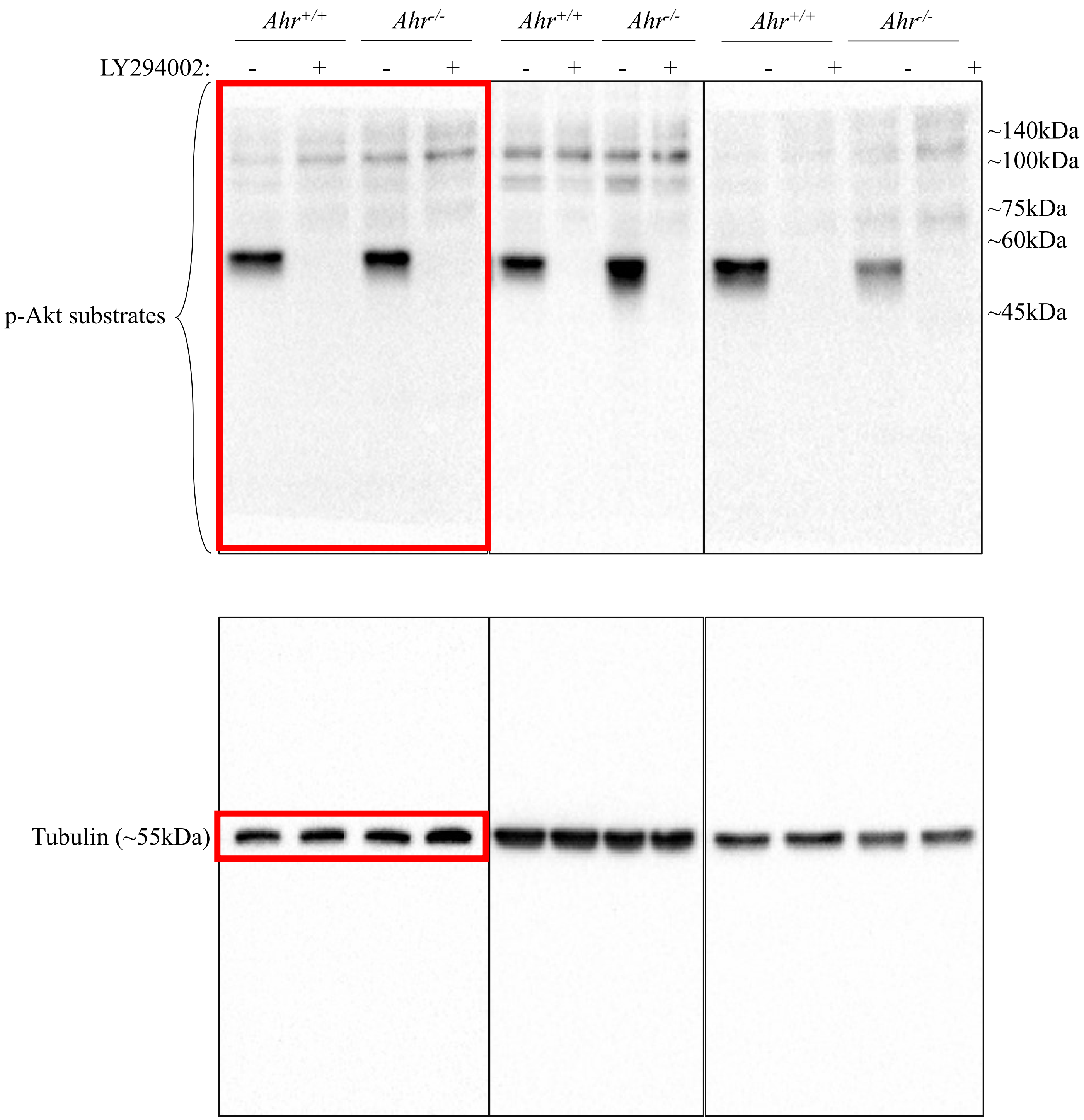

Figure 6C.. LY294002: p-Akt substrates (left = second exposure; right = protein ladder)

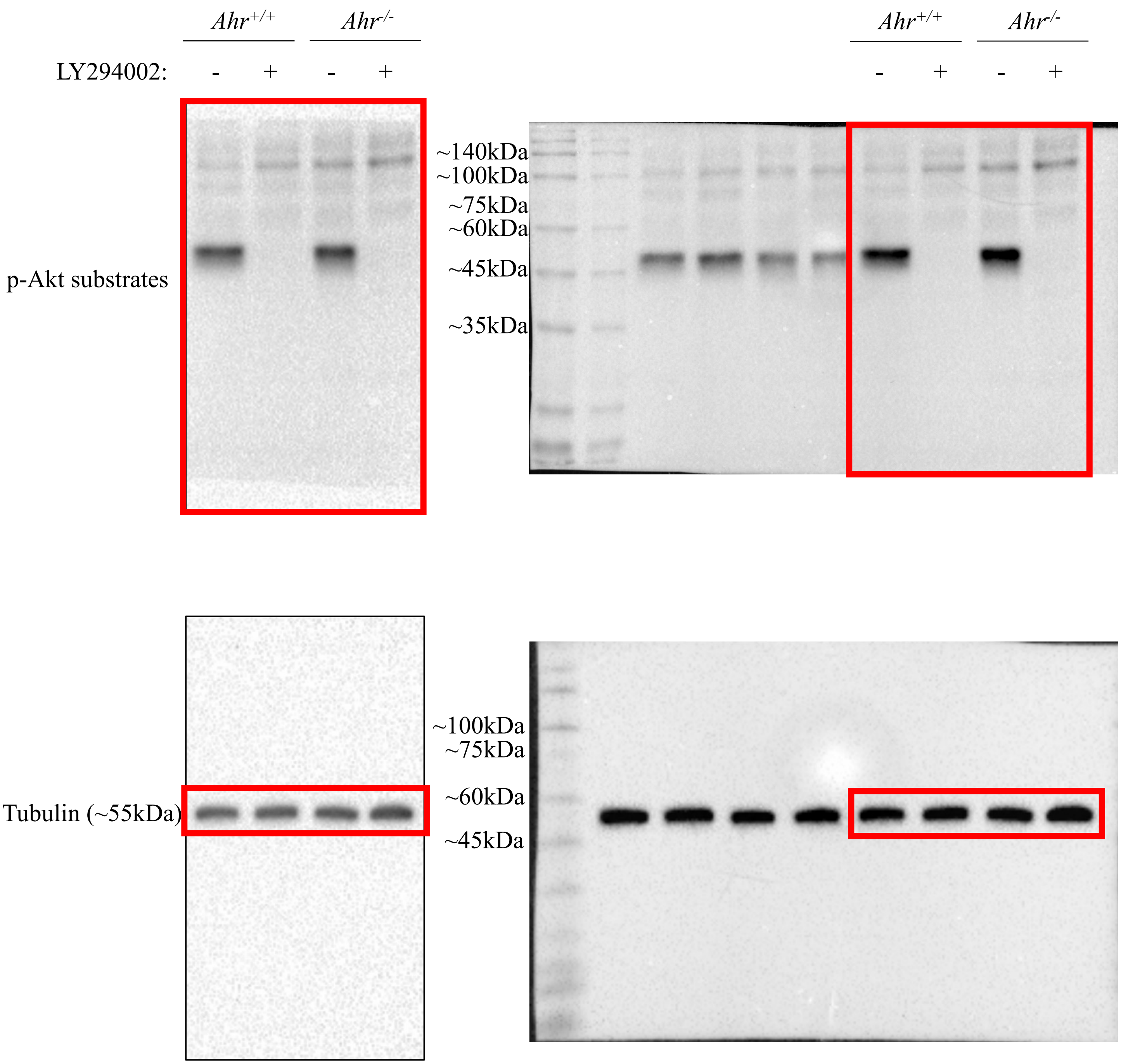

Supplement: Supplementary file 1 — Supplementary Information. [file 41598_2021_2339_MOESM1_ESM.pdf]
